# Novel *N*,*N*′-Disubstituted Selenoureas as Potential Antioxidant and Cytotoxic Agents

**DOI:** 10.3390/antiox10050777

**Published:** 2021-05-14

**Authors:** Gorka Calvo-Martín, Daniel Plano, Ignacio Encío, Carmen Sanmartín

**Affiliations:** 1 Departamento de Tecnología y Química Farmacéuticas, Universidad de Navarra, Irunlarrea 1, E-31008 Pamplona, Spain; gcalvo.3@alumni.unav.es (G.C.-M.); dplano@unav.es (D.P.); 2 Instituto de Investigación Sanitaria de Navarra (IdiSNA), Irunlarrea, 3, E-31008 Pamplona, Spain; ignacio.encio@unavarra.es; 3 Departamento de Ciencias de la Salud, Universidad Pública de Navarra, Avda. Barañain s/n, E-31008 Pamplona, Spain

**Keywords:** selenoureas, antioxidant, cytotoxicity, radical scavenging, selenium

## Abstract

A series of 30 novel *N*,*N* disubstituted selenoureas were synthesized, characterized, and their antioxidant ability was tested using 2,2-diphenyl-1-picrylhydrazyl (DPPH) and 2,2′-azino-bis(3-ethylbenzthiazoline-6-sulfonic acid (ABTS) assays. Additionally, their cytotoxic activity was tested in vitro in a panel of three different cancer (breast, lung and colon) and two normal cell lines. Each selenourea entity contains a para-substituted phenyl ring with different electron-withdrawing and electron-donating groups, and different aliphatic and aromatic nuclei. All of the synthesized selenoureas present antioxidant capacity at high concentrations in the DPPH assay, and three of them (**2b**, **2c** and **2d**) showed greater radical scavenging capacity than ascorbic acid at lower concentrations. These results were confirmed by the ABTS assay, where these novel selenoureas present even higher antioxidant capacity than the reference compound Trolox. On the other hand, 10 selenoureas present IC_50_ values below 10 µM in at least one cancer cell line, resulting in the adamantyl nucleus (**6a**–**6e**), the most interesting in terms of activity and selectivity. Outstanding results were found for selenourea **6c**, tested in the NCI60 cell line panel and showing an average GI_50_ of 1.49 µM for the 60 cell lines, and LC_50_ values ranging from 9.33 µM to 4.27 µM against 10 of these cancer cell lines. To gain insight into its anticancer activity mechanism, we investigated the cell cycle progression of the promising compound **6c**, as well as the type of programmed-cell death in a colon cancer cell line it provokes (HT-29). Compound **6c** provoked S phase cell cycle arrest and the induction of cell death was independent of caspase activation, suggesting autophagy, though this assertion requires additional studies. Overall, we envision that this compound can be further developed for the potential treatment of colon cancer.

## 1. Introduction

Cancer is a group of complex and multifactorial diseases and is considered the third leading cause of death globally. The World Health Organization (WHO) estimates that one-sixth of deaths can be attributed to cancer [1]. Lung (12.3%), breast (12.3%), colorectal (10.6%), and prostate (7.5%) are the types of cancer with the highest incidence worldwide, according to the statistics [2,3]. Several chemotherapy regimens have demonstrated efficacy, but these cancers’ heterogeneity and evolvement of resistance have generated a growing need for new anticancer agents that would sort out these problems.

Selenium (Se) is an essential micronutrient; trace amounts are necessary for the function of the human body and it is mainly obtained through diet and/or nutritional supplements [4]. Se is a component of the selenoproteins (mostly in the form of amino acid selenocysteine) that participate in a wide range of cellular physiological processes [5]. In the human organism, Se acts as an antioxidant (e.g., glutathione peroxidase (GPX)), protecting against the harmful effects of free radicals. Depending on the Se dose and chemical form, diverse effects of this element have been observed on cellular functions such as immunitary response and energy metabolism [6]. A marginal problem of selenium use is its narrow range between the toxic dose and the dose necessary for the proper functioning of living organisms and, for these reasons, each selenium derivative must be studied individually. For example, sodium selenite exhibited excellent activity in the prevention [7] and treatment of different types of cancers, while other derivatives with other chemical forms, such as sodium selenate, did not exert these properties [8]. On the other hand, different studies and clinical trials have supported that Se could prevent cancer [9]. In addition, it has been reported that low Se intake is associated with cancer risk, in spite of data on the anticancer properties of Se not being fully understood [10]. For these reasons, the incorporation of Se into small molecules, both organic and inorganic, has attracted special attention among the researchers, and it is now considered a promising candidate in the field of drug discovery for cancer therapy [11,12].

The anticancer effect of selenium compounds may be asserted through various pathways in the cell [13]. They have been reported to act protectively against oxidative injury by stimulation of DNA repair, by the regulation of inflammatory and immune responses, by the induction of cell cycle arrest, and by provoking cell death by different processes such as apoptosis, necrosis, autophagy, ferroptosis, necroptosis, entosis, anoikis, NETosis, or mitotic catastrophe inhibition of local invasion and migration, as well as blocking angiogenesis, modulating the cell proliferation, etc. [10,14].

Several organoselenium agents have been developed for the inhibition of different types of cancer cell growth. Among them, selenoureas represent a group of simple but well-studied molecules with anticancer and/or antioxidant properties [15,16,17,18,19,20]. In addition, our research team has reported the preparation and cytotoxic and antioxidant activities for different selenourea derivatives [21,22,23,24].

**Figure 1:**
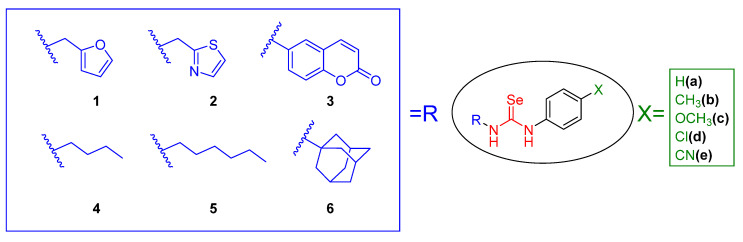
Structural design of selenoureas presented in this work.

With the above facts in mind, and using the fragment-based approach, we designed the compounds presented in this paper. Herein, we pursue the development of new molecules, with the selenourea functioning as cytotoxic agents decorated in the nitrogen atoms with a small set of fragment hits. Therefore, we select nucleus as coumarin [25], furan [26], thiazole [27], and adamantine [28], all of them with reported antitumoral and/or antioxidant activity. In the opposing nitrogen, we enclose the phenyl ring, functionalized with different electron-donating and electron-withdrawing substituents in order to cover a relatively broad range of electronic effects. A general representation of the target compounds is shown in Figure 1.

The cytotoxic activity of these novel selenoureas was screened in vitro at two doses (10 and 50 μM) against a panel of cancer cell lines derived from breast (MCF-7), lung (HTB-54), and colon (HT-29), using a colorimetric assay of 3-(4,5-dimethyl-2-thiazolyl)-2,5-diphenyl-2*H*-tetrazolium bromide (MTT). Ten of these selenoureas were chosen by considering their cell growth inhibitory activity, and their IC_50_ values were determined towards breast (MCF-7), colon (HT-29, HCT-116), and lung (HTB-54) cancer cells, along with a breast non-malignant derived cell line (184B5) and a lung non-malignant derived cell line (BEAS-2B) to determine their selectivity. The radical scavenging activity for all the analogs was assessed in vitro using colorimetric assays of 2,2-diphenyl-1-picrylhydrazyl (DPPH) and 2,2′-azino-bis(3-ethylbenzthiazoline-6-sulfonic acid) (ABTS) at different times and concentrations.

## 2. Material and Methods

### 2.1. Chemistry

#### 2.1.1. General Information

All the chemical reagents for the synthesis were purchased from E. Merck (Darmstadt, Germany), Scharlau (F.E.R.O.S.A., Barcelona, Spain), Panreac Química S.A. (Montcada i Reixac, Barcelona, Spain), Sigma-Aldrich Química, S.A. (Alcobendas, Madrid, Spain) and Acros Organics (Janssen Pharmaceuticalaan 3a, 2440 Geel, Belgium). TLCs were performed on aluminum pre-coated sheets (E. Merck Silica gel 60 F254). Silica gel 60 (0.040–0.063 mm) 1.09385.2500 (Merck KGaA, 64271 Darmstadt, Germany) was used for Column Chromatography. Melting points were determined using a Mettler FP82 + FP80 apparatus (Greifense, Switzerland) and was not corrected. ^1^H-, ^13^C- and ^77^Se-NMR spectra were registered on a Bruker Avance Neo 400 MHz in CDCl_3_ and DMSO-*d*_6_, operating at 400, 100 and 76 MHz, respectively, using TMS as the internal standard. Chemical shifts are reported in δ values (ppm) and *J* values are reported in hertz (Hz). The IR spectra were obtained on a Thermo Nicolet FT-IR Nexus spectrophotometer with KBr pellets.

#### 2.1.2. General Procedure for the Preparation of Selenourea Derivatives

The synthetic route of some of the key starting materials has been described in previous studies. Briefly, the selenocyanates were prepared in two steps. The first one involved formylation of the amines [29] to yield the corresponding formamides (**Ia**–**e**), followed by the treatment with triphosgene and elemental selenium in methylene chloride under reflux in presence of triethylamine to obtain isoselenocyanates (**IIa**–**e**) [30]. Isoselenocyanates were purified by silica gel column chromatography using *n*-hexane or *n*-hexane/ethyl acetate as eluents. The IR spectra easily inform about the presence of the isoselenocyanate functional group (–NCSe). The stretching frequency was observed at 2115–2224 cm^−1^. The 30 final products (**1a**–**e**–**6a**–**e**) were obtained by reaction of isoselenocyanates with different substituted amines in methylene chloride, or tetrahydrofurane at room temperature. The structures of the newly synthesized derivatives were confirmed under the basis of spectral and HRMS analysis, which were in full agreement with the postulated structures.

### 2.2. Radical Scavenging Activity

From all the methods available for the prediction of the antioxidant properties, DPPH**^●^** and ABTS^●+^ assays were chosen according to their simplicity, accessibility, and effectiveness for a fast prediction of radical scavenging activities in vitro. The radical scavenging capacity of the novel selenoureas was determined by DPPH**^●^** and ABTS**^●^**^+^ assays. Measurements were recorded on a BioTeck PowerWave XS spectrophotometer (BioTeck Instruments, Winooski (VT), USA) and the data were collected using KCJunior software (BioTeck Instruments, Winooski (VT), USA).

#### 2.2.1. DPPH Radical Scavenging Assay

A DPPH assay was performed, as described by Svinyarov [31]. Stock solutions of 1 mg/mL were initially prepared for each selenourea in methanol and this solution was used for the preparation of the different concentrations employed in the assay. Ascorbic acid (Asc) was used as positive control due to the literature reports that this derivative is a potent antioxidant and radical scavenger [32]. A volume of 100 µL from a previously prepared stock solution (preserved in dark and daily prepared) of DPPH (100 µM) in methanol were added to 100 µL of the methanolic selenoureas solutions prepared previously, and the antioxidant activity these compounds was assessed by its ability to decolorize DPPH**●** radical (purple color in methanol) to DPPHH (colorless). Thus, radical scavenging activity was estimated by the decrease in the absorbance at 517 nm. Determinations were recorded at different time-points. All the measurements were carried out in triplicate. Results are expressed as the percentage of the radicals scavenged, calculated using the following formula:

(1) $$\%\;\mathrm{DPPH}\;\mathrm{radical}\;\mathrm{scavenging}=\frac{\mathrm{Acontrol}\;-\;\mathrm{Asample}}{\mathrm{Acontrol}}\;\times \;100$$

where A_control_ refers to the absorbance of the negative control and A_sample_ refers to the absorbance of the tested compounds. Results are expressed as percentage of DPPH radical scavenging ± SD.

#### 2.2.2. ABTS Radical Scavenging Assay

As well as DPPH, ABTS radical scavenging assay is a colorimetric method [33]. ABTS (2,2′-azino-bis(3-ethylbenzothiazoline-6-sulfonic acid) was dissolved in deionized water at 1 mg/mL and oxidized to ABTS^●+^ with potassium persulfate (2.45 mM final concentration). This oxidation process is not instantaneous, and the reaction mixture was kept in the dark overnight at room temperature. After ABTS^●+^ radical was generated, the ABTS^●+^ solution was diluted with 50% ethanol until an absorbance of 0.70 ± 0.02 at 734 nm for measurements was achieved. Stock solution of 1 mg/mL in absolute ethanol was prepared for each selenourea, and different dilutions were prepared to reach the final concentration, when 20 µL of this solution were added to 180 µL of the diluted ABTS^●+^. The absorbance at 734 nm was recorded using 50% ethanol as blank. Trolox and Asc were used as positive controls. All determinations were carried out in triplicate. Same time intervals as in the DPPH assay were also measured. The ability to scavenge ABTS^●+^ was calculated using the following formula:

(2) $$\%\;\mathrm{ABTS}\;\mathrm{radical}\;\mathrm{scavenging}=\frac{\mathrm{Acontrol}\;-\;\mathrm{Asample}}{\mathrm{Acontrol}}\;\times \;100$$

where A_control_ refers to the absorbance of the negative control and A_sample_ refers to the absorbance of the tested compounds. The results are expressed as percentage of ABTS radical scavenging ± SD.

### 2.3. Biological Evaluation

#### 2.3.1. Cell Culture Conditions

Cell lines were purchased from the American Type Culture Collection (ATCC). MCF-7, HTB-54, BEAS-2B and 184B5 cell lines were grown in RPMI 1640 medium (Gibco) supplemented with 10% fetal bovine serum (FBS; Gibco), 100 units/mL penicillin, and 100 mg/mL streptomycin (Gibco). HT-29 and HCT-116 cell lines was cultured in McCoy’s 5A (Gibco), 10% FBS, 100 units/mL penicillin, and 100 μg/mL streptomycin. Cells were maintained at 37 °C and 5% CO_2_. Culture medium was replaced every three days.

#### 2.3.2. Cell Viability Assay

A cell viability assay of each compound was carried out using an MTT assay [34]. Each selenourea was dissolved in dimethyl sulfoxide (DMSO) at a concentration of 0.01 M. Serial dilutions were prepared with non-supplemented culture medium. Cytotoxic activity of each selenourea was determined at two different concentrations (10 and 50 µM) in MCF-7, HTB-54, and HT-29 cells. In addition, selected compounds were tested at seven different concentrations (0.1, 1, 5, 10, 25, 50, 100 µM) on MCF-7, HT-29, HCT-116, HTB-54, 184B5, and BEAS-2B cells. The number of passages before defrosting the cells was 3, and they were kept under culture up to a maximum of 15.

Briefly, 1 × 10^4^ cells/well were grown in 96-well plates for 24 h. Then, these cells were incubated with either DMSO (negative control) at 5 µM or a different concentration of each tested selenourea for 48 h. A volume of 20 µL of MTT was added 150 min before the termination point to measure cellular viability. After this time, culture medium was removed. The resultant formazan crystals were dissolved in 50 µL of DMSO, and absorbance was measured at 570 nm. Results are expressed as IC_50_, the half maximal inhibitory concentration. This value was calculated using GraphPad Prism 7 software by nonlinear curve fitting. Furthermore, the selectivity index (SI) was calculated as the ratio of the IC_50_ values determined for the non-malignant and the tumoral cells (IC_50_ (184B5)/IC_50_ (MCF-7) or (IC_50_ (BEAS-2B)/IC_50_ (HTB-54). Data were obtained from at least three independent experiments performed in triplicates.

#### 2.3.3. Evaluation of Cell Cycle Progression and Apoptosis in HT-29 Cells

6 × 10^5^ cells/well were grown in 6-well plates for 24 h. Cultures were treated with the corresponding amount of the selected compounds, DMSO (control), or 10 μM camptothecin (positive control). Propidium iodide (PI) was used as DNA stain dye to determine cell cycle distribution. Cells were stored at −20 °C with 70% ethanol for at least 24 h. Then, ethanol was removed, cells were washed with PBS, and stained for 30 min with 500 µL of a solution containing 0.001% triton, 0.2% *w*/*v* RNase, and 0.02% PI.

For the determination of apoptosis, APC Annexin V Apoptosis Detection Kit with 7-AAD from Biolegend^®^ was employed following the staining procedure described by the manufacturer. Samples were analyzed by flow cytometry using a BD Accuri™ C6 Plus Flow Cytometer (Beckton, Dickinson and company, Franklin Lakes (NJ), USA).

## 3. Results and Discussion

### 3.1. Chemistry

**Scheme 1:**
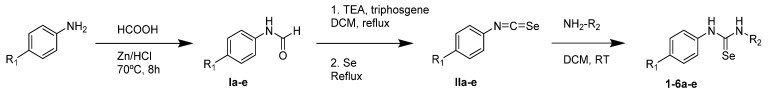
Synthesis of the selenourea derivatives.

As a part of our project aiming for the synthesis of organoselenium-based chemotherapeutic agents, we present the synthesis of a novel family of 30 new selenoureas **1**–**6a**–**e**, following the methodology represented in Scheme 1. In detail, the formamides (**Ia**–**e**) were prepared by reaction between 4-substituted anilines and formic acid, with Zn/HCl as catalyst. The reaction of the corresponding formamides with triphosgene, TEA, and selenium powder yielded the isoselenocyanates (**IIa**–**e**). These intermediates were purified by filtration through celite prior to a silica column chromatography in *n*-hexane or *n*-hexane/ethyl acetate for compound **IIe**. The target selenoureas (**1**–**6a**–**e**) were obtained by reaction of isoselenocyanates, with a variety of amines at room temperature in methylene chloride. The reaction was monitored by TLC and IR by the disappearance of the signal corresponding to NCSe at 2115–2224 cm^−1^. The compounds were isolated by filtration or by solvent evaporation. The resulting residue was washed with *n*-hexane and the desired compounds were obtained with high purity and yield (50–96%). All the structures were confirmed by NMR spectra and high-performance liquid chromatography-mass spectrometry (HPLC-MS).

The characterization of all 30 selenoureas is included in the Supplementary Material, along with the ^1^H-, ^13^C-, and ^77^Se-NMR spectra recorded for each compound (Figures S6–S80). As expected with other selenoureas, ^13^C-NMR shifts of C=Se are placed around 180 ppm, similar to thioureas but at lower fields than ureas, located at around 155 ppm [23]. ^77^Se-NMR shifts of selenoureas can be found between 195 and 315 ppm.

### 3.2. Antioxidant Activity

Oxidative stress is commonly present in several diseases, including cancer. For this reason, antioxidants are preferable agents for preventing these diseases, especially given their ability to inhibit the oxidative damage to DNA caused by scavenging free radicals. On the other hand, the antioxidant activity of selenium compounds at optimal doses is well established, in spite of supranutritional doses displaying prooxidant activity [10,35,36]. Glutathione peroxidases, for example, are a group seleno-containing protein responsible for oxidoreductase activity in the immune system, neutralizing peroxides and preserving the integrity of cell membranes. This activity is proportional to the selenium uptake [37]. Some organic selenocompounds also show a direct radical scavenging activity, specially selenoureas [38,39,40].

#### 3.2.1. DPPH Radical Scavenging Assay

In order to determine the radical scavenging of these newly synthesized selenourea derivatives, the DPPH assay was firstly employed. Measurements were performed at four different concentrations ranging from 0.0003 to 0.03 mg/mL, and collected at time-points between 0 min and 120 min (Figures S81–S100). Results are presented as the percentage of inhibition for DPPH radical scavenging activity.

**Figure 2:**
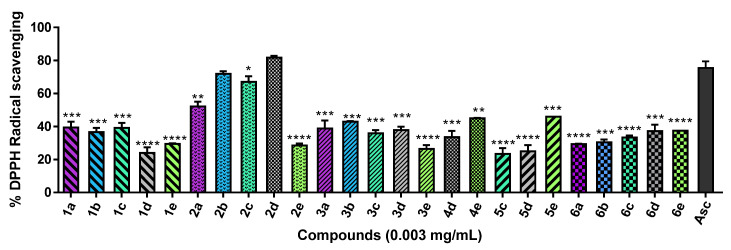
DPPH radical scavenging activity, expressed as percentage, for the selenoureas presented herein. DPPH^●^ scavenging at 0.003 mg/mL after 30 min of treatment. Selenoureas **4a**, **4b**, **4c**, **5a**, and **5b** were degraded before the DPPH assay was realized. Significance: * *p* < 0.05, ** *p* ≤ 0.01, *** *p* ≤ 0.001 and **** *p* ≤ 0.0001 compared with Asc. Results obtained for oher concentrations and incubation times are included in the Supplementary Material (Figures S81–S100).

All tested selenoureas exhibited potent antioxidant activity at 0.03 mg/mL after 30 min, similar to ascorbic acid (Figure S82). Some insights concerning the structural features related to the promising antioxidant activity of these compounds were observed at 0 min (Figure S81). Firstly, it was observed that compounds containing the -CN substituent (**1e**, **2e**, **3e**, **4e**, **5e** and **6e**) and the coumarin moiety (**3a**, **3b**, **3c**, **3d** and **3e**), along with **2a** that presents the thiazol motif, displayed fast kinetics, reaching the maximum activity at time-point 0′. In general, the presence of the aliphatic chains as connectors was detrimental to the activity, with the exception of **4e** and **5e**. No differences were observed with the elongation of this chain from four to six carbon atoms. at concentrations 10-fold lower, 0.003 mg/mL, no compound reached the maximal activity at timepoint 0′; however, coumarin derivatives **3a**–**e** and –CN fragment presented the faster kinetic. That said, at 30- (Figure 2), 60-, 90-, and 120-min derivatives with thiazol scaffold, mainly **2b** and **2d**, resulted the most promising, providing similar antioxidant activity than the reference ascorbic acid. Concentrations of 0.0006 and 0.0003 mg/mL did not provide remarkable results. The compounds displayed their free radical scavenging abilities in a dose- and time-dependent manner. Notably, compounds **4a**, **4b**, **4c**, **5a** and **5b** decomposed before screening, and they could not be tested.

#### 3.2.2. ABTS Radical Scavenging Assay

**Figure 3:**
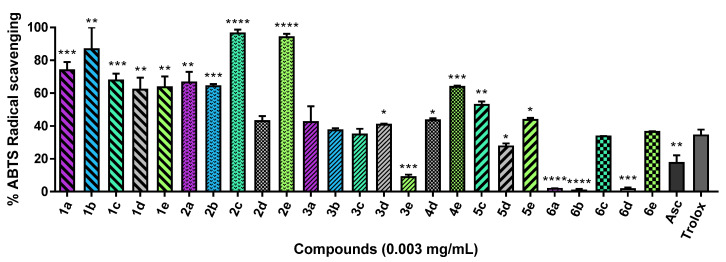
ABTS radical scavenging activity, expressed as percentage, for the selenoureas presented herein. ABTS^●+^ scavenging after 60 min of treatment at 0.003 mg/mL. Selenoureas **4a**, **4b**, **4c**, **5a**, and **5b** were degraded before the DPPH assay was realized. Significance: * *p* < 0.05, ** *p* ≤ 0.01, *** *p* ≤ 0.001 and **** *p* ≤ 0.0001 compared with Trolox. Results obtained for other times and concentrations are included in the Supplementary Material (Figures S101–S106).

In order to confirm the antioxidant activity of the selenoureas, we performed an additional ABTS (2,2′-azino-bis(3-ethylbenzothiazoline-6-sulfonic acid) radical scavenging assay. Following the results obtained in the DPPH assay, this experiment was performed at 0.03 and 0.003 mg/mL. Measurements were recorded after 0′, 6′ and 60′ of incubation with ABTS. For comparison purposes, Trolox and ascorbic acid were used as references. The remarkable results are summarized in Figure 3.

At a concentration of 0.03 mg/mL, all compounds, with the exception of coumarin containing selenoureas **3b**–**e**, reached the maximal ABTS radical scavenging activity quickly, as well as ascorbic acid and Trolox, the reference compounds (Figure S101). At a lower concentration, kinetics were slower. At 0.003 mg/mL, selenoureas needs 60 min to reach their maximal ABTS radical scavenging activity. At this timepoint and concentration, only five selenoureas (**3e**, **5d**, **6a**, **6b**, and **6d**) present significant lower radical scavenging activity than Trolox. However, **2d**, **3a**, **3b**, **3c**, **6c**, and **6e** show similar activity to Trolox, and the rest show a clearly higher scavenging activity in this assay. Remarkable ABTS radical scavenging activity is shown by compounds **2c** and **2e**. These thiazol containing selenoureas have an activity above 90%, significantly higher than ascorbic acid or Trolox.

### 3.3. Biological Evaluation

#### 3.3.1. Cytotoxic Activity

**Figure 4:**
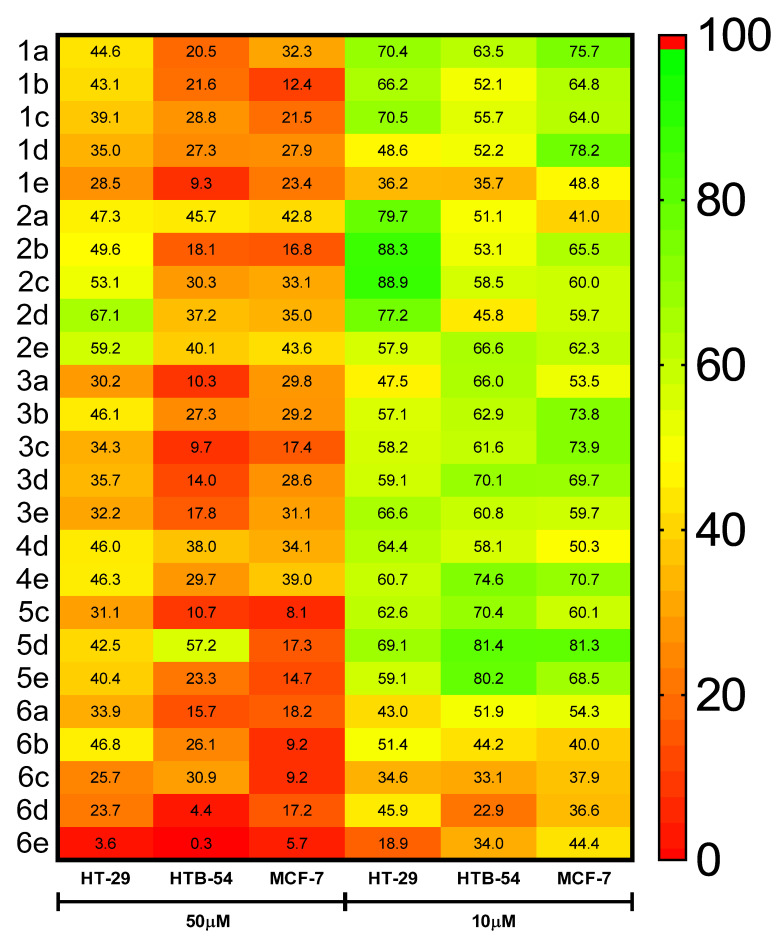
Heat map representation of cell viability, expressed as the percentage of live cells, after 48 h of treatment with each selenourea at 50 µM and 10 µM concentrations in HT-29, HTB-54, and MCF-7 cell lines. Red tones account for low cell survival; green tones account for great cell survival. Selenoureas **4a**, **4b**, **4c**, **5a**, and **5b** were excluded due to their rapid degradation.

The cytotoxic effects of synthesized compounds were evaluated against three human cancer cell lines: breast adenocarcinoma (MCF-7), lung adenocarcinoma (HTB-54), and colon carcinoma (HT-29), using a 3-[4,5-dimethylthiazol-2-yl]-2,5-diphenyltetrazolium bromide (MTT) assay [41]. Firstly, the compounds were screened at two doses (50 and 10 µM) and after 48 h of treatment. The results are summarized in Figure 4 and are presented as percentages of growth inhibition related to control.

Those derivatives that reduced the growth of the cell lines below 50% at 10 µM in at least one cell line were selected for being evaluated at five concentrations between 0.01 and 100 µM. Ten compounds (**1d**, **1e**, **2a**, **2d**, **3a**, **6a**, **6b**, **6c**, **6d**, and **6e**) passed this threshold and four of them (**1e**, **6c**, **6d** and **6e**) in all three cancer cell lines. The data are expressed as IC_50_ (mean ± SD) in a panel of four cancer cell lines (MCF-7, HTB-54, HT-29, and HCT-116), and are shown in Table 1. Cisplatin was used as a standard drug. All the selected selenoureas displayed stronger in vitro inhibitory activities against some of the cell lines compared to cisplatin. Additionally, reported IC_50_ values of a well-known antitumor selenocompound, i.e., sodium selenite (Na_2_SeO_3_), were used as reference for comparision with the selected selenoureas. The only IC_50_ reported value found in the literature for sodium selenite was 17 µM in MCF-7 cells after 72 h of treatment [42]. It is noteworthy that seven selenoureas (**1e**, **2a**, **6a**, **6b**, **6c**, **6d**, and **6e**) showed greater cytotoxic activity than sodium selenite towards this cancer cell line, even at shorter timepoints. The highest cytotoxic activity among the selenourea derivatives was exhibited by adamantyl compounds in all the cell lines with IC_50_ values below 10 µM for compounds **6b**, **6c**, **6d**, and **6e**. No correlation was observed with the insertion of electron-withdrawing or -donating groups.

The selectivity index (SI) was calculated as the ratio between IC_50_ values for the normal cell line and IC_50_ values for malignant cell lines. High SI values are desirable, since they reflect efficacy with less toxicity. Compounds with an IC_50_ below 10 µM in MCF-7 or in HTB-54 were assayed against two non-tumoral cell lines derived from breast (184B5) and lung (BEAS-2B), respectively. According to the SI results, most of the compounds exhibited SI values below 1, with the exception of **2a**, **6a**, **6b**, **6c**, **6d**, and **6e** in breast cancer, and only **6d** in lung cancer. Remarkably, the most effective analog (**6c**) presents similar or slightly better cytotoxic activity than many of the most recent urea [43] and thiourea [44] derivatives reported, showing much greater antitumor effects than several thiosemicarbazone derivatives recently published in relation to MCF-7 cells [45,46].

To further explore the potential of these selenoureas, the most effective analog, **6c**, against the four cell lines was selected for testing in the NCI-60 cell line panel. This compound shows an average cell growth inhibition of −9.58% at 10 µM, meaning that compound **6c** is able to kill cells in most of the cell lines. The dose–response was also studied in the same 60 cancer cell lines, with outstanding results. Average GI_50_ of 1.49 µM and TGI of 9.17 µM were found. Table 2 summarizes the GI_50_, TGI, and LC_50_ values of representative cell lines. Full data mean GI_50_ graphs are provided in the Supplementary Materials. The activity profile was found to be broad and especially pronounced for colon, central nervous system, melanoma, ovarian, renal, and breast cancers.

#### 3.3.2. Cell Cycle Modulation by Selenourea **6c**

The arrest of cell cycle progression caused by some anticancer drugs entails the inhibition of cancer cell proliferation [49,50]. Moreover, previous studies with selenium compounds have demonstrated that these compounds could induce arrest in cell cycle phases [51,52].

**Figure 5:**
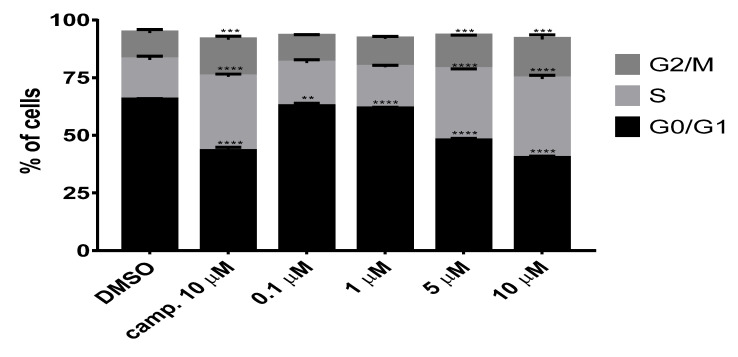
Cell cycle modulation of selenourea **6c** at different concentrations after 48 h of treatment compared with the vehicle (DMSO) and 10 µM of camptothecin (camp.) as positive control. Results are expressed as a mean ± SD of duplicates of three independent experiments, ** *p* ≤ 0.01, *** *p* ≤ 0.001 and **** *p*≤ 0.0001 compared to the vehicle (DMSO).

To gain further knowledge about the mechanism of action of **6c**, we investigated the effect of this compound on cell cycle progression using flow cytometry analysis, with propidium iodide as a DNA staining agent. We analyzed the effect at 48 h treatment with four different concentrations (0.1, 1, 5, and 10 µM) of compound **6c** on HT-29 cells. Camptothecin (10 μM) was used as a positive control. As shown in Figure 5, compound **6c** caused cell cycle arrest at the S phase in a concentration-dependent manner. The cell cycle modulation of **6c** is very similar to camptothecin, the reference compound used in the study.

#### 3.3.3. Cell Death Induced by **6c**

**Figure 6:**
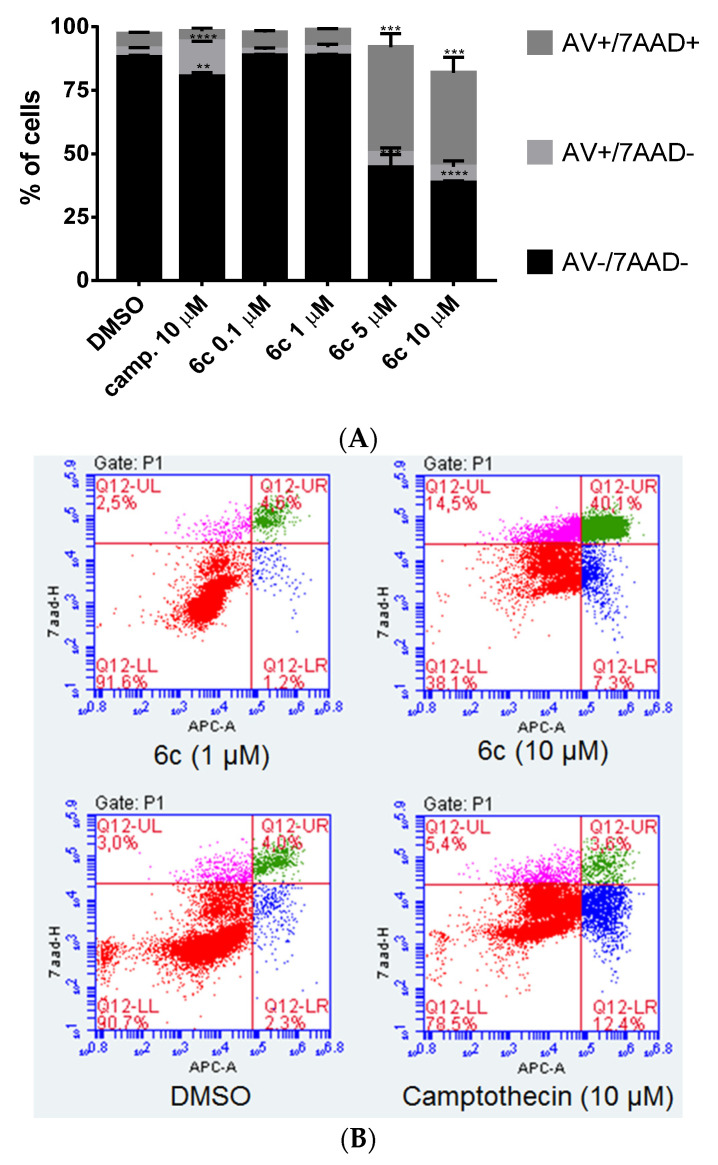

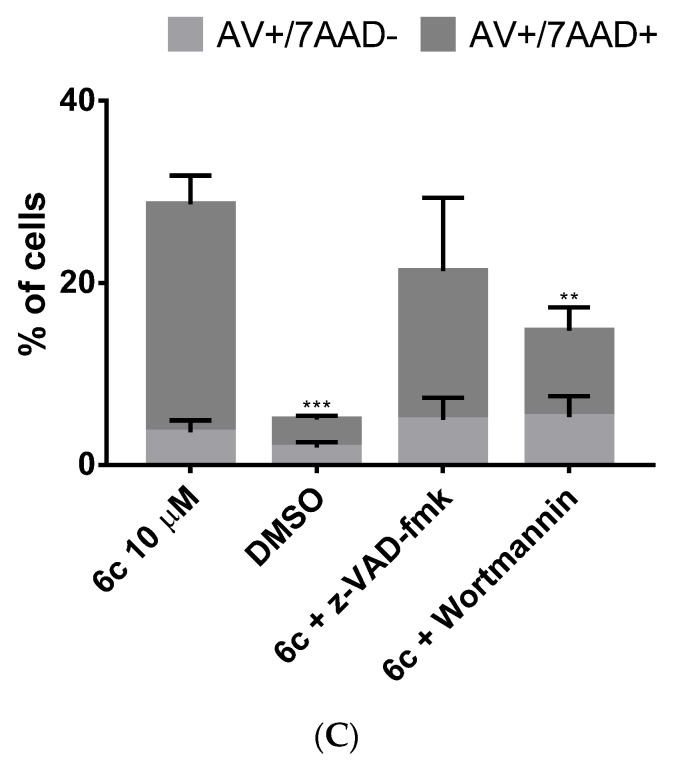
(**A**) Percentage of live cells (AV−/7AAD−), live cells with loss membrane integrity (AV+/7AAD−), and dead cells (AV+/7AAD+) after 48 h of treatment at different concentrations in HT-29 cells. Results are expressed as a mean ± SD of three independent experiments, ** *p* ≤ 0.01, *** *p* ≤ 0.001 and **** *p* ≤ 0.0001 compared to non-treated cells (DMSO). (**B**) Annexin V-APC/7AAD assay of HT-29 cells treated with 1 and 5 µM of **6c**, vehicle (DMSO), and when treated with 10 µM of camptothecin (Camp.) after 48 h of treatment. Cell population is represented in different colors: red (AV−/7AAD-), blue (AV+/7AAD−), green (AV+/7AAD+), pink (AV−/7AAD+). (**C**) Percentage of AV+/7AAD− and AV+/7AAD+ cells after 24 h of incubation with or without 30 min preincubation with z-VAD-fmk or wortmannin. Results are expressed as a mean ± SD of three independent experiments, ** *p* ≤ 0.01 and *** *p* ≤ 0.001 compared to treatment with **6c** at 10 µM.

Mounting evidence of the anticancer potential of selenium compounds can be found in the literature. Diverse mechanisms, including apoptosis, necroptosis, necrosis, and autophagy, have been shown to be involved in cell death induction by selenium compounds. Remarkably, these mechanisms vary depending on the selenium compound and on cell phenotype [53]. To further analyze the ability of the selenourea **6c** to induce cell death, we performed Annexin V-APC/7AAD assays in HT-29 cells cultured either in the presence or absence of this compound for 48 h. Assays were conducted at different **6c** concentrations ranging from 0.1 to 10 µM. Camptothecin (10 µM) was used as positive control. As shown in Figure 6A,B, 5 and 10 μM **6c** increased the number of dead cells (7AAD positive cells). Interestingly, no significant differences in the number of dead cells were found between HT-29 cells treated with **6c** and HT-29 cells treated with **6c** after pre-incubation with the pancaspase inhibitor z-VAD-fmk (Figure 6C). However, pre-incubation of the cells with the PI3K inhibitor wortmannin prevented induction cell death by **6c** (Figure 6C). These findings indicate that the induction of cell death by **6c** is independent of caspase activation, and suggest autophagy, though this assertion requires additional studies.

## 4. Conclusions

In the present work, 30 novel *N*,*N*-disubstituted selenoureas were synthesized and tested for their cytotoxic and antioxidant activities in vitro. The cytotoxicity of these selenoureas was assessed in different cancer cell lines of breast, lung, and colon cancer. Adamantyl-containing selenoureas **6a**–**e** were the most potent cytotoxic agents, whereas aliphatic selenoureas (**4a**–**e**, **5a**–**e**) were the least cytotoxic. No specific correlation in the cytotoxic activity between selenoureas containing different electron-withdrawing and electron-donating groups at the 4-phenyl position was found. An MTT assay demonstrated that five compounds (**1e**, **6b**, **6c**, **6d** and **6e**) exhibited IC_50_ values below 10 µM against the four cancer cells lines tested (breast, lung, and two colon), showing greater cytotoxic activity than the reference cisplatin. Adamantyl-containing selenourea **6c** was selected as the most potent cytotoxic agent, and was tested in the NCI60 cell line panel, with outstanding results. Average GI_50_ of 1.49 µM and TGI of 9.17 µM were found, showing more potent antiproliferative activity than several anticancer drugs, including 5-fluorouracil, gefitinib, or oxaliplatin. To gain insight into its anticancer activity mechanisms, we investigated the cell cycle progression of this promising **6c** compound, as well as the type of programmed-cell death in a colon cancer cell line it causes (HT-29). Compound **6c** provoked S phase cell cycle arrest with a decrease in cells in the G1 phase, and the induction of cell death was independent of caspase activation, suggesting autophagy, though this assertion requires additional studies. Overall, we envision that this compound can be further developed for the potential treatment of colon cancer.

The radical scavenging ability of the new selenoureas was evaluated using DPPH and ABTS assays. All these selenoureas present important antioxidant activity in DPPH and ABTS assays, presenting, in some cases, higher or similar radical scavenging activity than the reference substances (ascorbic acid and Trolox). Nitrile para-substituted selenoureas **1**–**6e** and coumarin-derived selenoureas **3a**–**e** present fast kinetics, reaching the maximal antioxidant activity at time 0 in a DPPH assay. On average and in both experiments, thiazol-containing selenourea **2c** was found to be the most potent antioxidant compound at low concentrations.

Although no overall correlation between cytotoxicity and antioxidant activity was found, compound **6c** demonstrated an outstanding cytotoxic activity, along with potent radical scavenging activity. These results provide an excellent starting point for the development of new selenium-based medication with both antioxidant and chemotherapeutic potential.

## Figures and Tables

**Table 1:** Cytotoxic activity expressed as average IC_50_ values (µM) and selectivity indexes (SI) calculated for selected selenoureas. Cisplatin was taken as reference compound.

Compound	IC_50_ (µM) *							
	Breast			Lung			Colon	
	MCF-7	184B5	SI **	HTB-54	BEAS-2B	SI	HT-29	HCT-116
**1d**	>10	N.D.	N.D.	>10	N.D.	N.D.	8.07 ± 3.77	7.80 ± 0.56
**1e**	9.96 ± 1.45	8.09 ± 1.21	0.81	9.79 ± 0.07	6.01 ± 0.94	0.61	5.89 ± 1.32	9.14 ± 1.14
**2a**	9.16 ± 3.49	11.00 ± 0.73	1.20	>10	N.D.	N.D.	>10	N.D.
**2d**	>10	N.D.	N.D.	8.65 ± 0.29	5.22 ± 0.07	0.60	>10	N.D.
**3a**	>10	N.D.	N.D.	>10	N.D.	N.D.	6.49 ± 1.58	5.86 ± 0.52
**6a**	9.88 ± 1.46	22.24 ± 4.07	2.25	>10	N.D.	N.D.	5.03 ± 3.06	5.69 ± 0.71
**6b**	5.52 ± 1.61	12.24 ± 2.42	2.21	9.01 ± 2.60	7.56 ± 0.19	0.83	5.67 ± 4.67	6.29 ± 0.76
**6c**	4.94 ± 1.99	7.23 ± 2.28	1.46	5.09 ± 0.28	4.73 ± 0.79	0.93	5.60 ± 1.24	3.60 ± 0.74
**6d**	7.19 ± 1.12	15.96 ± 2.74	2.22	6.56 ± 0.99	7.59 ± 0.53	1.16	8.04 ± 2.17	6.95 ± 0.65
**6e**	5.47 ± 3.91	7.69 ± 0.31	1.41	6.09 ± 0.75	2.36 ± 0.39	0.39	4.18 ± 0.46	5.43 ± 0.86
**Cisplatin**	15.16 ± 1.04 [47]	N.D.	N.D.	13.68 ± 0.83 [48]	N.D.	N.D.	14.18 ± 0.73 [47]	2.18 ± 1.58

* IC_50_: half maximal inhibitory concentration ** SI: selectivity index was calculated as the ratio of the IC_50_ values determined for the non-malignant and the tumoral cells (IC_50_ (184B5)/IC_50_ (MCF-7) or (IC_50_ (BEAS-2B)/IC_50_ (HTB-54) N.D.: not determine.

**Table 2:** Most remarkable GI_50_, TGI, and LC_50_ values for selenourea **6c** in different cancer cell lines. Results were obtained from the NCI-60 program. Full information for the 60 cancer cell lines is presented in the Supplementary Materials.

Cance Type	Cell Line	GI_50_ (µM) *	TGI (µM) **	LC_50_ (µM) ***
Colon cancer	COLO-205	1.66	3.98	9.33
Colon cancer	HCT-116	1.29	2.75	6.03
CNS cancer	SF-593	1.91	4.17	9.33
Melanoma	M14	1.38	3.31	7.94
Melanoma	SK-MEL-5	0.56	1.78	4.27
Ovarian cancer	OVACAR-3	1.38	3.24	7.41
Renal cancer	UO-31	1.74	4.07	9.77
Breast cancer	MDA-MB-231/ATCC	1.78	3.89	8.51
Breast cancer	BT-549	0.87	2.29	5.62
Breast cancer	MDA-MB-468	1.35	3.39	8.71

* GI_50_: concentration for 50% inhibition of cell proliferation ** TGI: concentration for total grow inhibition *** LC_50_: Lethal dose at which 50% of cell population is killed.

## Data Availability

The data presented in this study are available on reasonable request from the corresponding author.

## Supplementary Materials

The detailed synthesis of *N*,*N′*-disubstituted selenoureas; NCI-60 dose–response report for **6c** (Figures S1–S5); spectroscopic characterization for all compounds; NMR spectra (Figures S6–S80) and DPPH (Figures S81–S100) and ABTS (Figures S101–S106) additional results are available online at https://www.mdpi.com/article/10.3390/antiox10050777/s1.
