# Supplementary material for: Novel N,N′-Disubstituted Selenoureas as Potential Antioxidant and Cytotoxic Agents
Source: Antioxidants (Basel). 2021 May 14;10(5):777. doi: 10.3390/antiox10050777 (PMC8156206; doi:10.3390/antiox10050777)
Supplement: Supplementary file 1 [file antioxidants-10-00777-s001.zip › antioxidants-1200696-supplementary.pdf]

Novel *N,N′*-Disubstituted selenoureas as Potential Antioxidant and Cytotoxic Agents

Gorka Calvo-Martin [1,2,†], Daniel Plano [1,2,†], Ignacio Encío [2,3], Carmen Sanmartín [1,2,#]

[1] Departamento de Tecnología y Química Farmacéuticas, Universidad de Navarra, Irunlarrea 1, E-31008 Pamplona, Spain

[2] Instituto de Investigación Sanitaria de Navarra (IdiSNA), Irunlarrea, 3, 31008 Pamplona, Spain

[3] Departamento de Ciencias de la Salud, Universidad Pública de Navarra, Avda. Barañain s/n, 31008, Pamplona, Spain

[#] Correspondence: sanmartin@unav.es; Tel.: +34-948425600 ext. 806388

* These authors have contributed equally to the manuscript

Contents:

1. Synthesis of *N,N´*-disubstituted selenoureas.
2. NCI-60 dose-response report for **6c (Figures S1-S4).**
3. General structure used to assign the chemical shifts in NMR spectroscopy (**Figure S5**) and characterization of the compounds synthetized in this work.
4. NMR spectra (^{1}H, ^{13}C and ^{77}Se) of final products (**Figures S6-S80**)
5. Supplementary data for DPPH and ABTS assays (**Figures S81-S106**).

1. SYNTHESIS OF *N,N´*-DISUBSTITUTED SELENOUREAS

1.1. Synthesis of the compounds **1a-e**

0.75 mmol (1,5 eq.) of furan-2-ymethylamine were added over a solution of 0.5 mmol of the selected isoselenocyanate in 5 mL of DCM., The mixture was stirred for one hour. After one hour the solvent was evaporated under vacuum and the resulted oil was precipitated with n-hexane. The precipitate was filtered and washed with *n*-hexane.

1.2. Synthesis of the compounds **2a-e**

0.84 mmol of thiazol-2-ylmethylamine (1.5 equivalents) were added over a solution of 1 equivalent of the corresponding arylisoselenocyanate dissolved in 10 mL of DCM. The mixture was stirred for 1 hour at RT. After this time the precipitate was filtered and washed with *n*-hexane.

1.3. Synthesis of the compounds **3a-e.**

0.6 mmol (1.1 eq) of 6-aminocoumarin were added over a solution of 1 equivalent of the corresponding arylisoselenocyanate in 10 mL of THF and the mixture was stirred for four hours. After this time the solvent was evaporated in a rotary evaporator. The resulted oil was precipitated with a 1:1 mixture of DCM/n-Hexane and washed with the same proportion to obtain the selenoureas **4a-e** as yellow powder.

1.4. Synthesis of the compounds **4a-e**

1.5 mmol of butylamine (1.5 eq) were added to a solution of 1 mmol of the corresponding arylisoselenocyanate dissolved in 10 mL of DCM and the mixture was stirred at room temperature for 20 min. After 20 min the solvent was evaporated under reduced pressure and the resulted oil precipitated with *n*-hexane. the precipitate was filtered and washed with *n*-hexane to obtain the products as white powder.

1.5. Synthesis of the compounds **5a-e.**

1.5 mmol of hexylamine (1.5 eq) were added to a solution of 1 mmol of the corresponding arylisoselenocyanate dissolved in 10 mL of DCM and the mixture was stirred at room temperature for 45 min. After 45 min the solvent was evaporated under reduced pressure and the resulted oil precipitated with *n*-hexane. the precipitate was filtered and washed with *n*-hexane to obtain the product as white-pink powder.

1.6. Synthesis of the compounds **6a-e.**

0.7 mmol adamantylamine were added to a solution of 0.7 mmol of the corresponding arylisoselenocyanate in 10 mL of DCM and the mixture was stirred at room temperature for 30 min. The solvent was evaporated under reduced pressure and the resulted oil was precipitated with *n*-hexane. The precipitate was filtered and washed with n-hexane to obtain the products as white powder.

2. NCI-60 DOSE-RESPONSE REPORT FOR 6c

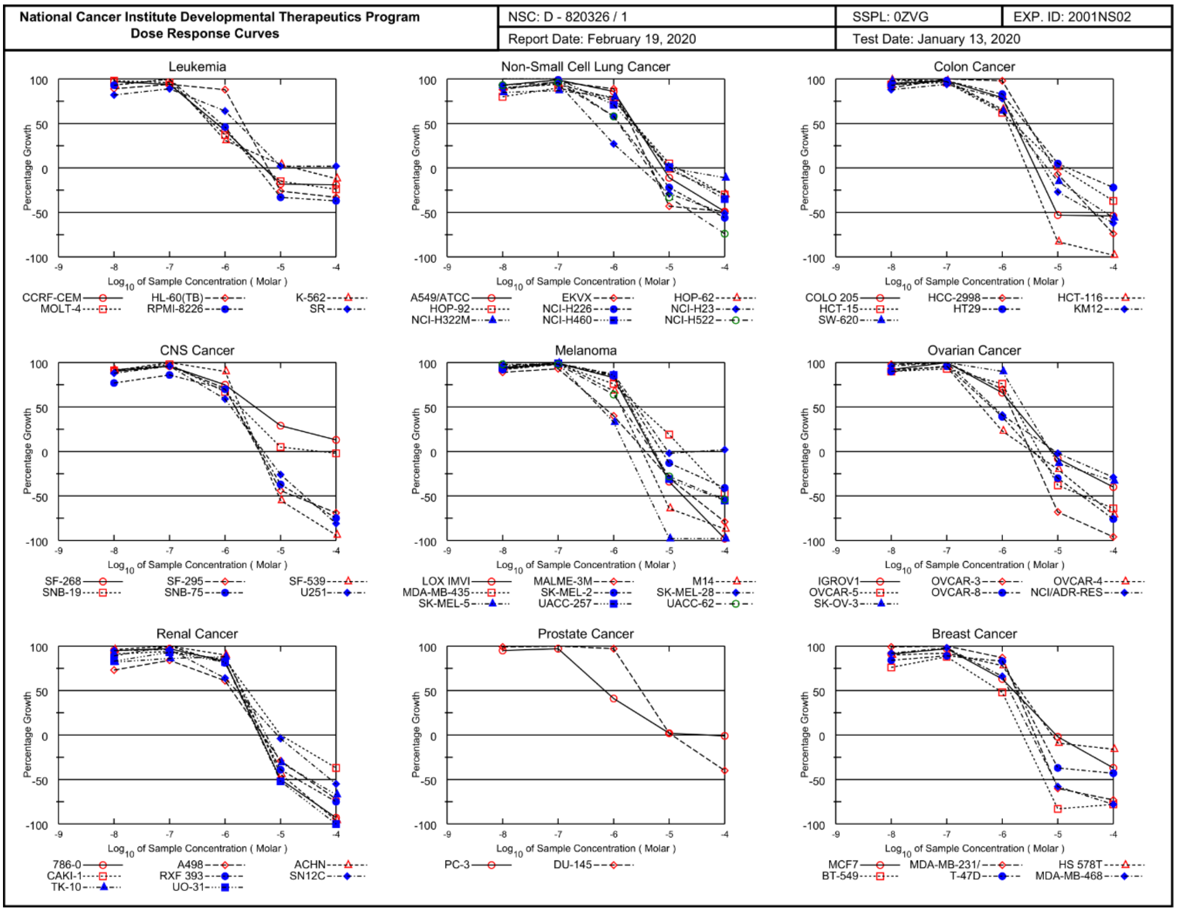

Figure S1. NCI-60 dose-response report for **6c**. Page 1 of 4.

National Cancer Institute Developmental Therapeutics Program
In-Vitro Testing Results

NSC : D - 820326 / 1	Experiment ID : 2001NS02	Test Type : 08	Units : Molar
Report Date : February 19, 2020	Test Date : January 13, 2020	QNS :	MC :
COMI : SU-8c	Stain Reagent : SRB Dual-Pass Related	SSPL : 0ZVG	

			Log10 Concentration												
	Time		Mean Optical Densities					Percent Growth							
Panel/Cell Line	Zero	Ctrl	-8.0	-7.0	-6.0	-5.0	-4.0	-8.0	-7.0	-6.0	-5.0	-4.0	GI50	TGI	LC50
Leukemia															
CCRF-CEM	0.531	2.039	1.997	1.945	1.167	0.434	0.432	97	94	42	-18	-19	7.05E-7	4.97E-6	> 1.00E-4
HL-60(TB)	0.745	2.912	2.663	2.792	2.653	0.551	0.503	89	94	88	-26	-33	2.15E-6	5.91E-6	> 1.00E-4
K-562	0.169	1.583	1.487	1.608	0.611	0.232	0.149	93	102	31	4	-12	5.42E-7	1.88E-5	> 1.00E-4
MOLT-4	0.633	2.859	2.813	2.781	1.465	0.536	0.483	98	96	37	-15	-24	6.11E-7	5.12E-6	> 1.00E-4
RPMI-8226	1.012	2.229	2.145	2.284	1.568	0.677	0.635	93	105	46	-33	-37	8.45E-7	3.80E-6	> 1.00E-4
SR	0.291	1.632	1.393	1.482	1.145	0.318	0.315	82	89	64	2	2	1.67E-6	> 1.00E-4	> 1.00E-4
Non-Small Cell Lung Cancer															
A549/ATCC	0.483	2.628	2.488	2.603	2.337	0.432	0.249	93	99	86	-11	-49	2.38E-6	7.78E-6	> 1.00E-4
EKVX	1.070	2.124	2.001	2.088	2.006	0.614	0.545	88	97	89	-43	-49	1.97E-6	4.74E-6	> 1.00E-4
HOP-62	1.222	3.004	2.830	2.898	2.613	1.200	0.853	90	94	78	-2	-30	2.24E-6	9.49E-6	> 1.00E-4
HOP-92	1.344	1.984	1.859	1.919	1.831	1.379	0.939	80	90	76	5	-30	2.34E-6	1.42E-5	> 1.00E-4
NCI-H226	1.051	1.917	1.854	1.908	1.551	0.817	0.459	93	99	58	-22	-56	1.25E-6	5.26E-6	6.50E-5
NCI-H23	0.905	2.457	2.309	2.376	1.322	0.639	0.446	90	95	27	-29	-51	4.56E-7	3.00E-6	9.20E-5
NCI-H322M	0.998	2.348	2.140	2.178	2.080	0.989	0.888	85	87	80	.	-11	2.35E-6	9.73E-6	> 1.00E-4
NCI-H460	0.238	2.727	2.729	2.716	1.993	0.266	0.155	100	100	71	1	-35	1.98E-6	1.07E-5	> 1.00E-4
NCI-H522	1.289	3.194	3.052	3.116	2.396	0.866	0.332	93	96	58	-33	-74	1.23E-6	4.35E-6	2.59E-5
Colon Cancer															
COLO 205	0.661	2.904	2.798	2.862	2.439	0.308	0.306	95	98	79	-53	-54	1.66E-6	3.95E-6	9.41E-6
HCC-2998	0.510	2.002	1.853	2.000	1.975	0.472	0.132	90	100	98	-7	-74	2.86E-6	8.50E-6	4.34E-5
HCT-116	0.288	2.286	2.276	2.249	1.607	0.050	0.005	99	98	66	-83	-98	1.28E-6	2.78E-6	6.02E-6
HCT-15	0.299	2.249	2.133	2.176	1.504	0.333	0.187	94	96	62	2	-37	1.57E-6	1.11E-5	> 1.00E-4
HT29	0.431	2.765	2.593	2.727	2.369	0.542	0.335	93	98	83	5	-22	2.64E-6	1.50E-5	> 1.00E-4
KM12	0.387	2.145	1.939	2.036	1.758	0.283	0.149	88	94	78	-27	-62	1.85E-6	5.54E-6	4.63E-5
SW-620	0.414	2.600	2.558	2.536	1.815	0.352	0.181	98	97	64	-15	-56	1.51E-6	6.45E-6	7.04E-5
CNS Cancer															
SF-268	1.138	2.887	2.714	2.810	2.457	1.650	1.372	90	96	75	29	13	3.55E-6	> 1.00E-4	> 1.00E-4
SF-295	0.873	3.136	2.964	3.043	2.473	0.492	0.273	92	96	71	-44	-69	1.52E-6	4.15E-6	1.79E-5
SF-539	0.767	2.363	2.218	2.360	2.203	0.347	0.046	91	100	90	-55	-94	1.89E-6	4.18E-6	9.27E-6
SNB-19	0.550	2.025	1.886	1.997	1.534	0.624	0.539	91	98	67	5	-2	1.86E-6	5.06E-5	> 1.00E-4
SNB-75	0.925	1.836	1.629	1.707	1.563	0.582	0.235	77	86	70	-37	-75	1.54E-6	4.50E-6	2.20E-5
U251	0.498	2.526	2.289	2.440	1.693	0.367	0.094	88	96	59	-26	-81	1.27E-6	4.91E-6	2.71E-5
Melanoma															
LOX IMVI	0.442	2.929	2.756	2.901	2.506	0.290	0.008	93	99	83	-34	-98	1.91E-6	5.09E-6	1.76E-5
MALME-3M	0.724	1.798	1.679	1.723	1.156	0.507	0.155	89	93	40	-30	-79	6.53E-7	3.74E-6	2.58E-5
M14	0.411	1.112	1.068	1.130	0.889	0.149	0.055	94	103	68	-64	-87	1.37E-6	3.29E-6	7.87E-6
MDA-MB-435	0.640	2.928	2.807	2.883	2.368	1.079	0.341	95	98	76	19	-47	2.84E-6	1.95E-5	> 1.00E-4
SK-MEL-2	1.185	2.793	2.662	2.765	2.554	1.028	0.700	92	98	85	-13	-41	2.27E-6	7.33E-6	> 1.00E-4
SK-MEL-28	0.964	2.673	2.610	2.657	2.454	0.948	0.993	96	99	87	-2	2	2.62E-6	.	> 1.00E-4
SK-MEL-5	1.104	3.173	3.108	3.163	1.791	0.022	0.025	97	99	33	-98	-98	5.58E-7	1.79E-6	4.30E-6
UACC-257	1.261	2.841	2.750	2.831	2.620	0.871	0.563	94	99	86	-31	-55	2.03E-6	5.44E-6	6.03E-5
UACC-62	0.982	2.891	2.861	2.862	2.210	0.706	0.456	98	98	64	-28	-54	1.43E-6	4.96E-6	7.24E-5
Ovarian Cancer															
IGROV1	0.568	2.312	2.173	2.325	1.717	0.520	0.342	92	101	66	-8	-40	1.64E-6	7.70E-6	> 1.00E-4
OVCAR-3	0.585	1.920	1.894	1.943	1.516	0.189	0.024	98	102	70	-68	-96	1.39E-6	3.21E-6	7.42E-6
OVCAR-4	0.681	1.750	1.661	1.694	0.927	0.548	0.195	92	95	23	-20	-71	4.20E-7	3.46E-6	3.87E-5
OVCAR-5	0.534	1.401	1.318	1.337	1.197	0.333	0.194	90	93	76	-38	-64	1.70E-6	4.68E-6	2.97E-5
OVCAR-8	0.413	2.133	1.961	2.059	1.089	0.291	0.100	90	96	39	-30	-76	6.45E-7	3.71E-6	2.76E-5
NCI/ADR-RES	0.583	2.214	2.156	2.235	1.253	0.570	0.413	96	101	41	-2	-29	7.11E-7	8.88E-6	> 1.00E-4
SK-OV-3	1.100	2.585	2.583	2.611	2.440	0.953	0.736	100	102	90	-13	-33	2.45E-6	7.43E-6	> 1.00E-4
Renal Cancer															
786-0	0.762	2.636	2.544	2.572	2.323	0.381	0.053	95	97	83	-50	-93	1.78E-6	4.22E-6	1.00E-5
A498	1.775	2.641	2.406	2.504	2.303	1.259	0.496	73	84	61	-29	-72	1.32E-6	4.75E-6	3.07E-5
ACHN	0.340	1.501	1.456	1.536	1.383	0.192	0.015	96	103	90	-44	-96	1.99E-6	4.71E-6	1.32E-5
CAKI-1	0.741	2.518	2.364	2.413	2.243	0.747	0.464	91	94	85	.	-37	2.57E-6	1.02E-5	> 1.00E-4
RXF 393	1.312	2.008	1.930	2.025	1.874	0.800	0.334	89	102	81	-39	-75	1.80E-6	4.72E-6	2.03E-5
SN12C	0.644	2.550	2.454	2.477	1.862	0.620	0.290	95	96	64	-4	-55	1.61E-6	8.81E-6	8.00E-5
TK-10	0.784	1.910	1.712	1.752	1.777	0.544	0.261	82	86	88	-31	-67	2.10E-6	5.52E-6	3.44E-5
UO-31	0.902	2.031	1.841	1.955	1.828	0.437	0.003	83	93	82	-52	-100	1.74E-6	4.11E-6	9.74E-6
Prostate Cancer															
PC-3	0.542	2.056	1.978	2.014	1.166	0.565	0.534	95	97	41	2	-1	6.96E-7	3.21E-5	> 1.00E-4
DU-145	0.762	2.517	2.493	2.572	2.464	0.799	0.458	99	103	97	2	-40	3.13E-6	1.12E-5	> 1.00E-4
Breast Cancer															
MCF7	0.288	1.785	1.657	1.737	1.225	0.283	0.182	91	97	63	-2	-37	1.57E-6	9.40E-6	> 1.00E-4
MDA-MB-231/ATCC	0.730	1.657	1.650	1.719	1.538	0.292	0.200	99	107	87	-60	-73	1.79E-6	3.91E-6	8.55E-6
HS 578T	1.059	2.292	2.168	2.194	2.015	0.965	0.893	90	92	78	-9	-16	2.08E-6	7.89E-6	> 1.00E-4
BT-549	0.955	2.092	1.821	1.951	1.497	0.163	0.212	76	88	48	-83	-78	8.74E-7	2.32E-6	5.60E-6
T-47D	1.039	2.473	2.244	2.318	2.229	0.650	0.597	84	89	83	-37	-43	1.88E-6	4.89E-6	> 1.00E-4
MDA-MB-468	0.864	1.838	1.759	1.818	1.509	0.366	0.187	92	98	66	-58	-78	1.35E-6	3.42E-6	8.68E-6

Figure S2. NCI-60 dose-response report for **6c**. Page 2 of 4.

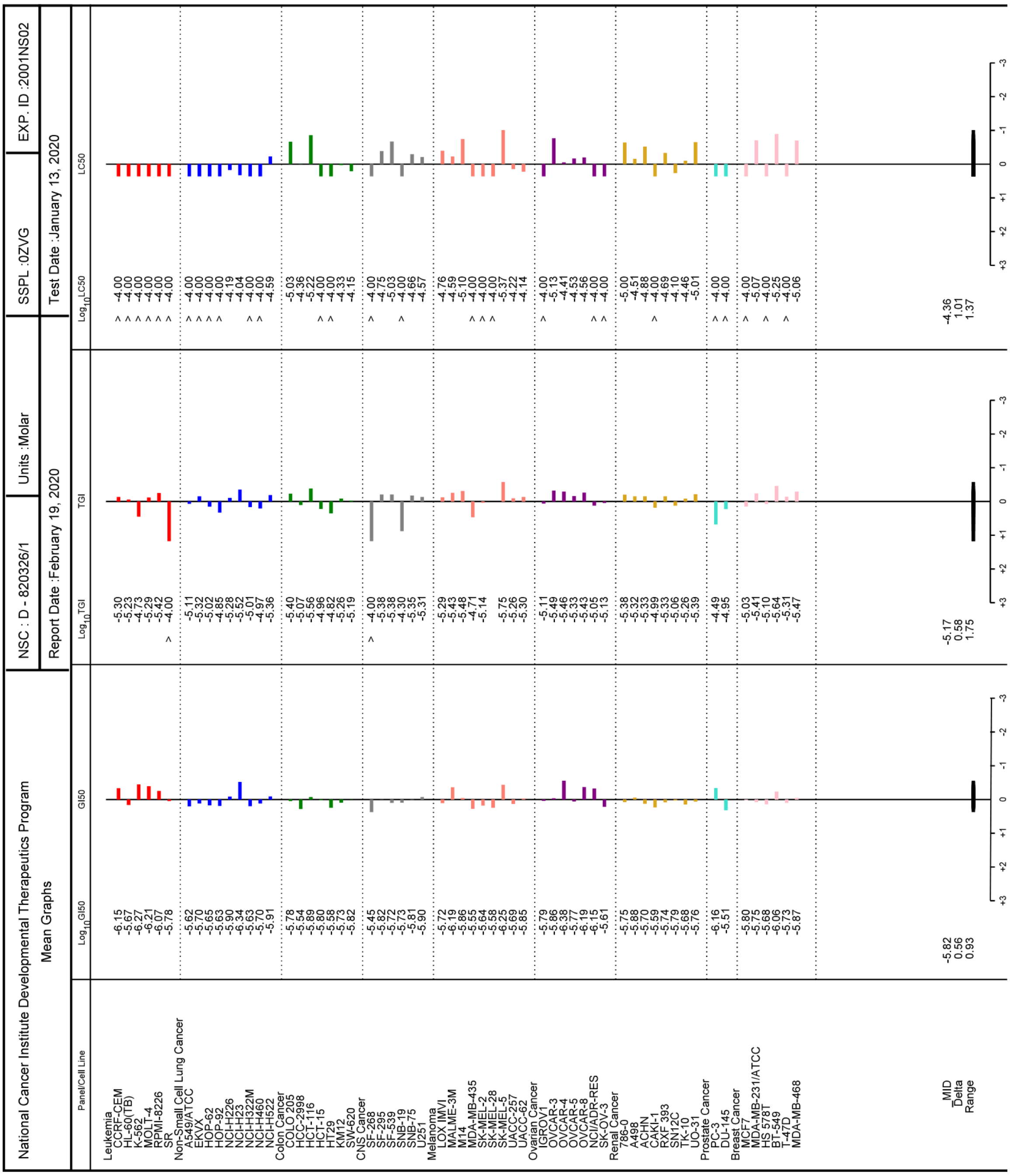

Figure S3. NCI-60 dose-response report for **6c**. Page 3 of 4.

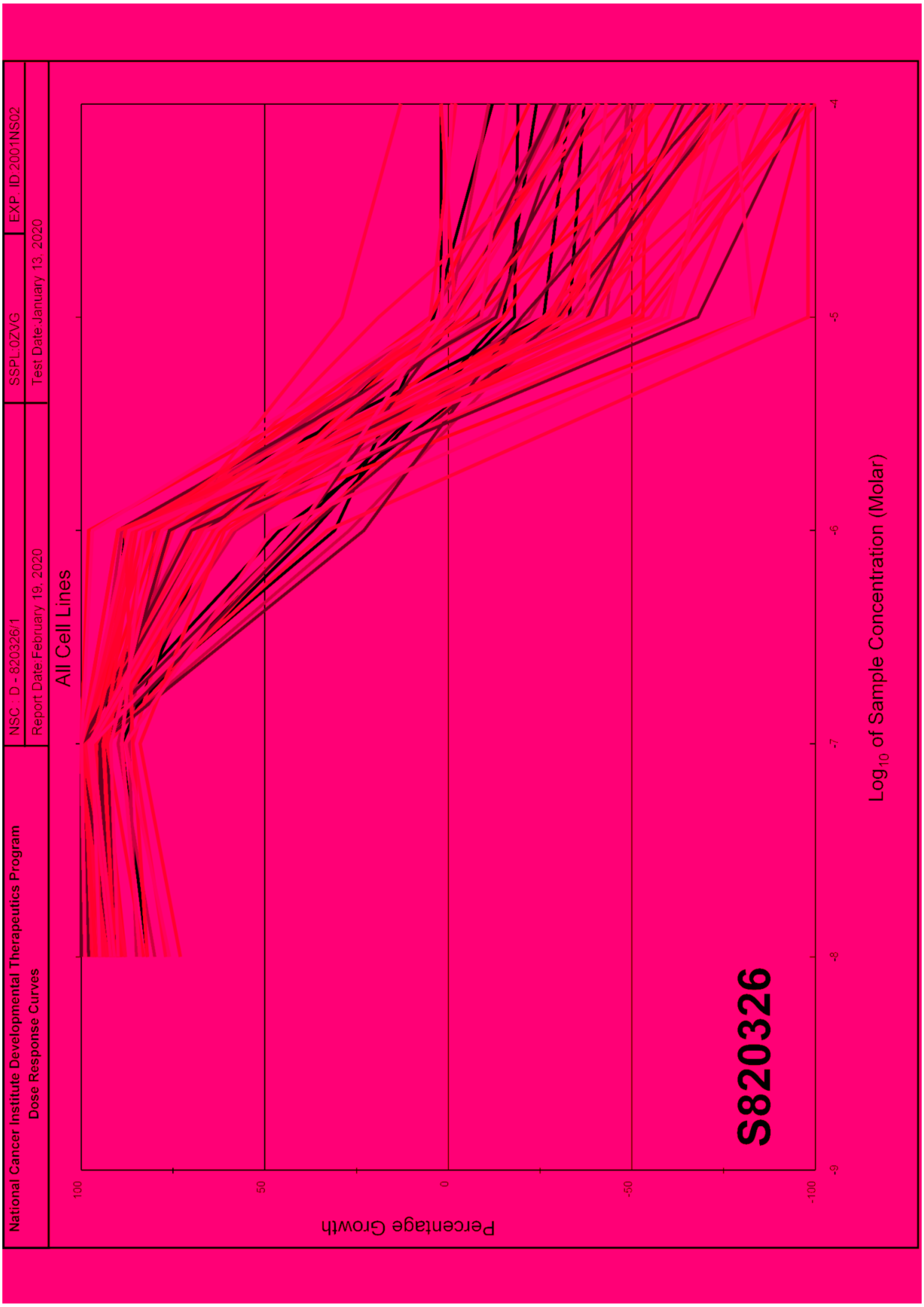

Figure S4. NCI-60 dose-response report for **6c**. Page 4 of 4.

3. GENERAL STRUCTURE USED TO ASSIGN THE CHEMICAL SHIFTS IN NMR SPECTROSCOPY

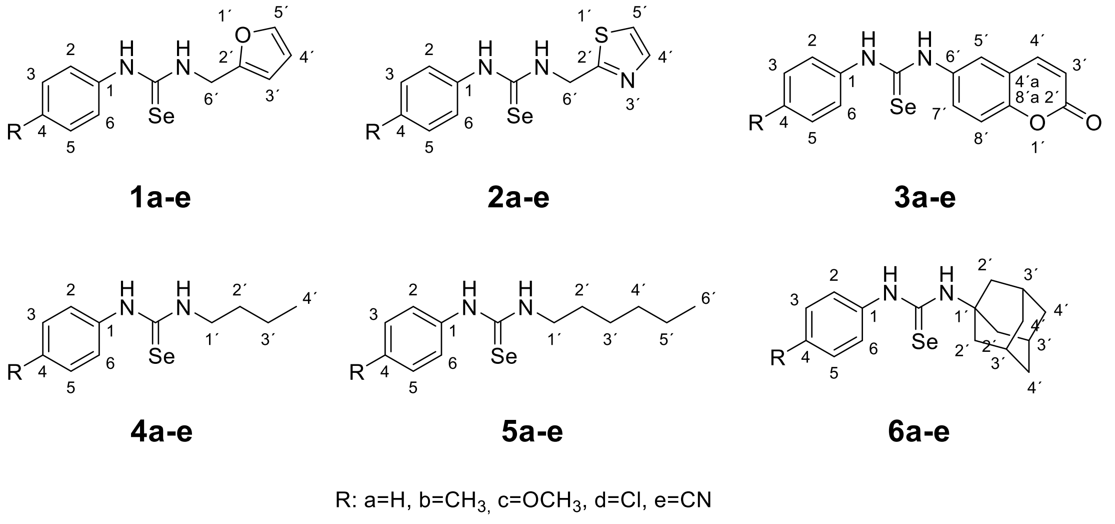

Figure S5. General structure of the new *N,N´*-disubstituted selenoureas.

N´-(furan-2-ylmethyl)-N-phenylselenourea (**1a**). Yield 80%, mp 109 ºC. IR (KBr) ʋ cm-1: 3262 (N-H, 3141 (N-H), 2993 (Csp^3-H). ^{1}H NMR (400 MHz, $CDCl_3$) δ: 8.34 (br s, 1H, NH), 7.44 (dd, 2H, $J_{3\text{-}4} = J_{5\text{-}4}$ =7.7 Hz; $J_{3\text{-}2}$ =$J_{5\text{-}6}$ =7.7 Hz; H_3+H_5), 7.36 – 7.30 (m, 2H, H_4+$H_{5'}$), 7.21 (d, 2H, H_2+H_6), 6.51 (br s, 1H, N´H), 6.31 (d, 2H, $J_{3'\text{-}5'}$= $J_{4'\text{-}5'}$= 1.3 Hz, $H_{3'}$+$H_{4'}$), 4.93 (d, 2H, $J_{6'}$-N´H = 5.1 Hz, $H_{6'}$). ^{13}C NMR (100 MHz, $CDCl_3$) δ: 179.3 (C=Se), 149.8 ($C_{2'}$), 142.7 ($C_{5'}$), 135.7 (C_1), 130.53 (C_3+C_5), 128.0 (C_4), 125.3 (C_2+C_6), 110.64 ($C_{4'}$), 108.59 ($C_{3'}$), 45.35 ($C_{6'}$). 77Se NMR (76 MHz, $CDCl_3$) δ: 222.2 (Se=C). HRMS calcd. For $C_{12}H_{12}N_2OSe$ [M+H]: 281.0188, found 281.0191 [M+H].

N´-(furan-2-ylmethyl)-N-(4-methyl)phenylselenourea (**1b**). Yield 60%, 157 ºC. IR (KBr) ʋ cm^{-1}: 3282 (N-H), 3155 (N-H), 3085 (C_{sp2}-H), 2936 (C_{sp3}-H). ^{1}H NMR (400 MHz, $CDCl_3$) δ: δ 8.17 (br s, 1H, NH), 7.33 (s, 1H, $H_{5'}$), 7.23 (d, 2H, $J_{3\text{-}2}$= $J_{5\text{-}6}$= 8.1 Hz; H_3+H_5), 7.09 (d, 2H, H_2+H_6), 6.43 (br s, 1H, N´H), 6.31 (s, 2H, $H_{3'}$+$H_{4'}$), 4.92 (d, 2H, $J_{6'\text{-}N'H}$ = 5.1 Hz, $H_{6'}$), 2.35 (s, 3H, CH_3-Ph). ^{13}C NMR (100 MHz, $CDCl_3$) δ: 179.1 (C=Se), 150.0 ($C_{2'}$), 142.6 ($C_{5'}$), 138.3 (C_1), 132.9 (C_4), 131.1 (C_3+C_5), 125.5 (C_2+C_6), 110.6 ($C_{4'}$), 108.5 ($C_{3'}$), 45.5 ($C_{6'}$), 21.2 (CH_3-Ph). HRMS calcd. For $C_{13}H_{14}N_2OSe$ [M+H]: 295.0344, found 295.0349 [M+H].

N´-(furan-2-ylmethyl)-N-(4-methoxy)phenylselenourea (**1c**). Yield 86%, mp 128 ºC. IR (KBr) ʋ cm^{-1}: 3322 (N-H), 3171 (N-H), 2963 (C_{sp3}-H). ^{1}H NMR (400 MHz, $CDCl_3$) δ: 8.30 (s, 1H, NH), 7.32 (dd, 1H, $J_{5'\text{-}3'}$= 1.7 Hz, $J_{5'\text{-}4'}$=0.9 Hz, $H_{5'}$), 7.14 (d, 2H, $J_{2\text{-}3}$=$J_{6\text{-}5}$ = 8.8 Hz, H_2+H_6), 6.92 (d, 2H, H_3+H_5), 6.41 – 6.24 (m, 3H, NH+$H_{3'}$+$H_{4'}$), 4.90 (d, 2H, $J_{6'\text{-}N'H}$= 5.2 Hz, $H_{6'}$), 3.80 (s, 3H, OCH_3). ^{13}C NMR (100 MHz, $CDCl_3$) δ: 179.7 (C=Se), 159.4 (C_4), 150.0 ($C_{2'}$), 142.6 ($C_{5'}$), 128.0 (C_1), 127.6 (C_2+C_6), 115.6 (C_3+C_5), 110.6 ($C_{4'}$), 108.5 ($C_{3'}$), 55.7 (OCH_3), 45.3 ($C_{1'}$).^{77}Se NMR (76 MHz, $CDCl_3$) δ: 206.7 (Se=C). HRMS calcd. For $C_{13}H_{14}N_2O_2Se$ [M+H]: 311.0293, found 311.0284 [M+H].

N´-(furan-2-ylmethyl)-N-(4-chloro)phenylselenourea (**1d**). Yield 79%, mp 128 ºC. IR (KBr) ʋ cm^{-1}: 3357 (N-H), 3152 (N-H), 3033 (C_{sp2}-H). ^{1}H NMR (400 MHz, $CDCl_3$) δ: 8.78 (s, 1H, NH), 7.38 (d, 2H, $J_{3\text{-}2}$=$J_{5\text{-}6}$= 8.7 Hz, H_3+H_5), 7.35 – 7.31 (m, 1H, $H_{5'}$), 7.17 (d, 2H, H_2+H_6), 6.47 (br s, 1H, N´H), 6.31 (d, 2H, $H_{3'}$+$H_{4'}$), 4.90 (d, 2H, $J_{6'\text{-}N'}$= 4.6 Hz, $H_{6'}$). ^{13}C NMR (100 MHz, $CDCl_3$) δ 179.4 (C=Se), 149.6 ($C_{2'}$), 142.8 ($C_{5'}$), 134.3 (C_1), 133.6 (C_4), 130.6 (C_3+C_5), 126.7 (C_2+C_6), 110.7 ($C_{4'}$), 108.8 ($C_{3'}$), 45.3 ($C_{6'}$). ^{77}Se NMR (76 MHz, $CDCl_3$) δ: 229.3 (Se=C). HRMS calcd. For $C_{12}H_{11}N_2OSeCl$ [M+H]: 314.9798, found 314.9789 [M+H].

N´-(furan-2-ylmethyl)-N-(4-cyano)phenylselenourea (**1e**). Yield 79%, mp 128ºC. IR (KBr) ῡ cm^{-1}: 3353 (N-H), 3166 (N-H), 3052 (C_{sp2}-H), 2982 (C_{sp3}-H), 2226 (C≡N). ^{1}H NMR (400 MHz, DMSO) δ: 10.33 (br s, 1H, NH), 9.03 (br s, 1H, N´H), 7.78 (d, 2H, $J_{3\text{-}2}$=$J_{5\text{-}6}$= 8.8 Hz, H_3+H_5), 7.68 (d, 2H, H_2+H_6), 7.65 - 760 (m, 1H, $H_{5'}$), 6.43 (dd, 1H, $J_{4'\text{-}3'}$ = 3.1 Hz, $J_{4'\text{-}5'}$= 1.9 Hz, $H_{4'}$), 6.39 (d, 1H, $H_{3'}$), 4.84 (d, 2H, $J_{6'\text{-}NH}$= 1.4 Hz, $H_{6'}$). ^{13}C NMR (100 MHz, DMSO) δ 180.3 (C=Se), 150.7 ($C_{2'}$), 143.7 (C_1), 142.4 ($C_{5'}$), 132.9 (C_3+C_5), 122.7 (C_2+C_6), 118.9 (C_4), 110.5 ($C_{4'}$), 107.9 ($C_{3'}$), 105.9 (CN), 43.5 ($C_{6'}$). ^{77}Se NMR (76 MHz, $CDCl_3$) δ: 251.53 (Se=C). HRMS calcd. For $C_{13}H_{11}N_3OSe$ [M+H]: 306.0140, found 306.0153 [M+H].

N´-(thiazol-2-ylmethyl)-N-phenylselenourea (**2a**). Yield 65%, mp 149ºC. IR (KBr) ῡ cm^{-1}: 3131 (N-H), 3048 (C_{sp2}-H), 2969 (C_{sp3}-H). ^{1}H NMR (400 MHz, DMSO) δ: 10.30 (br s, 1H, NH), 8.74 (br s, 1H, N´H), 7.74 (d, 1H, $J_{4'\text{-}5'}$= 3.2 Hz, $H_{4'}$), 7.63 (d, 1H, $H_{5'}$), 7.39 (t, 2H, J= 7.7 Hz, H_3+H_5), 7.33 (d, 2H, $J_{2\text{-}3}$=$J_{6\text{-}5}$=7.3 Hz, H_2+H_6), 7.23 (t, 1H, $J_{4\text{-}3}$=$J_{4\text{-}5}$= 7.2 Hz, H_4), 5.14 (d, 2H $J_{6'\text{-}N'}$= 4.0 Hz, $H_{6'}$). ^{13}C NMR (101 MHz, DMSO) δ: 178.0 (C=Se), 168.6 ($C_{2'}$), 142.0 ($C_{4'}$), 138.2 (C_1), 129.14 (C_3+C_5), 125.7 (C_4), 124.6 (C_2+C_6), 119.9 ($C_{5'}$), 48.5 ($C_{6'}$). ^{77}Se NMR (76 MHz, DMSO) δ: 219.4 (C=Se). HRMS calcd. For $C_{11}H_{11}N_3SSe$ [M+H]: 297.9912, found 297.9915 [M+H].

N´-(thiazol-2-ylmethyl)-N-(4-methyl)phenylselenourea (**2b**). Yield 58%, mp 154 ºC. IR (KBr) ῡ cm^{-1}: 3253 (N-H), 3205 (N-H), 2976 (C_{sp3}-H). ^{1}H NMR (400 MHz, DMSO) δ: 10.20 (br s, 1H, NH), 8.61 (br s, 1H, NH), 7.73 (d, 1H, $J_{4'\text{-}5'}$ = 3.2 Hz; $H_{4'}$), 7.62 (d, 1H, $H_{5'}$), 7.19 (s, 4H, H_2+H_3+H_5+H_6), 5.12 (d, 2H $J_{6'\text{-}N'H}$ = 4.5 Hz; 2H), 2.29 (s, 3H, CH_3-Ph). ^{13}C NMR (101 MHz, DMSO) δ: 179.8 (C=Se), 168.8 ($C_{2'}$), 142.0 ($C_{4'}$), 135.4 (C_1), 135.2 (C_4), 129.6 (C_3+C_5), 124.8 (C_2+C_6), 119.8 ($C_{5'}$), 48.5 ($C_{6'}$), 20.5 (CH_3-Ph). ^{77}Se NMR (76 MHz, DMSO) δ: 213.4 (C=Se). HRMS calcd. For $C_{12}H_{13}N_3SSe$ [M+H]: 312.0068, found 312.0099 [M+H].

N´-(thiazol-2-ylmethyl)-N-(4-methoxy)phenylselenourea (**2c**). Yield 62%, mp 150 ºC. IR (KBr) ῡ cm^{-1}: 3191 (N-H), 2957 (C_{sp3}-H). ^{1}H NMR (400 MHz, DMSO) δ: 10.11 (br s, 1H, NH), 8.51 (br s, 1H, N´H), 7.72 (d, 1H, $J_{4'\text{-}5'}$ = 3.3 Hz, $H_{4'}$), 7.61 (d, 1H, $H_{5'}$), 7.19 (d, 2H, $J_{3\text{-}2}$=$J_{5\text{-}6}$= 8.9 Hz, H_3+H_5), 6.96 (d, 2H, H_2+H_6), 5.10 (d, 2H, $J_{6'\text{-}N'H}$ = 5.1 Hz, H6´), 3.75 (s, 3H, OCH3). ^{13}C NMR (101 MHz, DMSO) δ 1780.0 (C=Se), 169.0 ($C_{2'}$), 157.5 (C_1), 142.0 ($C_{4'}$), 130.6 (C_4), 126.9 (C_3+C_5), 119.8 ($C_{5'}$), 114.4 (C_2+C_6), 55.3 (OCH_3), 48.5 ($C_{6'}$). HRMS calcd. For $C_{12}H_{13}N_3OSSe$ [M+H]: 328.0017, found 328.0082 [M+H].

N´-(thiazol-2-ylmethyl)-N-(4-chloro)phenylselenourea (**2d**). Yield 76%, mp 165 ºC. IR (KBr) ῡ cm^{-1}: 3266 (N-H), 3191 (N-H), 3089 (C_{sp2}-H), 2975 (C_{sp3}-H). ^{1}H NMR (400 MHz, DMSO) δ: 10.31 (br s, 1H, NH), 8.85 (br s, 1H, NH), 7.74 (d, 1H, $J_{4'\text{-}5'}$ = 3.2 Hz; $H_{4'}$), 7.63 (d, 1H, $H_{5'}$), 7.44 (d, 2H, $J_{3\text{-}2}$ = $J_{5\text{-}6}$ = 8.7 Hz; H_3+H_5), 7.37 (d, 2H, H_2+H_6), 5.13 (s, 2H, $H_{6'}$). ^{13}C NMR (101 MHz, DMSO) δ: 180.5 (C=Se), 168.3 ($C_{2'}$), 142.0 ($C_{4'}$), 137.4 (C_1), 129.7 (C_4), 128.9 (C_3+C_5), 126.3 (C_2+C_6), 120.0 ($C_{5'}$), 48.4 ($C_{6'}$). ^{77}Se NMR (76 MHz, DMSO) δ: 224.0 (C=Se). HRMS calcd. For $C_{11}H_{10}N_3SSeCl$ [M+H]: 331.9522, found 331.9517 [M+H].

N´-(thiazol-2-ylmethyl)-N-(4-cyano)phenylselenourea (**2e**). Yield 68%, mp 152ºC. IR (KBr) ῡ cm^{-1}: 3199 (N-H), 2924 (C_{sp3}-H), 2225 (C≡N). ^{1}H NMR (400 MHz, DMSO) δ: 10.44 (br s, 1H, NH), 9.30 (br s, 1H, NH), 7.81 (d, 2H, $J_{3\text{-}2}$ = $J_{5\text{-}6}$ = 8.5 Hz; H_3+H_5), 7.76 (d, 1H, $J_{4'\text{-}5'}$ = 3.0 Hz; $H_{4'}$), 7.68 (d, 2H, H_2+H_6), 7.65 (d, 1H, $H_{5'}$)., 5.17 (s, 2H, $H_{6'}$). ^{13}C NMR (100 MHz, DMSO) δ: 181.4 (C=Se), 167.9 ($C_{2'}$), 143.8 (C_1), 142.5 ($C_{4'}$), 133.6 (C_3+C_5), 123.6 (C_2+C_6), 120.7 ($C_{5'}$), 119.4 (C_4), 106.8 (CN), 48.7 ($C_{6'}$). ^{77}Se NMR (76 MHz, DMSO) δ: 255.5 (C=Se). HRMS calcd. For $C_{12}H_{10}N_4SSe$ [M+H]: 322.9864, found 322.9867 [M+H].

N´-(6-Coumarin)-N-Phenylselenourea (**3a**). Yield 78 %, mp 169ºC. IR (KBr) ῡ cm^{-1}: 3273 (N-H), 3153 (N-H), 1729 (C=O). ^{1}H NMR (400 MHz, DMSO) δ: 10.27 (br s, 1H, NH), 10.21 (br s, 1H, NH), 8.07 (d, 1H, $J_{H4'\text{-}H3'}$ = 9.6 Hz; $H_{4'}$), 7.73 (d, 1H, $J_{H7'\text{-}H8'}$ = 2.3 Hz; $H_{7'}$), 7.60 (dd, 1H, $J_{H8'\text{-}H5'}$ = 8.8 Hz; $J_{H8'\text{-}H7'}$= 2.3 Hz; $H_{8'}$), 7.44 – 7.32 (m, 5H, H_2+H_3+H_5+H_6+$H_{5'}$), 7.19 (t, 1H, $J_{H4\text{-}H3,H5}$ = 7.2 Hz, H_4), 6.50 (d, 1H, $H_{3'}$).^{13}C

NMR (100 MHz, DMSO) δ: 179.7 (C=Se), 160.4 ($C_{2'}$), 151.5 ($C_{8'a}$), 144.5 ($C_{4'}$), 139.9 (C1), 136.7 ($C_{6'}$), 130.2 ($C_{8'}$), 129.1 (C_3+C_5), 125.9 (C_4), 125.3 (C_2+C_6), 125.2 ($C_{7'}$), 119.0 ($C_{4'a}$), 116.8 ($C_{3'}$+$C_{5'}$). HRMS calcd. For $C_{16}H_{12}N_2O_2Se$ [M+H]: 345.0137, found 345.0138 [M+H].

N´-(-6-Coumarin)-N-(4-methylphenyl)selenourea (**3b**). Yield 86 %, mp 145 ºC. IR (KBr) ῡ cm^{-1}: 3278 (N-H), 3100 (N-H), 1720 (C=O). ^1H NMR (400 MHz, DMSO) δ: 10.17 (br s, 1H, NH), 10.07 (br s, 1H, N´H), 8.06 (d, 1H, $J_{H4'-H3'}$ = 9.6 Hz; $H_{4'}$), 7.72 (d, 1H, $J_{H7'-H8'}$ = 2.3 Hz; $H_{7'}$), 7.59 (dd, 1H, $J_{H8'-H5'}$ = 8.8 Hz; $J_{H8'-H7'}$= 2.4 Hz; $H_{8'}$), 7.37 (d, 1H, $H_{5'}$), 7.27 (d, 2H, J_{H3-H2} = J_{H5-H6} = 8.2 Hz; H_3+H_5), 7.16 (d, 2H, H_2+H_6), 6.49 (d, 1H, $H_{3'}$), 2.29 (s, 3H, CH_3-Ph). ^{13}C NMR (100 MHz, DMSO) δ: 179.6 (C=Se), 160.4 ($C_{2'}$), 151.5 ($C_{8'a}$), 144.6 ($C_{4'}$), 137.2 (C_1), 136.8 ($C_{6'}$), 135.3 (C_4), 130.3 ($C_{8'}$), 129.6 (C_3+C_5), 125.4 (C_2+C_6), 125.3 ($C_{7'}$), 119.0 ($C_{4'a}$), 116.7 ($C_{3'}$+$C_{5'}$), 21.0 (CH_3-Ph). HRMS calcd. For $C_{17}H_{14}N_2O_2Se$ [M+H]: 359.0293, found 359.0293 [M+H].

N´-(-6-Coumarin)-N-(4-methoxyphenyl)selenourea (**3c**). Yield 62 %, mp 159 ºC. IR (KBr) ῡ cm^{-1}: 3264 (N-H), 3118 (N-H), 1717 (C=O). ^1H NMR (400 MHz, DMSO) δ: 10.09 (br s, 1H, NH), 9.99 (br s, 1H, N´H), 8.06 (d, 1H, $J_{H4'-H3'}$ = 9.6 Hz; $H_{4'}$), 7.71 (s, 1H, $H_{7'}$), 7.59 (d, 1H, $J_{H8'-H5'}$ = 8.8 Hz, $H_{8'}$), 7.37 (d, 1H, $H_{5'}$), 7.27 (d, 2H, JH_{3-H2} = J_{H5-H6} = 8.5 Hz; H_3+H_5), 6.92 (d, 2H, H_2+H_6), 6.49 (d, 1H, $H_{3'}$), 3.75 (s, 3H, OCH_3). ^{13}C NMR (100 MHz, DMSO) δ: 179.68 (C=Se), 160.4 ($C_{2'}$), 157.7 (C_4), 151.5 ($C_{8'a}$), 144.6 ($C_{4'}$), 136.7 ($C_{6'}$), 132.6 (C_1), 130.3 ($C_{8'}$), 127.4 (C_3+C_5), 125.3 ($C_{7'}$), 119.0 ($C_{4'a}$), 116.7 ($C_{3'}$+$C_{5'}$), 114.4 (C_2+C_6), 55.7 (OCH_3). HRMS calcd. For $C_{17}H_{14}N_2O_3Se$ [M+H]: 375.0242, found 375.0238 [M+H].

N´-(-6-Coumarin)-N-(4-chlorophenyl)selenourea (**3d**). Yield 96 %, mp 156 ºC. IR (KBr) ῡ cm^{-1}: 3280 (N-H), 3109 (N-H), 1716 (C=O). ^1H NMR (400 MHz, DMSO) δ: 10.30 (br s, 1H, NH), 10.26 (br s, 1H, N´H), 8.07 (d, 1H, $J_{H4'-H3'}$ = 9.3 Hz; $H_{4'}$), 7.73 (s, 1H, $H_{7'}$), 7.60 (d, 1H, $J_{H8'-H5'}$ = 7.8 Hz, $H_{8'}$), 7.52-7.35 (m, 5H, H_2+H_3+H_5+H_6+$H_{5'}$), 6.50 (d, 1H, $H_{3'}$). ^{13}C NMR (100 MHz, DMSO) δ 180.0 (C=Se), 160.4 ($C_{2'}$), 151.6 ($C_{8'a}$), 144.5 ($C_{4'}$), 139.0 (C_1), 136.4 ($C_{6'}$), 130.1 ($C_{8'}$), 129.9 (C_4), 129.0 (C_3+C_5), 127.1 (C_2+C_6), 125.1 ($C_{7'}$), 119.1 ($C_{4'a}$), 116.9 ($C_{3'}$), 116.8 ($C_{5'}$). HRMS calcd. For $C_{16}H_{11}N_2O_2SeCl$ [M+H]: 378.9747, found 378.9763 [M+H].

N´-(-6-Coumarin)-N-(4-cyanophenyl)selenourea (**3e**). Yield 72 %, mp 140 ºC. IR (KBr) ῡ cm^{-1}: 3269 (N-H), 2223 (C≡N), 1727 (C=O). ^1H NMR (400 MHz, DMSO) δ: 10.62 (s, 2H, NH+N´H), 8.08 (d, 1H, $J_{H4'-H3'}$ = 9.6 Hz; $H_{4'}$), 7.80 (d, 2H, JH_{3-H2} = J_{H5-H6} = 8.6 Hz; H_3+H_5), 7.75 (d, 1H, $J_{H7'-H8'}$ = 2.2 Hz; $H_{7'}$), 7.69 (d, 2H, H_2+H_6), 7. (dd, 1H, $J_{H8'-H5'}$ = 8.8 Hz; $J_{H8'-H7'}$= 2.3 Hz; $H_{8'}$), 7.41 (d, 1H, $H_{5'}$), 6.51 (d,1H, $H_{3'}$). ^{13}C NMR (101 MHz, DMSO) δ: 179.8 (C=Se), 159.8 ($C_{2'}$), 151.2 ($C_{8'a}$), 144.1 (C_1), 144.0 ($C_{4'}$), 135.8 ($C_{6'}$), 132.7 (C_3+C_5), 129.3 ($C_{8'}$), 124.4 ($C_{7'}$), 123.9 (C_2+C_6), 118.9 ($C_{4'a}$), 118.7 (C_4), 116.6 ($C_{5'}$), 116.4 ($C_{3'}$), 106.4 (CN). HRMS calcd. For $C_{17}H_{11}N_3O_2Se$ [M+H]: 370.0089, found 370.0103 [M+H].

N-butyl-N´-phenylselenourea (**4a**). Yield 82%, mp 69ºC. IR (KBr) ῡ cm^{-1}: 3356 (N-H), 3123 (N-H), 2962 (C_{sp3}-H). ^1H NMR (400 MHz, $CDCl_3$) δ: 8.33 (br s, 1H, NH), 7.44 (dd, 2H, J_{3-4} = J_{5-4} =7.7 Hz; J_{3-2} =J_{5-6} =7.7 Hz; H_3+H_5), 7.33 (t, 1H, H_4), 7.20 (d, 2H, H_2+ H_6), 6.23 (br s, 1H, NH), 3.69 (dt, 2H, $J_{H1'-NH}$ =6.3; $J_{H1'-H2'}$ =6.5 Hz, $H_{1'}$), 1.55 (quint, 2H, J=7.4 Hz; $H_{2'}$), 1.38 – 1.25 (m, 2H, $H_{3'}$), 0.91 (t, 3H, $J_{H4'-JH3'}$ = 7.3 Hz; $H_{4'}$). ^{13}C NMR (100 MHz, $CDCl_3$) δ: 179.0 (C=Se), 135.8, 130.5, 127.9, 125.4 (aryl), 48.2 ($C_{1'}$), 31.1 ($C_{2'}$), 20.1 ($C_{3'}$), 13.8 ($C_{4'}$).

N-butyl-N´-(4-methyl)selenourea (**4b**). Yield 81%, mp 46ºC. IR (KBr) ῡ cm^{-1}: 3256 (N-H), 3194 (N-H), 2961(C_{sp3}-H). ^1H NMR (400 MHz, $CDCl_3$) δ: 8.16 (br s, 1H, NH), 7.23 (d, 2H, J_{3-2} = J_{5-6} = 8.1 Hz, H_3+H_5), 7.08 (d, 2H, H_2+H_6), 6.15 (br s, 1H, NH), 3.68 (dt, $J_{H1'-NH}$ =6.3; $J_{H1'-H2'}$ =6.5 Hz, $H_{1'}$), 2.35 (s, 3H, CH_3-Ph), 1.54 (quint, 2H, J=7.4 Hz; $H_{2'}$), 1.37 – 1.25 (m, 2H, $H_{3'}$), 0.90 (t, 3H, $J_{H4'-JH3'}$ = 7.3 Hz, H4′). ^{13}C NMR (100 MHz, $CDCl_3$) δ: 178.8 (C=Se), 138.1, 133.0, 131.0, 125.6 (Aryl), 48.2 ($C_{1'}$), 31.2 ($C_{2'}$), 21.2 (CH_3-Ph), 20.1 ($C_{3'}$), 13.86 ($C_{4'}$).

N-butyl-N´-(4-methoxyphenyl)selenourea (**4c**). Yield 87%, mp 89ºC. IR (KBr) ῡ cm^{-1}: 3319 (N-H), 3203 (N-H), 3053 (C_{sp2}-H), 2955 (C_{sp3}-H). ^{1}H NMR (400 MHz, $CDCl_3$) δ: 8.07 (br s, 1H, NH), 7.13 (d, 2H, $J_{3-2} = J_{5-6}$ = 8.8 Hz, H_3+H_5), 6.94 (d, H_2+H_6), 6.01 (br s, 1H, NH), 3.81 (s, 3H, OCH_3), 3.67 (dt, 2H, $J_{H1'-NH}$ =6.3; $J_{H1'-H2'}$ =6.5 Hz, $H_{1'}$), 1.53 (quint, 2H, J=7.4; $H_{2'}$), 1.36 – 1.22 (m, 2H, $H_{3'}$), 0.90 (t, 3H, $J_{H4'-H3'}$ = 7.3 Hz, $H_{4'}$). ^{13}C NMR (100 MHz, $CDCl_3$) δ: 179.2 (C=Se), 159.4, 128.1, 127.8, 115.6 (Aryl), 55.7 (OCH_3), 48.1 ($C_{1'}$), 31.2 ($C_{2'}$), 20.1 ($C_{3'}$), 13.9 ($C_{4'}$).

N-butyl-N´-(4-chlorophenyl)selenourea (**4d**). Yield 60%, mp 116ºC. IR (KBr) ῡ cm^{-1}: 3229 (N-H), 3127 (N-H), 3051 (C_{sp2}-H), 2954 (C_{sp3}-H). ^{1}H NMR (400 MHz, $CDCl_3$) δ: 8.47 (br s, 1H, NH), 7.41 (d, 2H, $J_{3-2} = J_{5-6}$ = 8.6 Hz, H_3+H_5), 7.16 (d, 2H, H_2+H_6), 6.15 (br s, 1H, NH), 3.69 (d, 2H, $J_{H1'-H2'}$ = 5.3 Hz, $H_{1'}$), 1.55 (quint, 2H, J=7.4; $H_{2'}$), 1.47 – 1.14 (m, 2H, $H_{3'}$), 0.92 (t, 3H, $J_{H4'-H3'}$ = 7.3 Hz, $H_{4'}$). ^{13}C NMR (100 MHz, $CDCl_3$) δ: 179.0 (C=Se), 134.3, 133.6, 130.7, 126.8 (Aryl), 48.2 ($C_{1'}$), 31.1 ($C_{2'}$), 20.1 ($C_{3'}$), 13.9 (C4´). HRMS calcd. For $C_{11}H_{15}N_2SeCl$ [M+H]: 291.0162, found 291.0158 [M+H].

N-butyl-N´-(4-cyanophenyl)selenourea (**4e**). Yield 70%, mp 145 ºC. IR (KBr) ῡ cm^{-1}: 3316 (N-H), 3136 (N-H), 2953 (C_{sp3}-H), 2229 (C≡N). ^{1}H NMR (400 MHz, $CDCl_3$) δ 8.52 (br s, 1H, NH), 7.72 (d, 2H, $J_{3-2} = J_{5-6}$ = 8.3 Hz, H_3+H_5), 7.33 (br s, 2H, H_2+H_6), 6.46 (br s, 1H, NH), 3.73 (br s, 2H, $H_{1'}$), 1.63 (quint, 2H, J=7.4; $H_{2'}$), 1.38 (m, 2H, $H_{3'}$), 0.95 (t, 3H, $J_{H4'-H3'}$ = 7.3 Hz, $H_{4'}$). ^{13}C NMR (101 MHz, DMSO) δ 179.3 (C=Se), 143.7 (C_1), 133.1 (C_3+C_5), 122.3 (C_2+C_6), 119.0 (C_4), 105.4 (CN), 46.5 ($C_{1'}$), 30.2 ($C_{2'}$), 19.6 ($C_{3'}$), 13.7 ($C_{4'}$). ^{77}Se NMR (76 MHz, DMSO) δ: 249.4 (C=Se). HRMS calcd. For $C_{11}H_{15}N_3Se$ [M+H]: 282.0504, found 282.0507 [M+H].

N-hexyl-N´-phenylselenourea (**5a**). Yield 80%, mp 59ºC. IR (KBr) ῡ cm^{-1}: 3203 (N-H), 3047 (C_{sp2}-H), 2956 (C_{sp3}-H). ^{1}H NMR (400 MHz, $CDCl_3$) δ: 8.24 (br s, 1H, NH), 7.44 (dd, 2H, $J_{3-4} = J_{5-4}$ =7.7 Hz; J_{3-2} =J_{5-6} =7.7 Hz; H3+H5), 7.33 (t, 1H, H_4), 7.21 (d, 2H, H_2+ H_6), 6.23 (br s, 1H, NH), 3.68 (dt, 2H, $J_{H1'-NH}$ =6.5; $J_{H1'-H2'}$ =6.5 Hz; $H_{1'}$), 1.59-1.54 (m, 2H, $H_{2'}$), 1.32 – 1.22 (m, 6H, $H_{3'}$+$H_{4'}$+$H_{5'}$), 0.86 (t, 3H, $J_{H6'-H5'}$ = 6.8 Hz; $H_{6'}$). ^{13}C NMR (100 MHz, $CDCl_3$) δ: 178.8 (C=Se), 135.8, 130.5, 128.0, 125.5 (Aryl), 48.5 ($C_{1'}$), 31.47 ($C_{2'}$), 29.0 ($C_{3'}$), 26.6 ($C_{4'}$), 22.6 ($C_{5'}$), 14.0 ($C_{6'}$).

N-hexyl-N´-(4-metylphenyl)selenourea (**5b**). Yield 85%, mp 88 ºC. IR (KBr) ῡ cm^{-1}: 3232 (N-H), 3058 (C_{sp2}-H), 2925 (C_{sp3}-H). ^{1}H NMR (400 MHz, $CDCl_3$) δ: 8.13 (br s, 1H, NH), 7.23 (d, 2H, $J_{3-2} = J_{5-6}$ = 8.1 Hz; H_3+H_5), 7.08 (d, 2H, H_2+H_6), 6.15 (br s, 1H), 3.67 (dt, 2H, $J_{H1'-NH}$ =6.5; $J_{H1'-H2'}$ =6.5 Hz; $H_{1'}$), 2.36 (s, 3H, CH_3-Ph), 1.58-1.50 (m, 2H, $H_{2'}$), 1.26 (br s, 6H,), 0.86 (t, 3H, $J_{H6'-H5'}$ = 6.8 Hz; $H_{6'}$). ´). ^{13}C NMR (100 MHz, $CDCl_3$) δ: 178.8 (C=Se), 138.1, 132.9, 131.0, 125.5 (aryl), 48.4 ($C_{1'}$), 31.4 ($C_{2'}$), 28.9 ($C_{3'}$), 26.4 ($C_{4'}$), 22.5 ($C_{5'}$), 21.1 (CH_3-Ph), 13.9 ($C_{6'}$).

N-hexyl-N´-(4-methoxyphenyl)selenourea (**5c**). Yield 88%, mp 97ºC. IR (KBr) ῡ cm^{-1}: 3202 (N-H), 3054 (C_{sp2}-H), 2918 (C_{sp3}-H). ^{1}H NMR (400 MHz, $CDCl_3$) δ: 8.14 (br s, 1H), 7.13 (d, 2H, $J_{3-2} = J_{5-6}$ = 8.8 Hz; H_3+H_5), 6.93 (d, 2H, H_2+H_6), 6.02 (br s, 1H, NH), 3.81 (s, 3H, OCH_3), 3.65 (dt, 2H, $J_{H1'-NH}$ =6.5; $J_{H1'-H2'}$ =6.7 Hz; $H_{1'}$), 1.60 – 1.49 (m, 2H, $H_{2'}$), 1.25 (br s, 6H, $H_{3'}$+$H_{4'}$+$H_{5'}$), 0.85 (t, $J_{H6'-H5'}$ = 6.8 Hz; $H_{6'}$). ^{13}C NMR (100 MHz, $CDCl_3$) δ: 179.3 (C=Se), 159.4 (C_4), 128.3 (C_1), 127.8 (C_2+C_6), 115.6 (C_3+C_5), 55.7 (OCH_3), 48.4 ($C_{1'}$), 31.5 ($C_{2'}$), 29.1 ($C_{3'}$), 26.5 ($C_{4'}$), 22.6 ($C_{5'}$), 14.0 ($C_{6'}$). ^{77}Se NMR (76 MHz, $CDCl_3$) δ: 195.6 (C=Se). HRMS calcd. For $C_{14}H_{22}N_2OSe$ [M+H]: 315.0970, found 315.0966 [M+H].

N-hexyl-N´-(4-chlorophenyl)selenourea (**5d**). Yield 60%, mp 110 ºC. IR (KBr) ῡ cm^{-1}: 3226 (N-H), 3050 (C_{sp2}-H), 2926 (C_{sp3}-H). ^{1}H NMR (400 MHz, $CDCl_3$) δ: 8.47 (br s, 1H, NH), 7.40 (d, 2H, $J_{3-2} = J_{5-6}$ = 8.6 Hz; H_3+H_5), 7.16 (d, 2H, H_2+H_6), 6.15 (br s, 1H, NH), 3.67 (d, 2H $J_{H1'-NH}$ = 5.2 Hz, $H_{1'}$), 1.62 – 1.51 (m, 2H, $H_{2'}$), 1.27 (br s, 6H, $H_{3'}$+$H_{4'}$+$H_{5'}$), 0.86 (t, $J_{H6'-H5'}$ = 6.8 Hz; $H_{6'}$). ^{13}C NMR (100 MHz, $CDCl_3$) δ: 178.9 (C=Se), 134.2 133.5, 130.6, 126.7 (Aryl), 48.4 ($C_{1'}$), 31.3 ($C_{2'}$), 28.9 ($C_{3'}$), 26.5 ($C_{4'}$), 22.5 ($C_{5'}$), 13.9 ($C_{6'}$). HRMS calcd. For $C_{13}H_{19}N_2SeCl$ [M+H]: 319.0475, found 319.0470 [M+H].

N-hexyl-N´-(4-cyanophenyl)selenourea (**5e**). Yield 70%, mp 125ºC. IR (KBr) ῡ cm^{-1}: 3312 (N-H), 3137 (N-H), 2927 (C_{sp3}-H). ^{1}H NMR (400 MHz, $CDCl_3$) δ: 8.95 (br s, 1H, NH), 7.69 (d, 2H, $J_{3\text{-}2}$ = $J_{5\text{-}6}$ = 8.6 Hz; H_3+H_5), 7.36 (d, 2H, H_2+H_6), 6.55 (br s, 1H, NH), 3.70 (s, 2H, $H_{1'}$), 1.70 – 1.54 (m, 2H, $H_{2'}$), 1.31 (s, 6H, $H_{3'}$+$H_{4'}$+$H_{5'}$), 0.88 (t, $J_{H6'\text{-}H5'}$ = 6.7 Hz; $H_{6'}$). ^{13}C NMR (100 MHz, $CDCl_3$) δ: 179.0 (C=Se), 140.3, 134.3, 123.9, 118.0 (Aryl), 109.9 (C≡N), 48.6 ($C_{1'}$), 31.3 ($C_{2'}$), 28.7 ($C_{3'}$), 26.5 ($C_{4'}$), 22.5 ($C_{5'}$), 13.9 ($C_{6'}$). HRMS calcd. For $C_{14}H_{19}N_3Se$ [M+H]: 310.0817, found 310.0837 [M+H].

N´-adamantyl-N-(phenyl)selenourea (**6a**).Yield 87%, mp 161ºC. IR (KBr) ῡ cm^{-1}: 3355 (N-H), 3152 (C_{sp2}-H), 2900 (C_{sp3}-H). ^{1}H NMR (400 MHz, DMSO) δ: 9.60 (br s, 1H, NH), 7.49 (br s, 1H, N´H), 7.40 – 7.29 (m, 4H, H_2+H_3+H_5+H_6), 7.18 – 7.11 (m, 1H, H4), 2.28 (s, 6H, $H_{2'}$), 2.06 (s, 3H, $H_{3'}$), 1.64 (s, 6H, $H_{4'}$). ^{13}C NMR (100 MHz, DMSO) δ: 175.8 (C=Se), 139.2 (C_1), 128.6 (C_2+C_6), 125.0 (C_3+C_5), 124.7 (C_4), 54.4 (C'_1), 40.8 (C'_2), 35.8 (C'_4), 29.0 (C'_3). 77Se NMR (76 MHz, CDCl3) δ: 275.4 (C=Se). HRMS calcd. For $C_{17}H_{22}N_2Se$ [M+Na]: 357.0841, found 357.0833 [M+Na].

N´-adamantyl-N-(methylphenyl)selenourea (**6b**). Yield 94%, mp 169ºC. IR (KBr) ῡ cm-1: 3344 (N-H), 3159 (C_{sp2}-H), 2909 (C_{sp3}-H). ^{1}H NMR (400 MHz, $CDCl_3$) δ: 8.12 (br s, 1H, NH), 7.18 (d, 2H, J = 8.2 Hz, H_3+H_5), 7.06 (d, 2H, J = 7.8 Hz, H_2+H_5), 6.21 (br s, 1H, N´H), 2.31 (s, 3H, Ph-CH_3), 2.20 (s, 6H, $H_{2'}$), 2.07 (s, 3H, $H_{3'}$), 1.64 (s, 6H, $H_{4'}$). ^{13}C NMR (100 MHz, $CDCl_3$) δ: 174.3 (C=Se), 137.6 (C_4), 133.5 (C_1), 130.8 (C_3+C_5), 125.4 (C_2+C_6), 55.5 (Ph-CH_3), 41.7 (C'_2), 36.2 (C'_4), 29.6 (C'_3), 21.11 (Ph-CH_3). ^{77}Se NMR (76 MHz, $CDCl_3$) δ: 267.0 (C=Se). HRMS calcd. For $C_{18}H_{24}N_2Se$ [M+Na]: 371.0998, found 371.0990 [M+Na].

N´-adamantyl-N-(4-methoxyphenyl)selenourea (**6c**). Yield 84%, mp 165ºC. IR (KBr) ῡ cm^{-1}: 3202 (N-H), 3157 (C_{sp2}-H), 2906 (C_{sp3}-H). ^{1}H NMR (400 MHz, DMSO) δ: 9.44 (br s, 1H, NH), 7.20 (d, 2H, $J_{3\text{-}2}$=$J_{5\text{-}6}$ = 8.8 Hz, H_3+H_5), 6.90 (d, 2H, H_2+H_6), 3.74 (s, 3H. OCH_3), 2.24 (s, 6H, $H_{2'}$), 2.04 (s, 3H, $H_{3'}$), 1.63 (s, 6H, $H_{4'}$). ^{13}C NMR (100 MHz, DMSO) δ: 176.0 (C=Se), 156.9 (C_4), 131.9 (C_1), 126.9 (C_3+C_5), 113.9 (C_2+C_6), 55.2 (OCH_3), 54.2 ($C_{1'}$), 40.9 ($C_{2'}$), 35.8 ($C_{4'}$), 29.0 ($C_{3'}$). ^{77}Se NMR (76 MHz, $CDCl_3$) δ: 259.8 (C=Se). HRMS calcd. For $C_{18}H_{24}N_2OSe$ [M+H]: 365.1127, found 365.1171 [M+H].

N´-adamantyl-N-(4-chlorophenyl)selenourea (**6d**). Yield 76%, mp 177ºC. IR (KBr) ῡ cm^{-1}: 3354 (N-H), 3109 (C_{sp2}-H), 2912 (C_{sp3}-H). ^{1}H NMR (400 MHz, DMSO) δ: 9.62 (br s, 1H, NH), 7.71 (s, 1H), 7.41 (d, 2H, J = 8.7 Hz, H2+H6), 7.35 (d, 2H, J = 8.8 Hz, H3+J5), 2.28 (s, 6H, H2´), 2.05 (s, 3H, H3´), 1.64 (s, 6H, H4´). ^{13}C NMR (100 MHz, DMSO) δ: 176.35 (C=Se), 138.62 (C1), 128.51 (C4), 128.29 (C3+C5), 126.07 (C2+C6), 54.54 (C1), 40.74 (C2), 35.83 (C4), 29.03 (C3). ^{77}Se NMR (76 MHz, DMSO) δ: 277.6 (C=Se). HRMS calcd. HRMS calcd. For $C_{17}H_{21}N_2ClSe$ [M+Na]: 391.0449, found 391.0610 [M+Na].

N´-Adamanty-N-(4-cyanophenyl)selenourea (**6e**). Yield 50%. IR (KBr) ῡ cm^{-1}: 3359 (N-H), 3134 (C_{sp2}-H), 2911 (C_{sp3}-H). ^{1}H NMR (400 MHz, DMSO) δ: 9.92 (br s, 1H, NH), 8.17 (br s, 1H, NH), 7.73 (d, 2H, $J_{3\text{-}2}$=$J_{5\text{-}6}$ = 8.8 Hz, H_3+H_5), 7.68 (d, 2H, H_2+H_6), 2.31 (s, 6H, $H_{2'}$), 2.07 (s, 3H, $H_{3'}$), 1.64 (s, 6H, $H_{4'}$). ^{13}C NMR (100 MHz, DMSO) δ: 176.1 (C=Se), 144.3 (C_1), 132.7 (C_3+C_5), 122.6 (C_2+C_6), 120.0 (C_4), 105.1 (CN), 54.9 ($C_{1'}$), 40.5 ($C_{2'}$), 35.8 ($C_{4'}$), 29.0 ($C_{3'}$). ^{77}Se NMR (76 MHz, DMSO) δ: 312.7 (C=Se). HRMS calcd. For $C_{18}H_{21}N_3Se$ [M+Na]: 382.0793, found 382.0788 [M+Na].

3. NMR SPECTRA (^{1}H, ^{13}C AND ^{77}Se) OF FINAL PRODUCTS

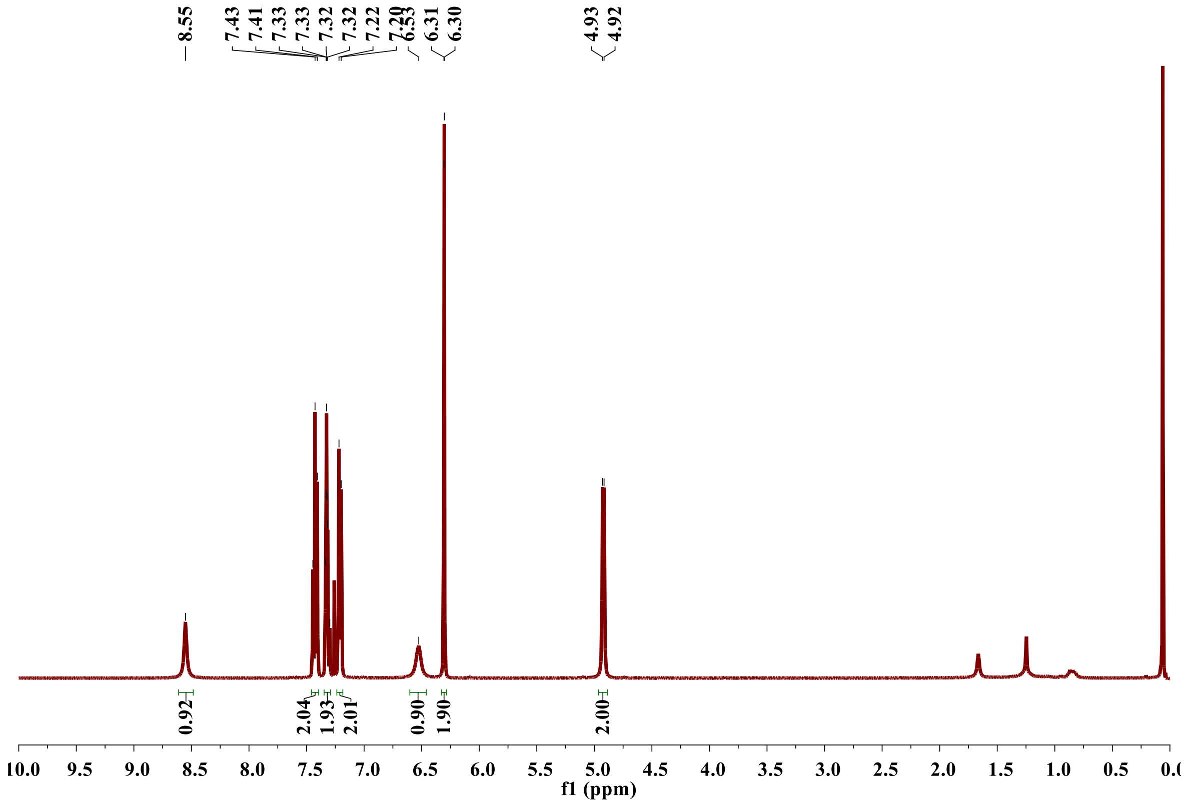

Figure S6. ^{1}H-NMR of compound **1a**.

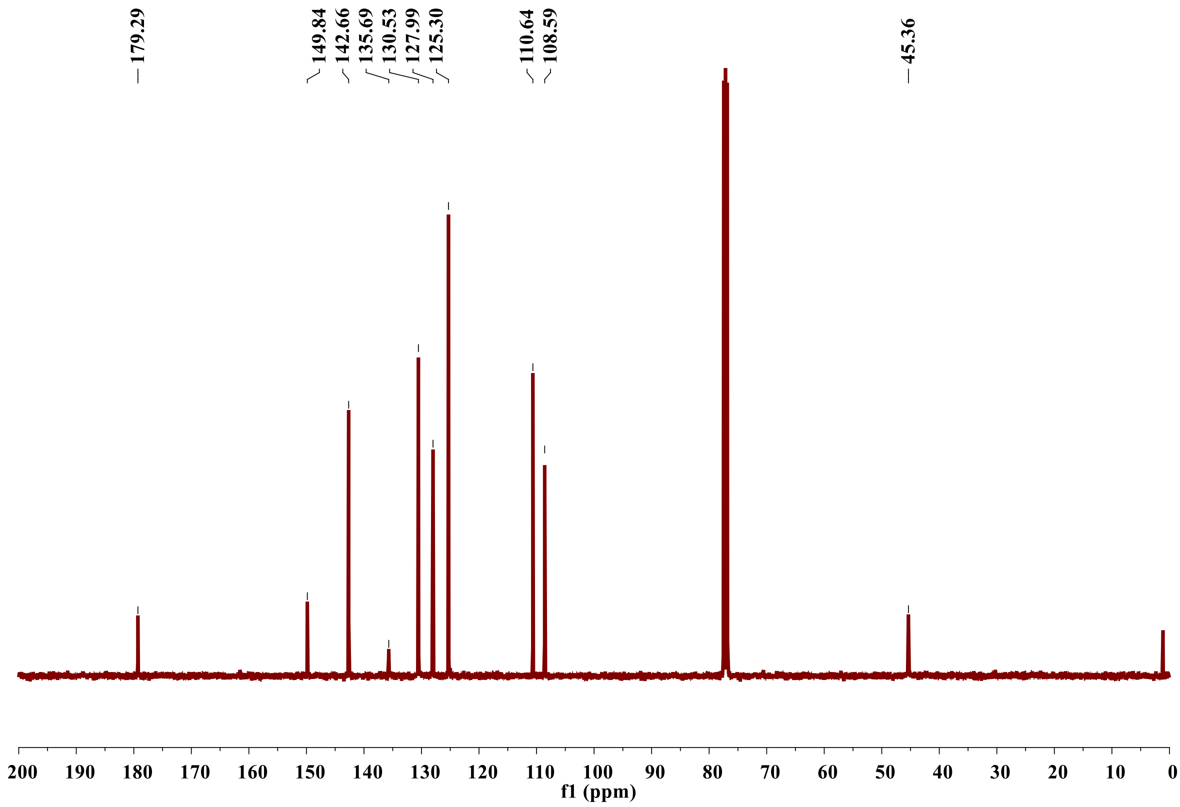

Figure S7. ^{13}C-NMR of compound **1a**.

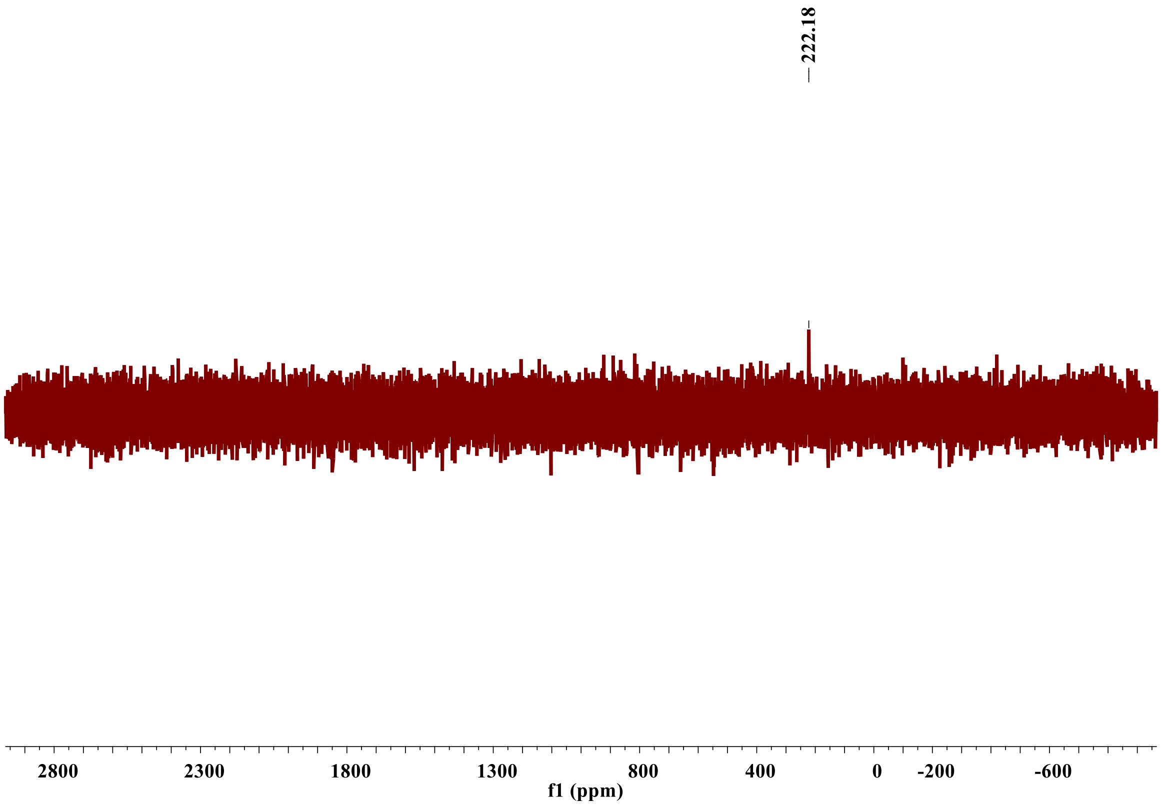

Figure S8. ^{77}Se-NMR of compound **1a**.

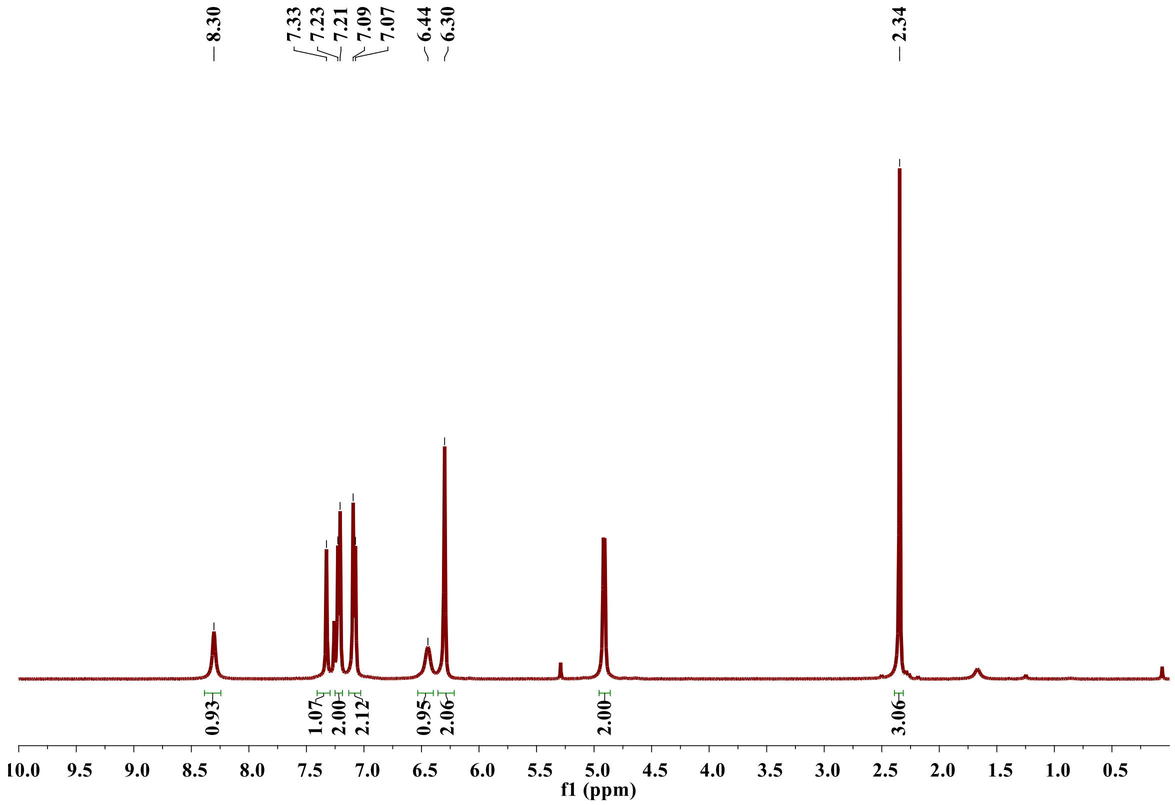

Figure S9. ^{1}H-NMR of compound **1b**.

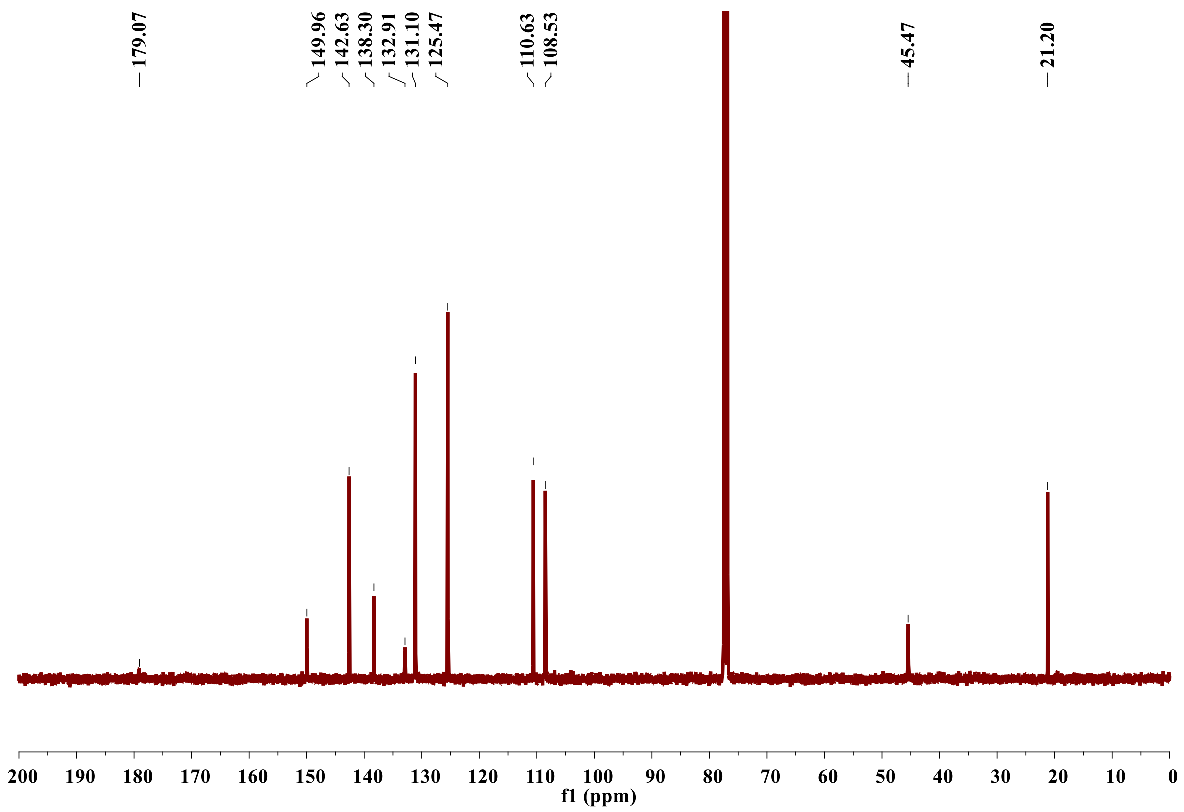

Figure S10. ^{13}C-NMR of compound **1b**.

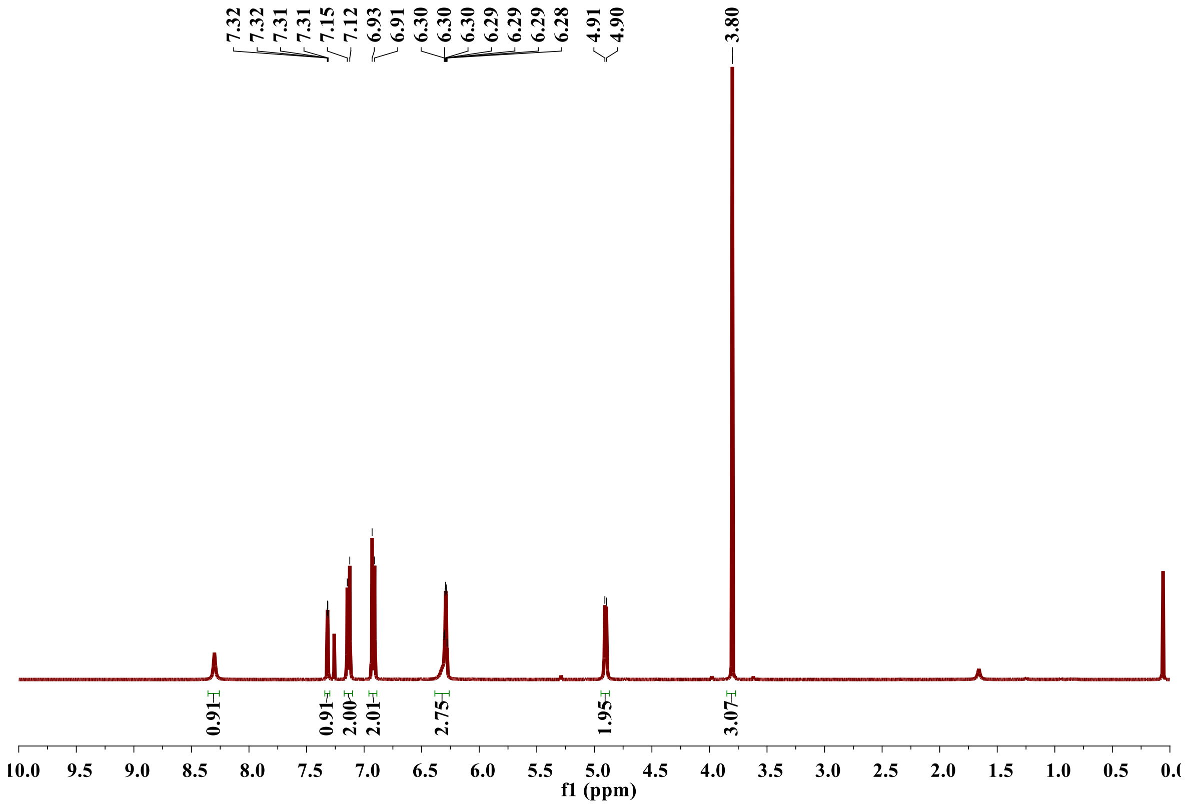

Figure S11. ^{1}H-NMR of compound **1c**.

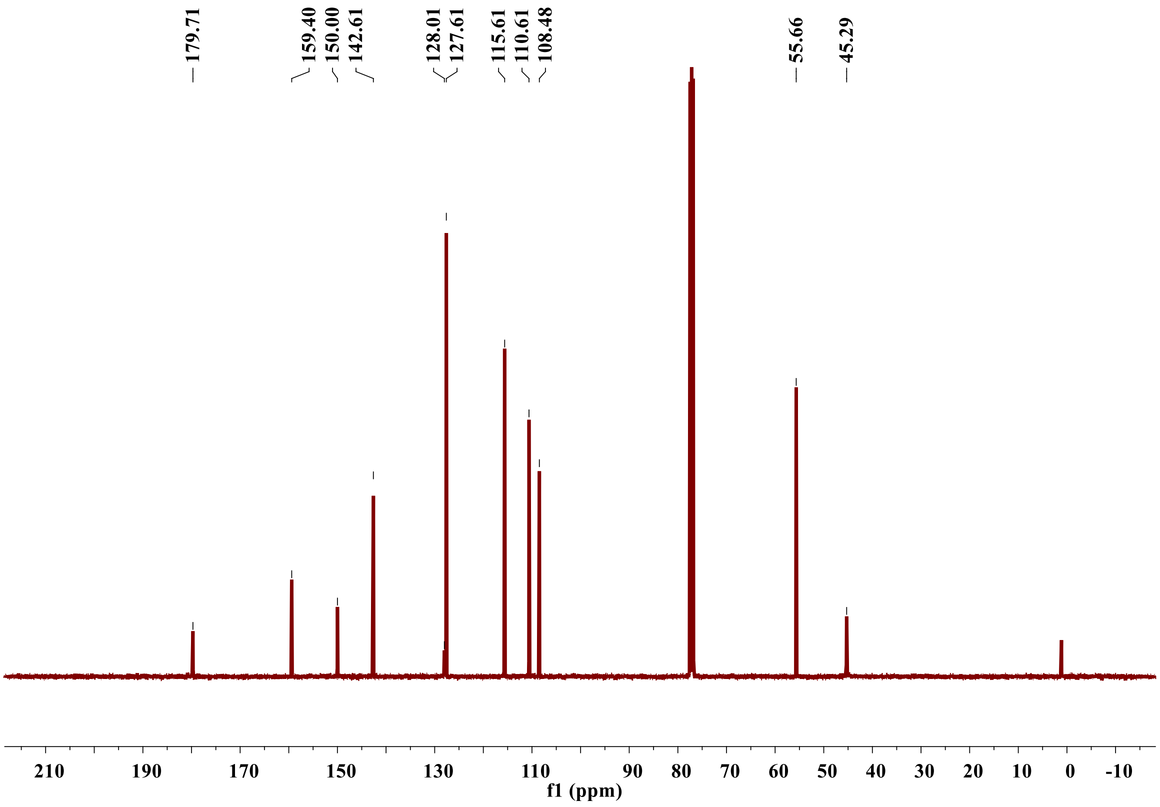

Figure S12. ^{13}C-NMR of compound **1c**.

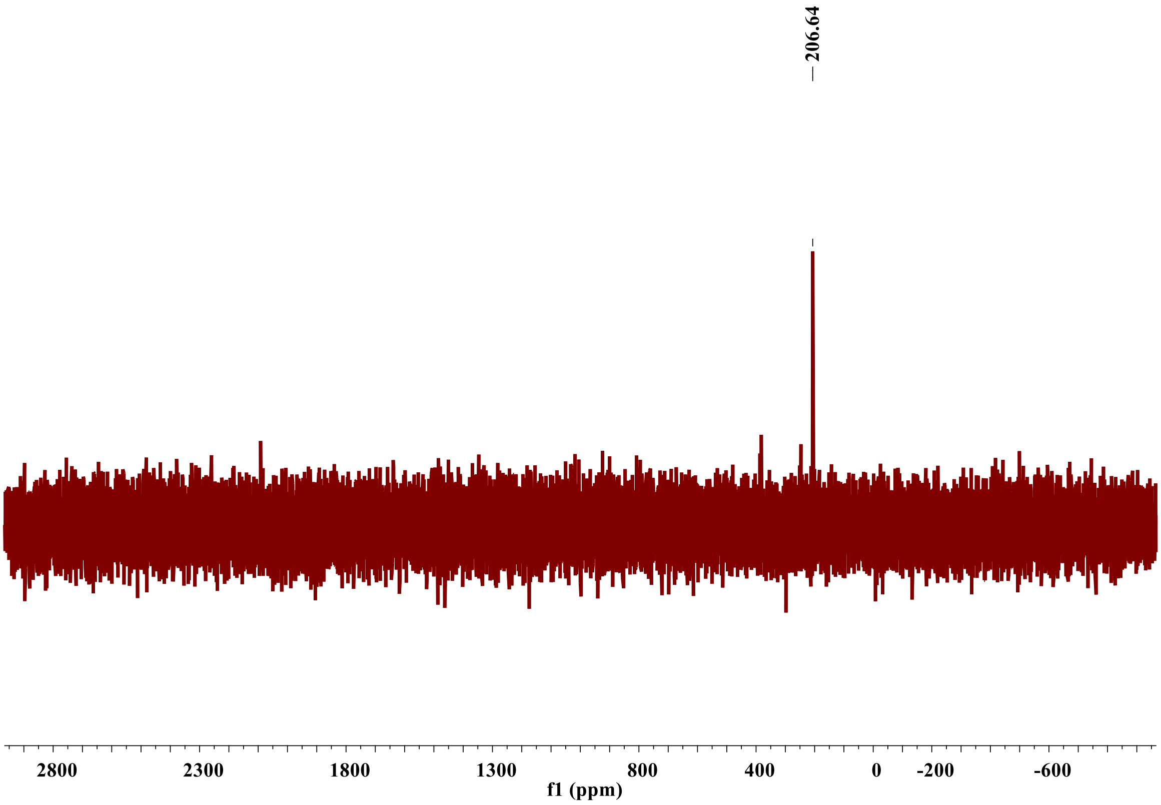

Figure S13. ^{77}Se-NMR of compound **1c**.

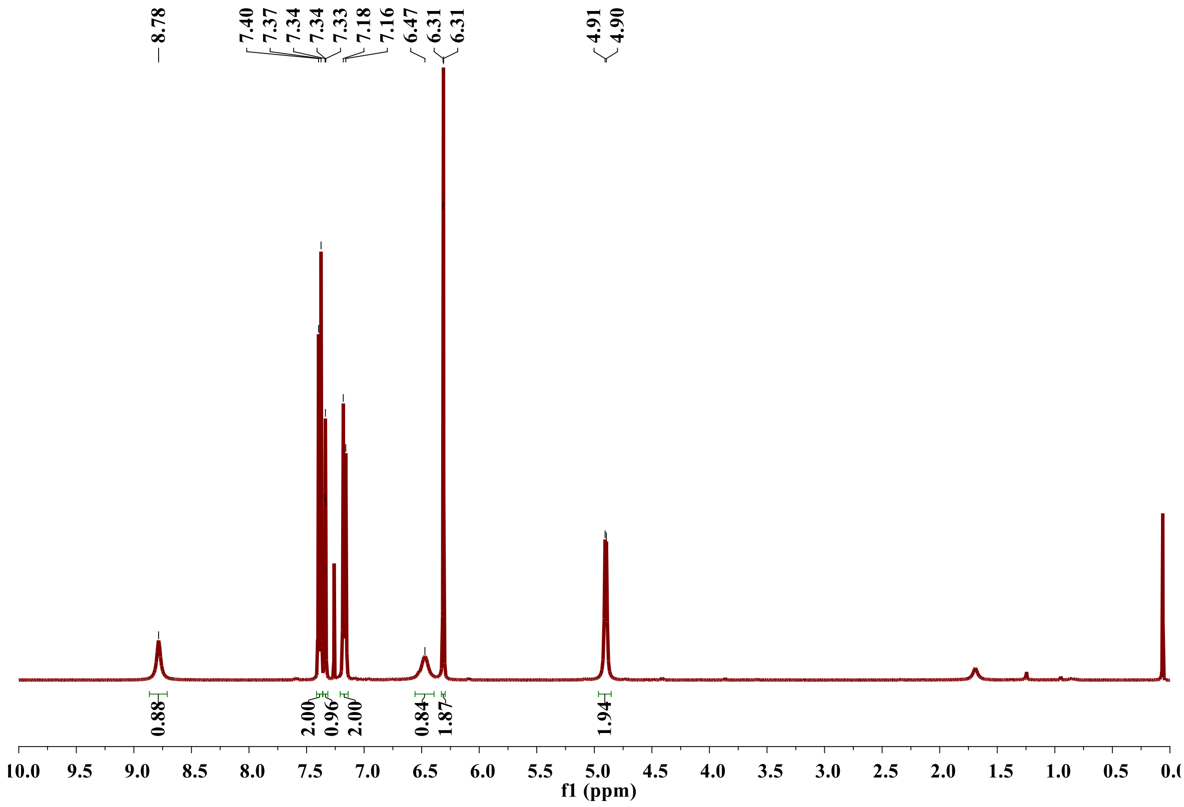

Figure S14. ^{1}H-NMR of compound **1d**.

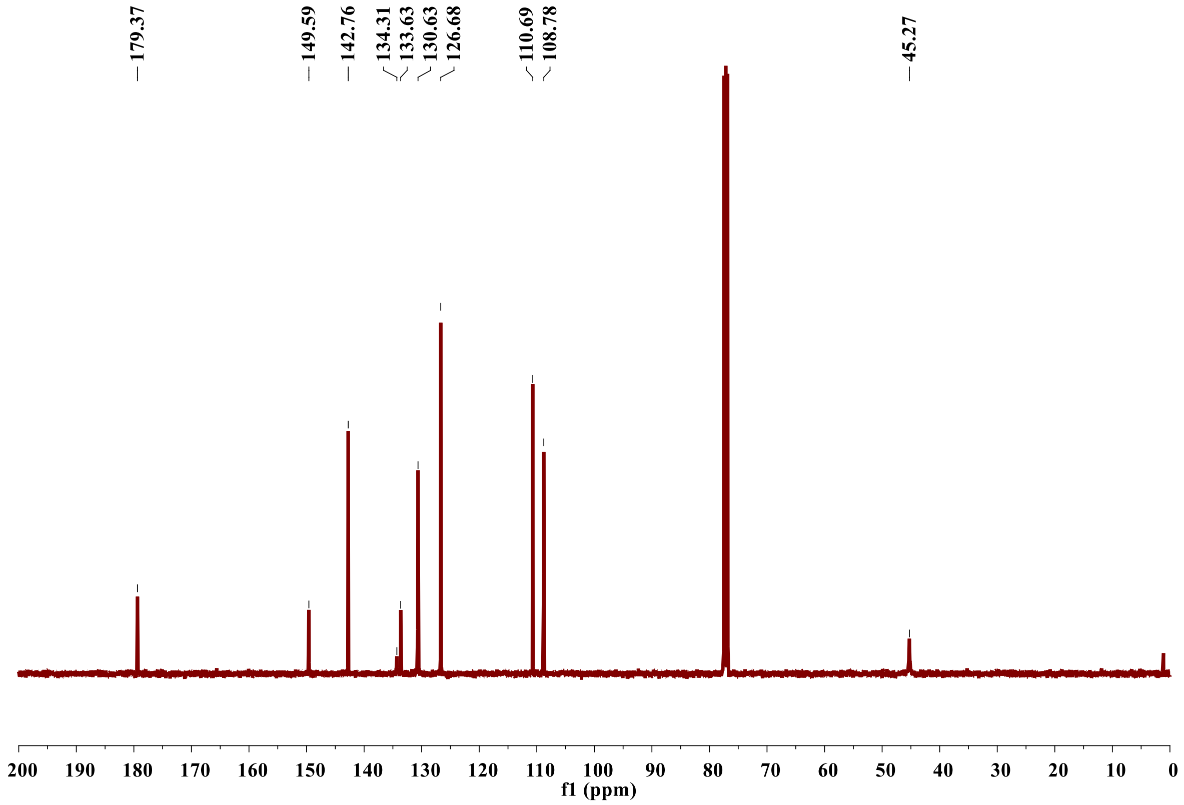

Figure S15. ^{13}C-NMR of compound **1d**.

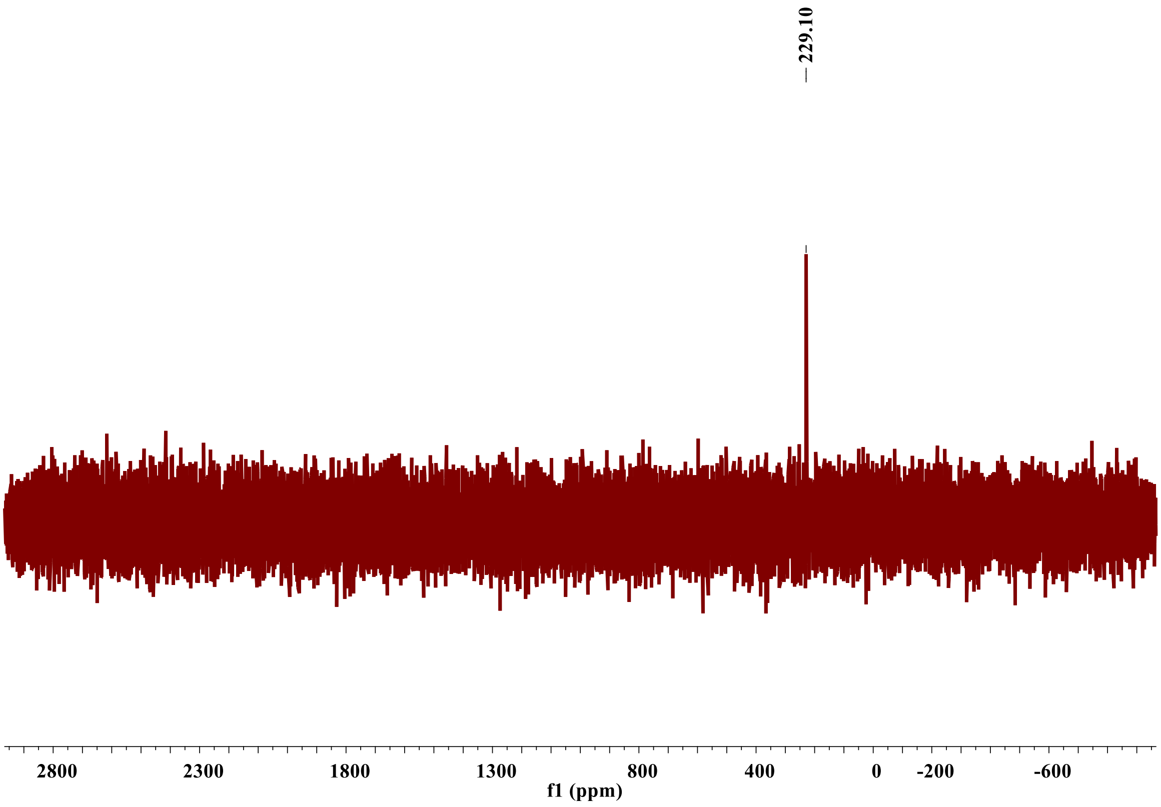

Figure S16. ^{77}Se-NMR of compound **1d**.

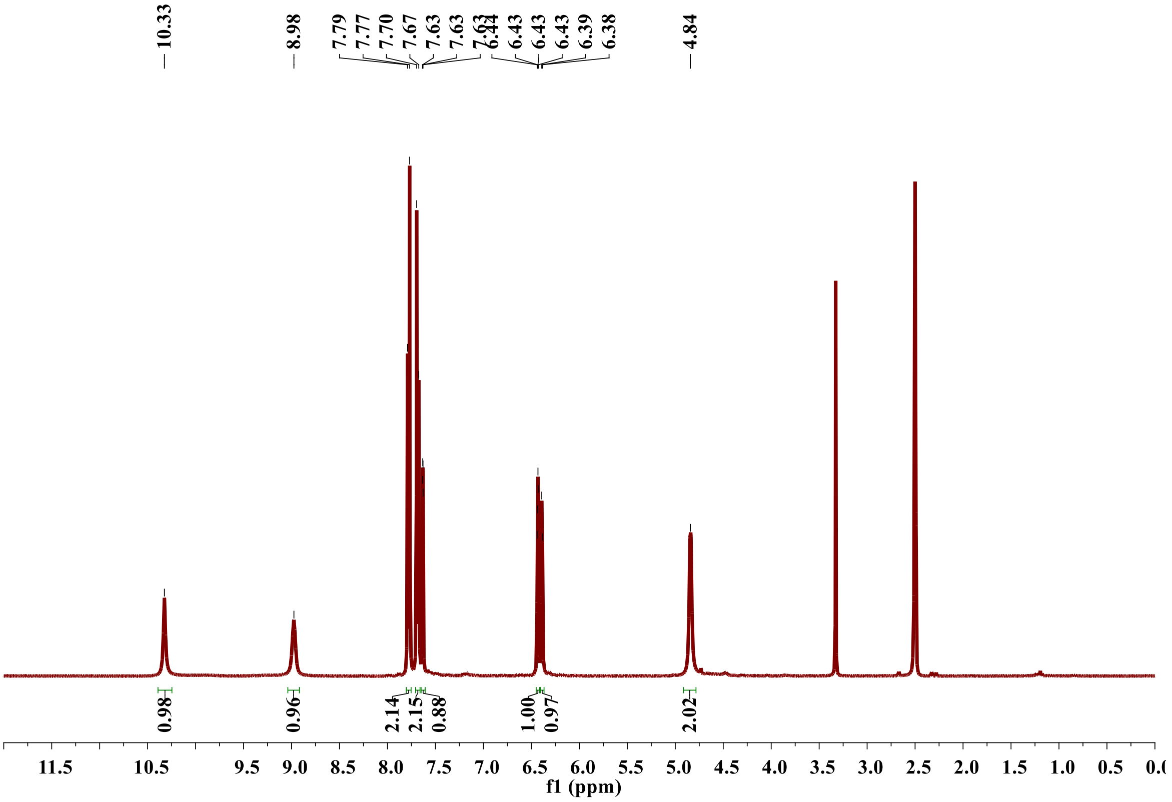

Figure S17. ^{1}H-NMR of compound **1e**.

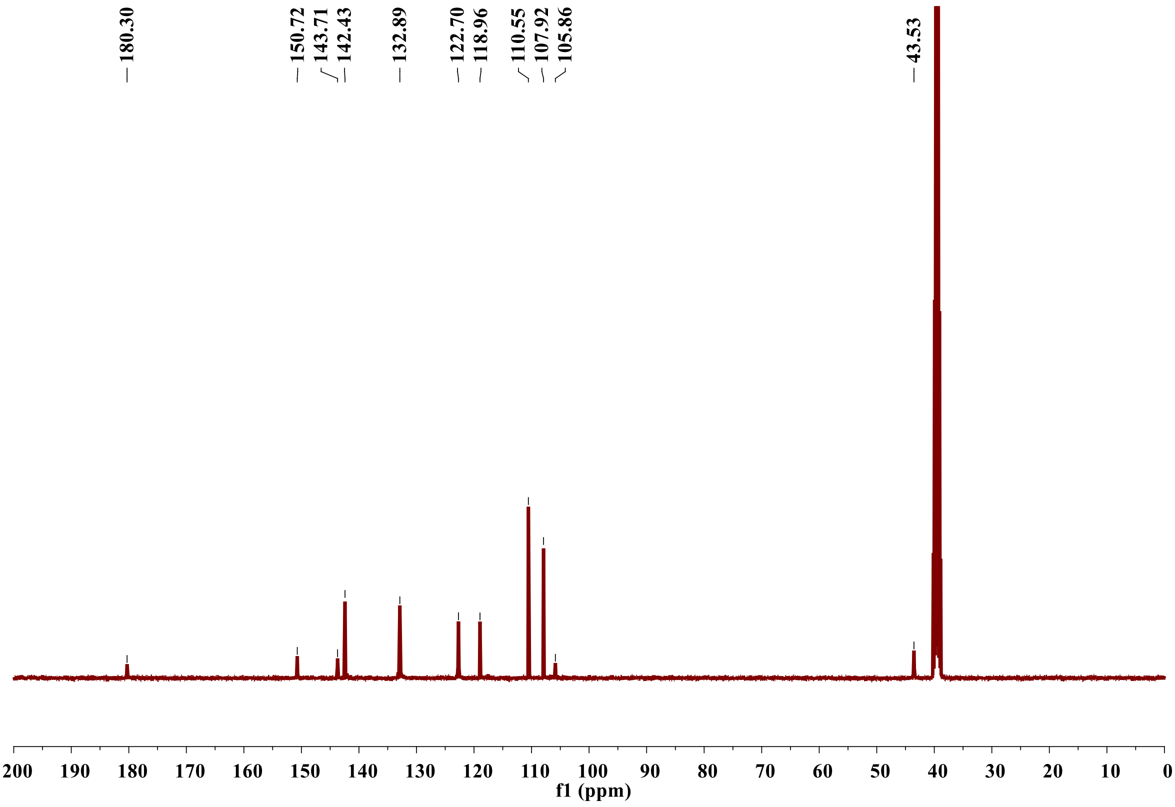

Figure S18. ^{13}C-NMR of compound **1e**.

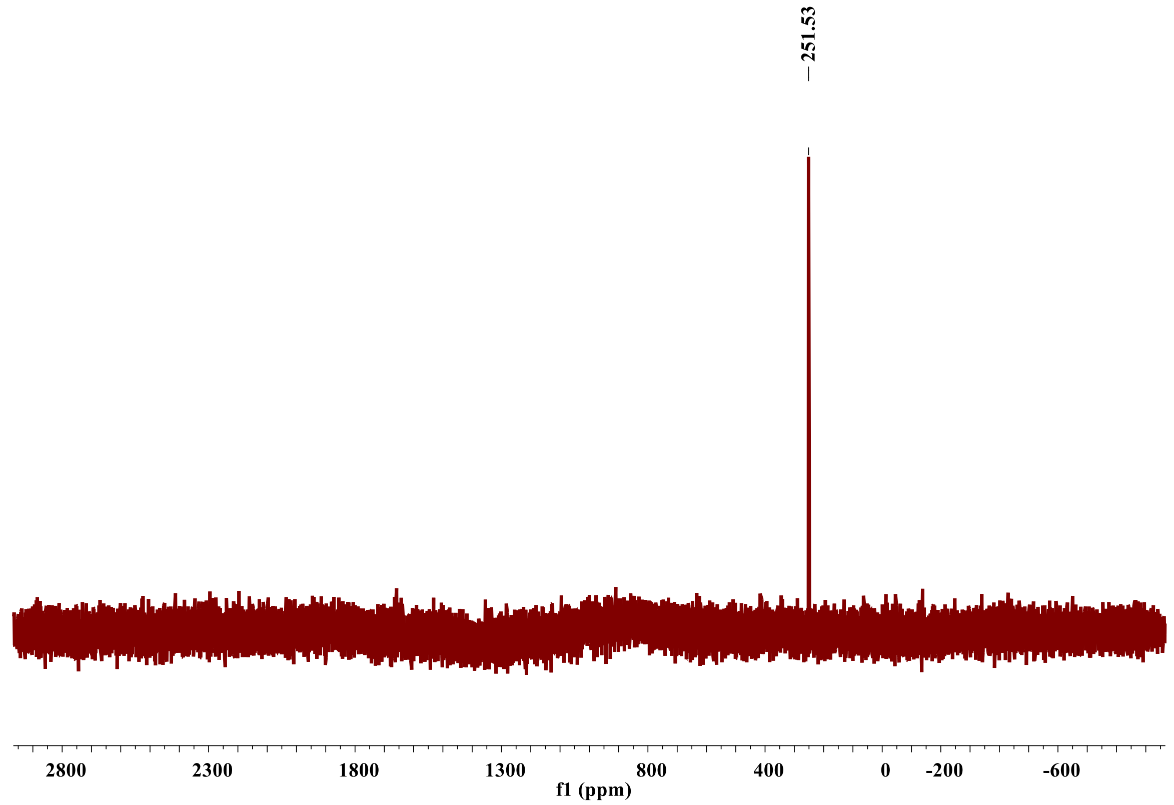

Figure S19. ^{77}Se-NMR of compound **1e**.

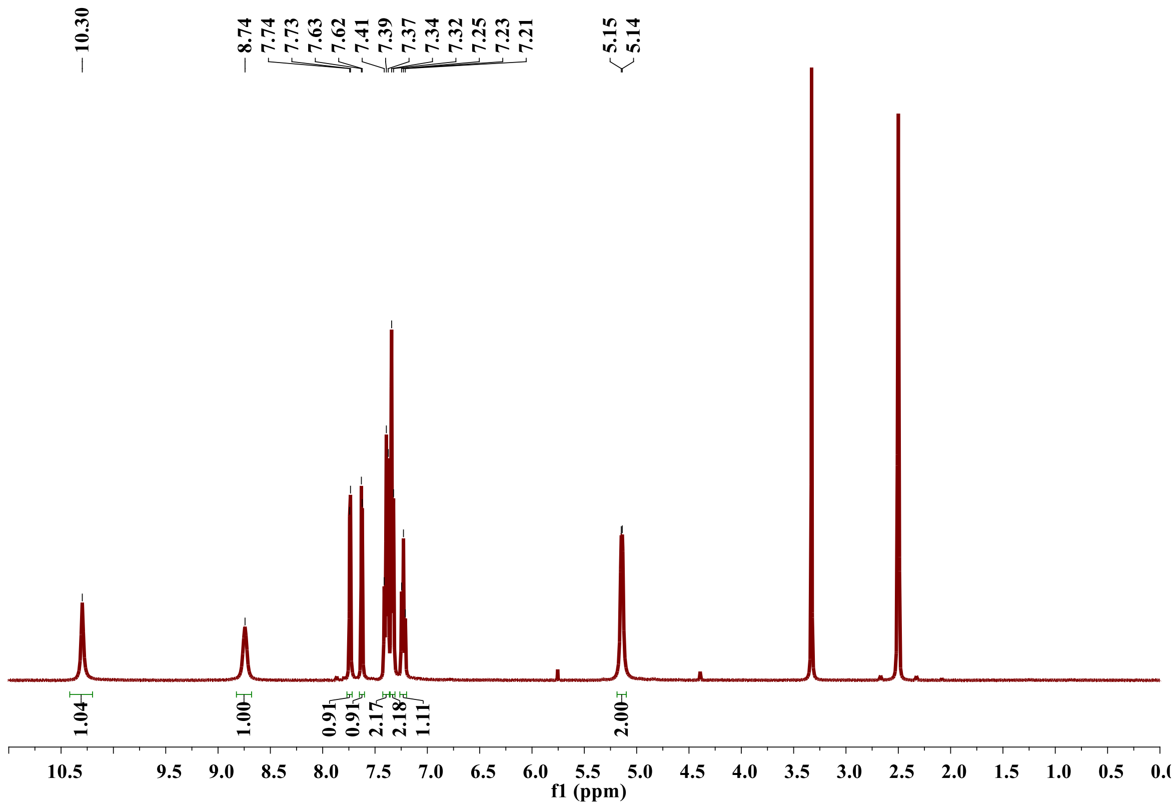

Figure S20. ^{1}H-NMR of compound **2a**.

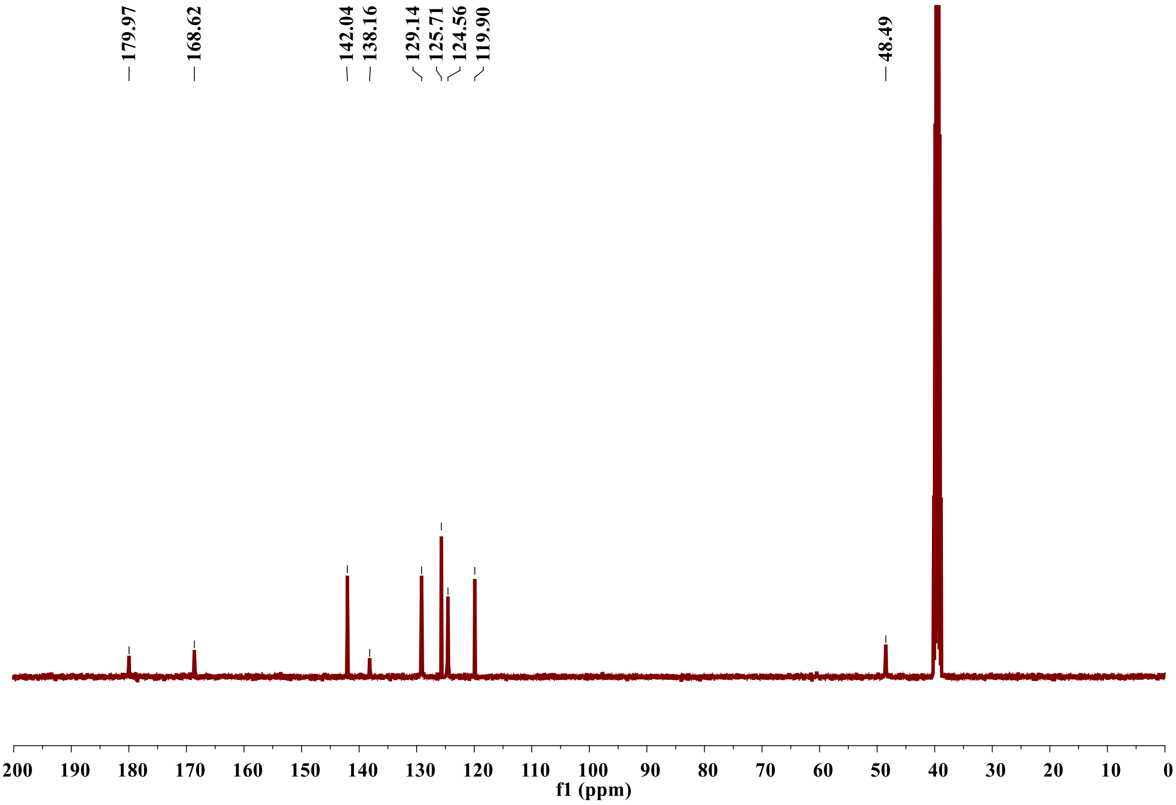

Figure S21. ^{13}C-NMR of compound **2a**.

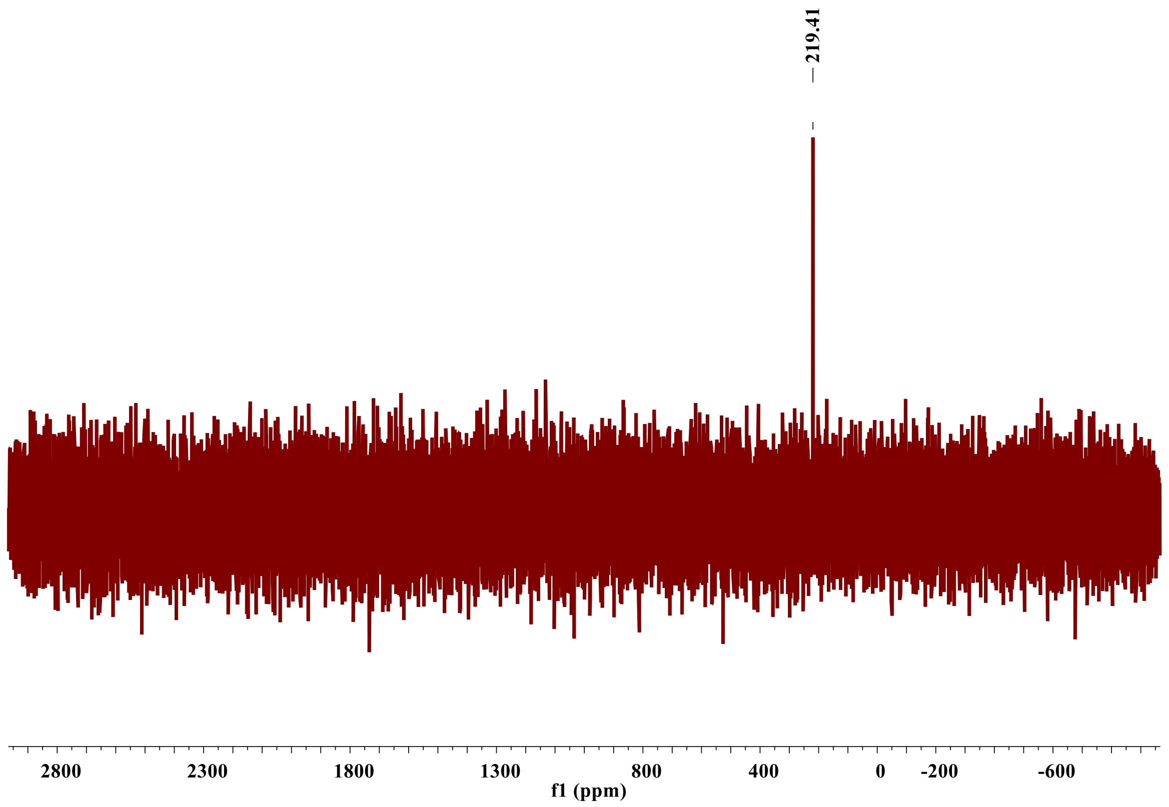

Figure S22. ^{77}Se-NMR of compound **2a**.

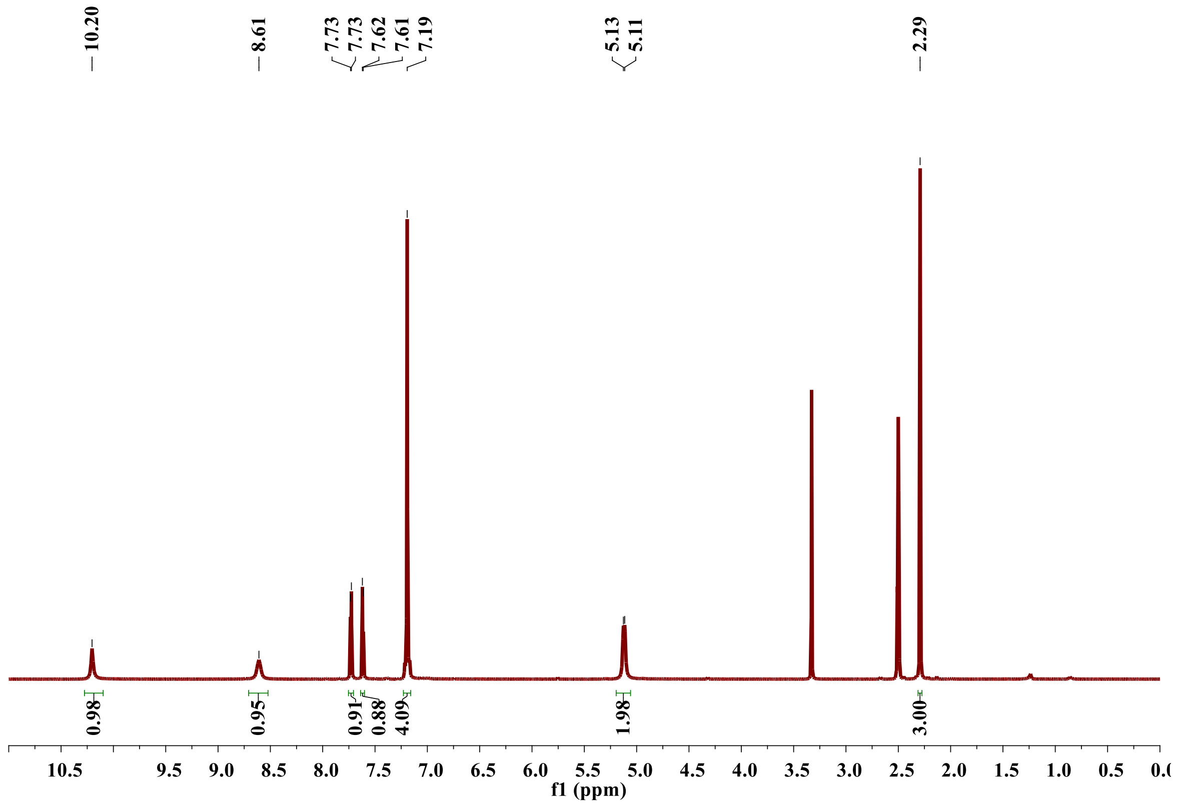

Figure S23. ^{1}H-NMR of compound **2b**.

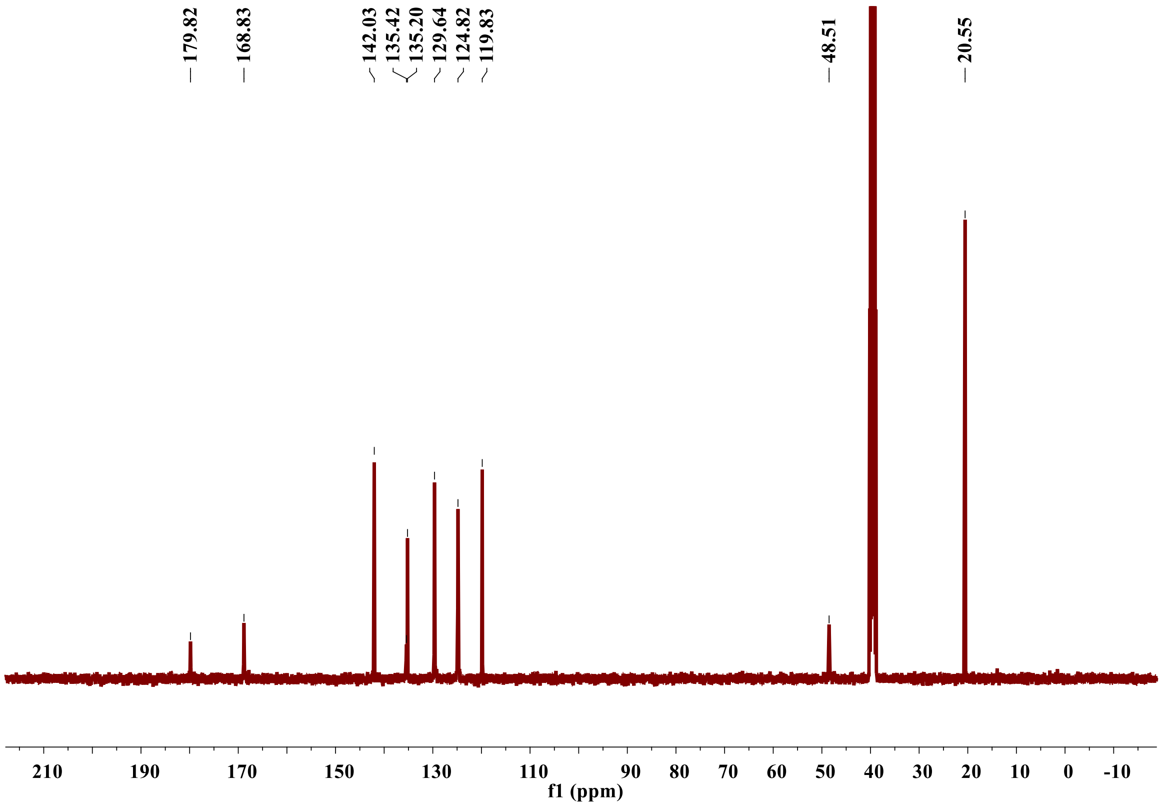

Figure S24. ^{13}C-NMR of compound **2b**.

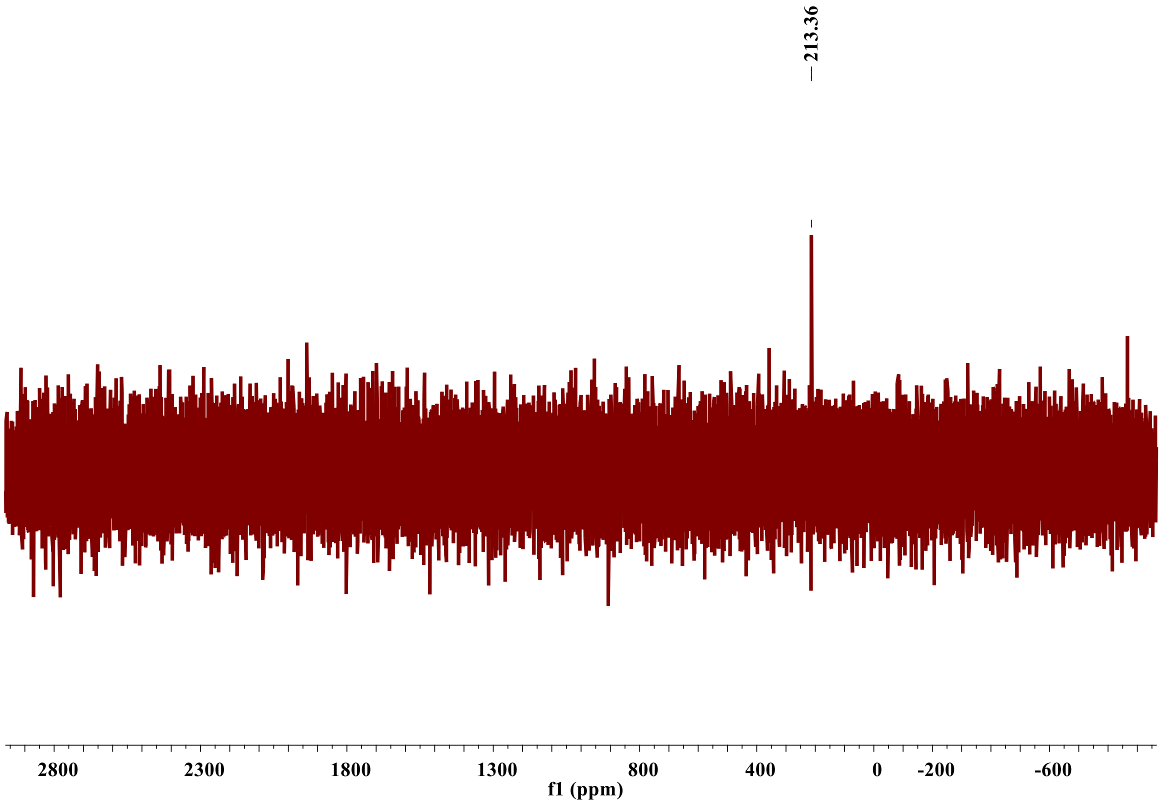

Figure S25. ^{77}Se-NMR of compound **2b**.

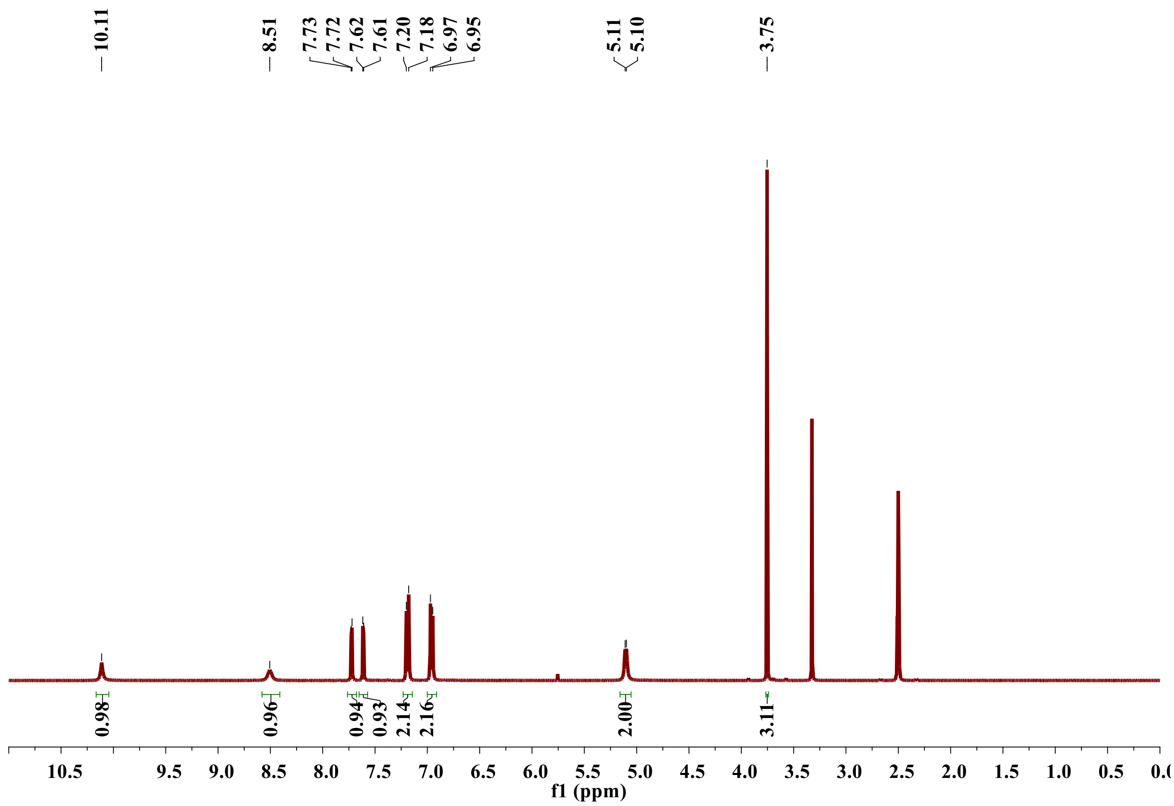

Figure S26. ¹H-NMR of compound **2c**.

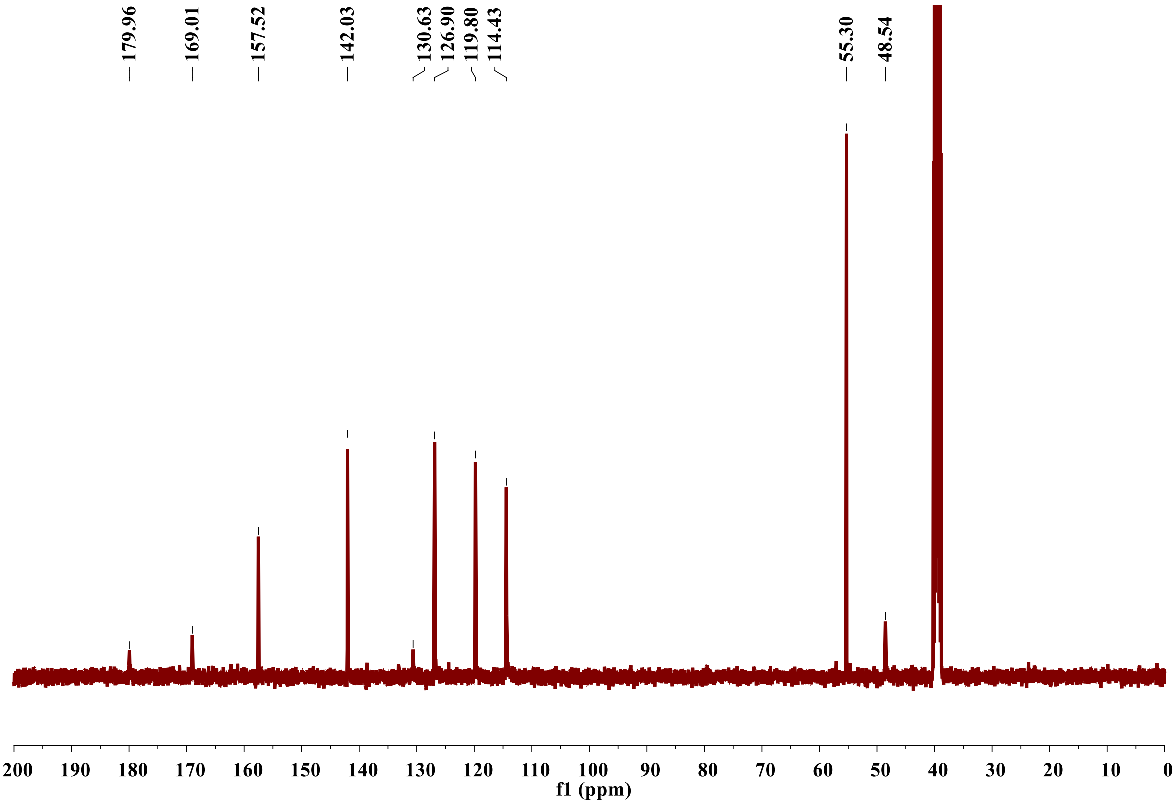

Figure S27. ¹³C-NMR of compound **2c**.

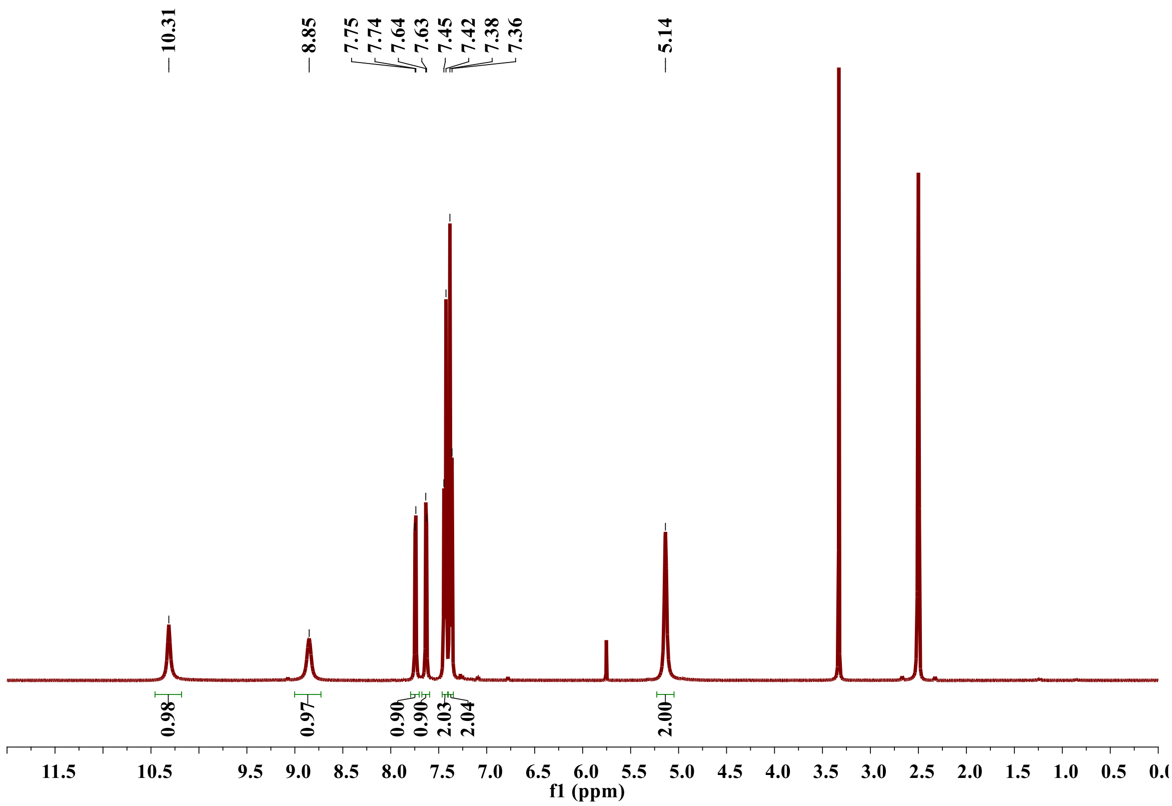

Figure S28. ^{1}H-NMR of compound **2d**.

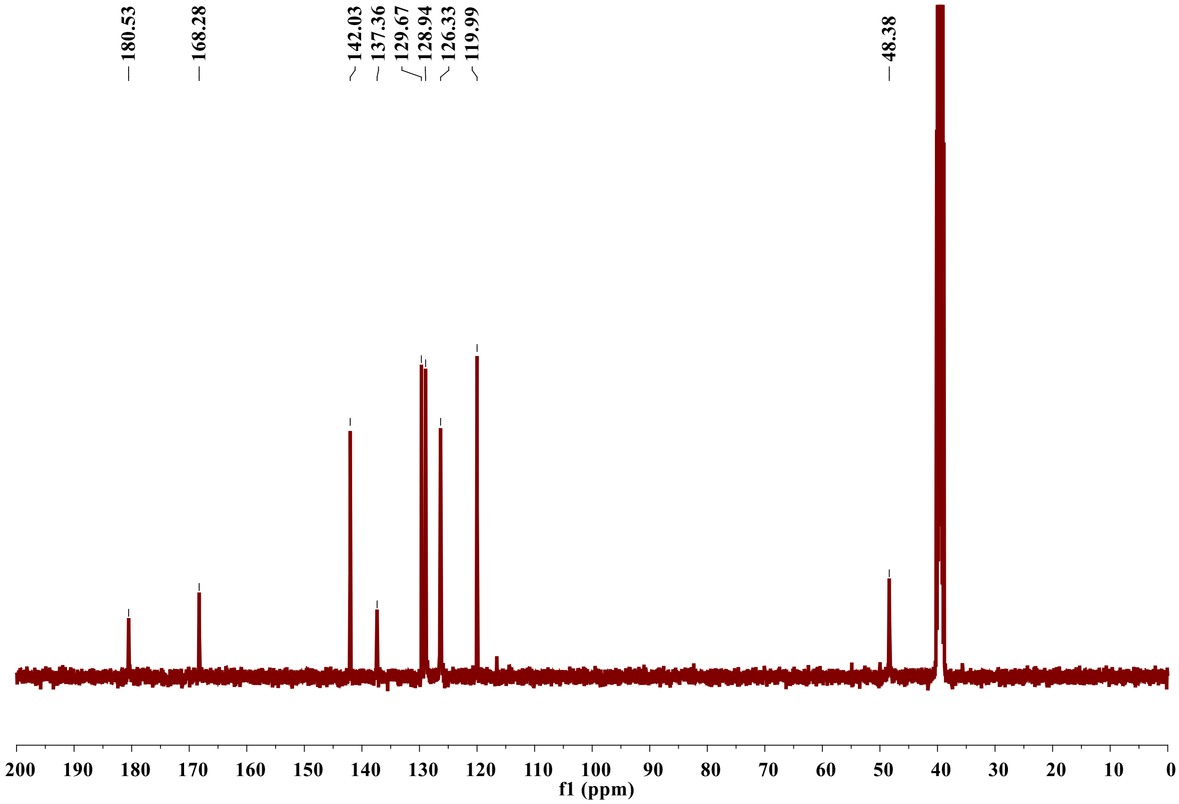

Figure S29. ^{13}C-NMR of compound **2d**.

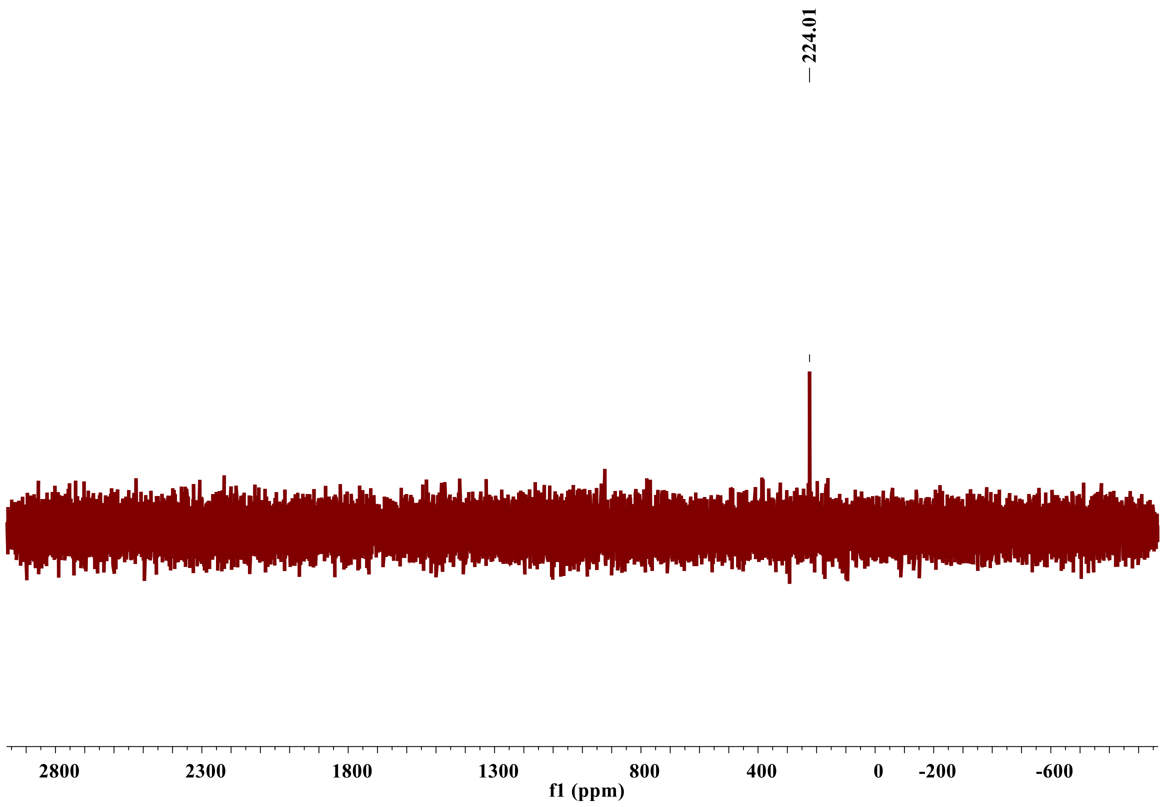

Figure S30. ^{77}Se-NMR of compound **2d**.

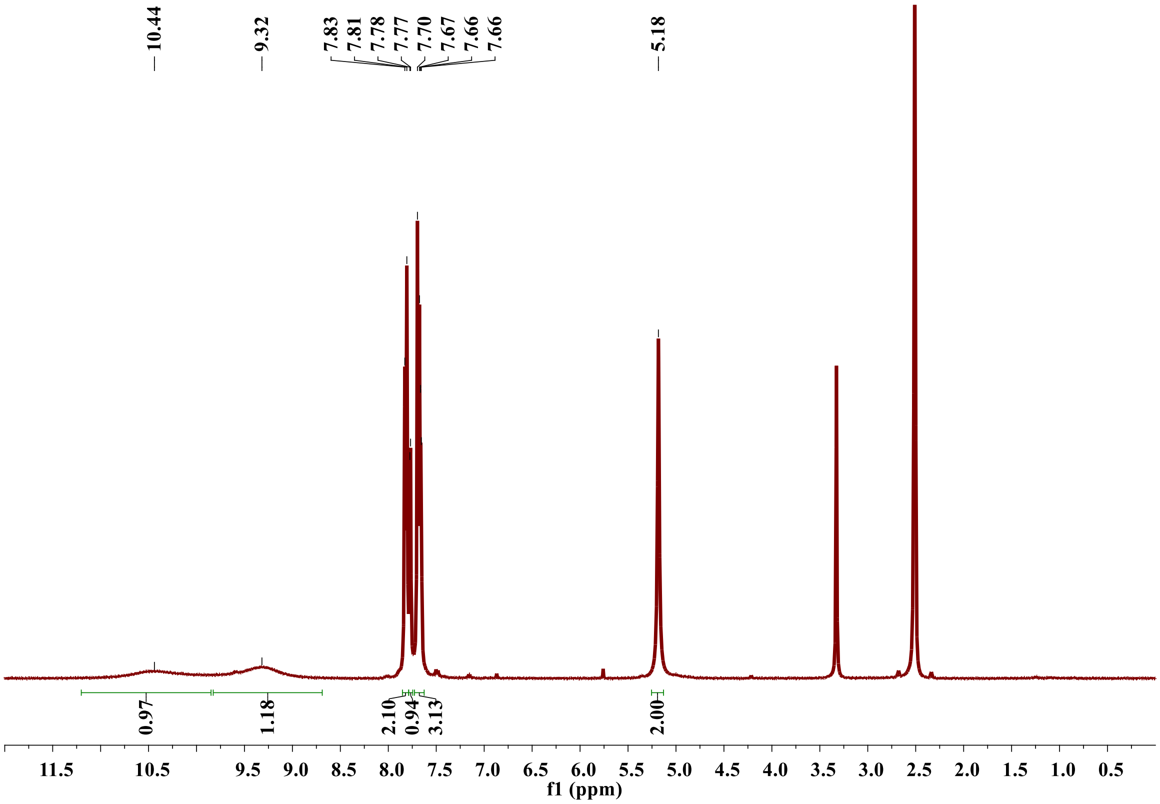

Figure S31. ^{1}H-NMR of compound **2e**.

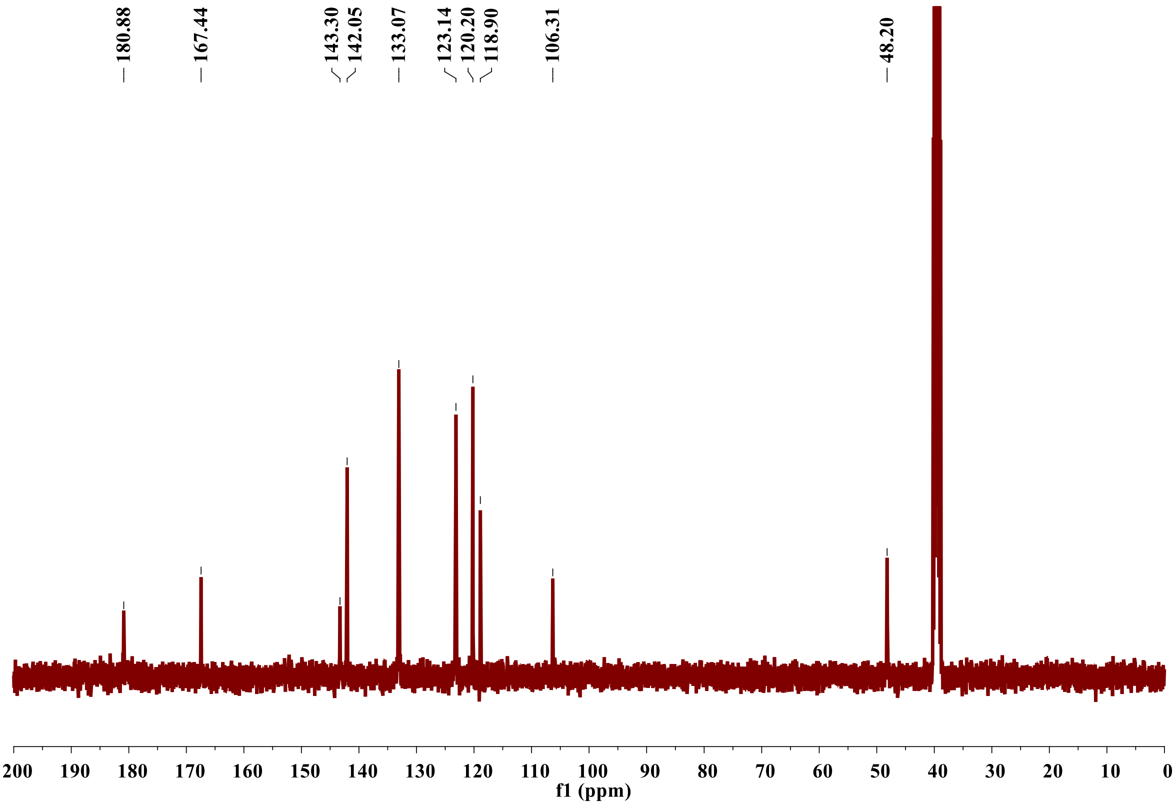

Figure S32. ^{13}C-NMR of compound **2e**.

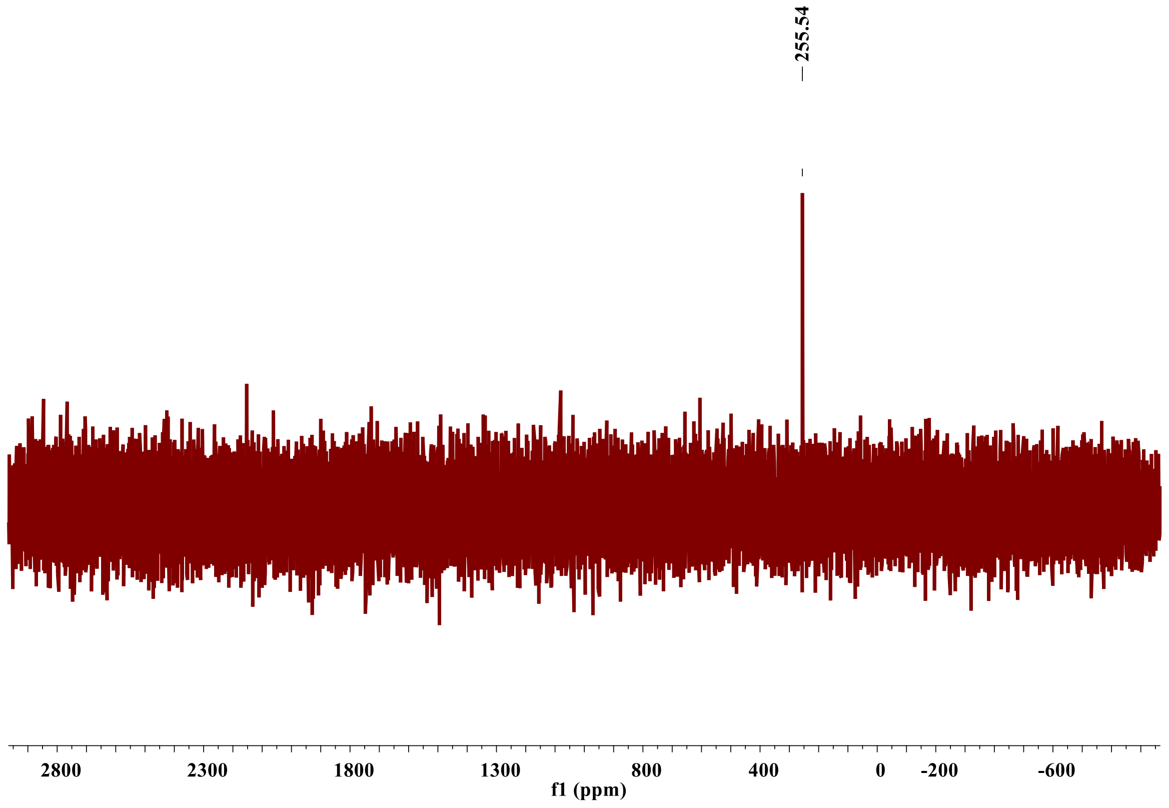

Figure S33. ^{77}Se-NMR of compound **2e**.

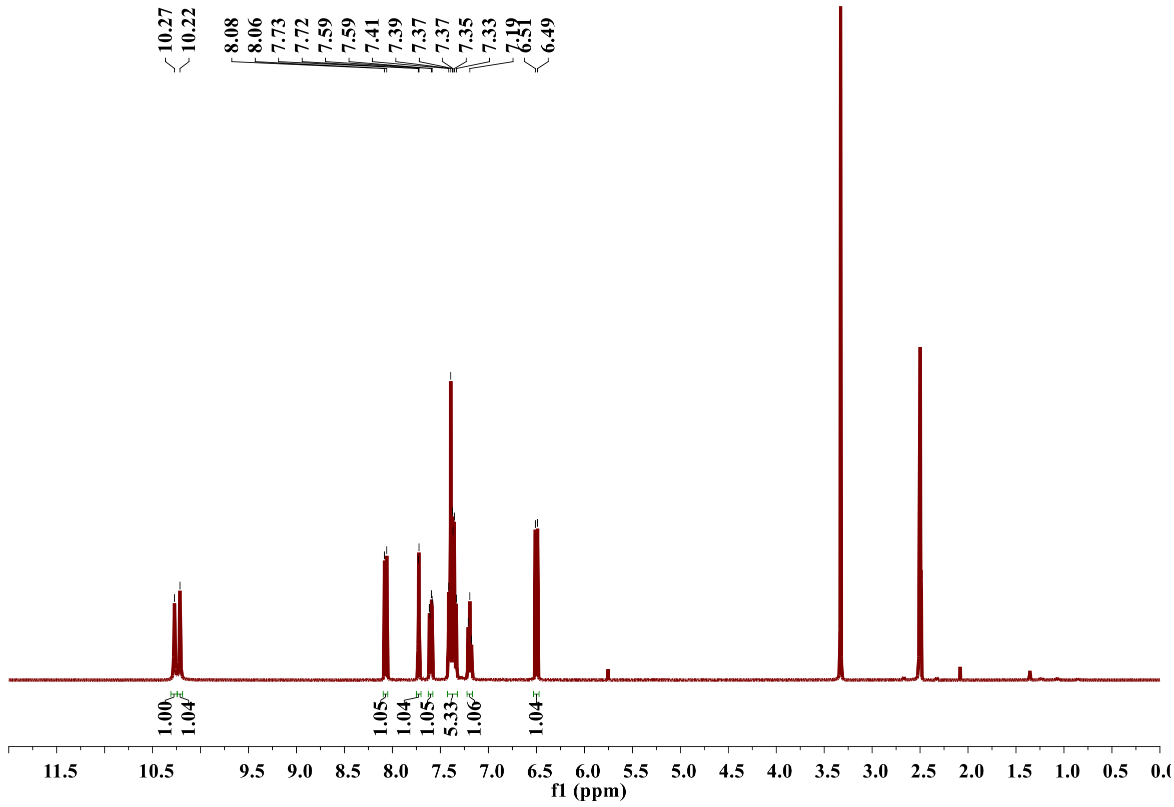

Figure S34. ^{1}H-NMR of compound **3a**.

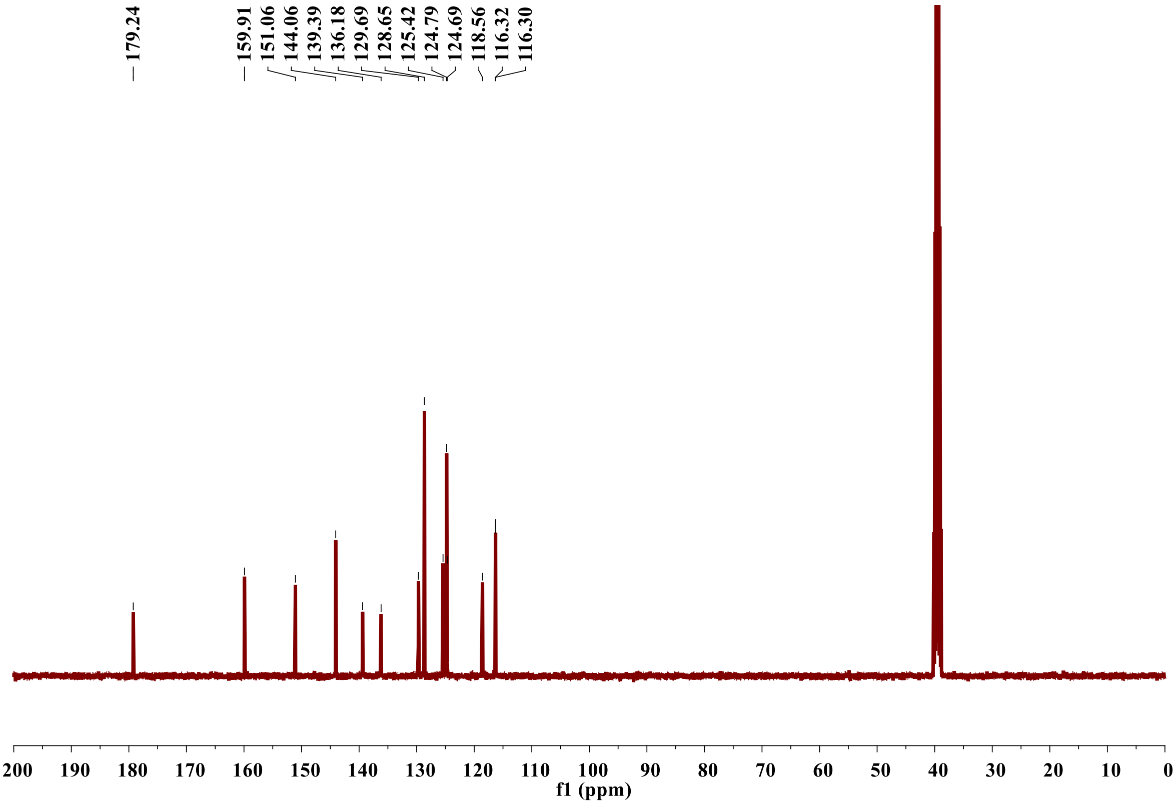

Figure S35. ^{13}C-NMR of compound **3a**.

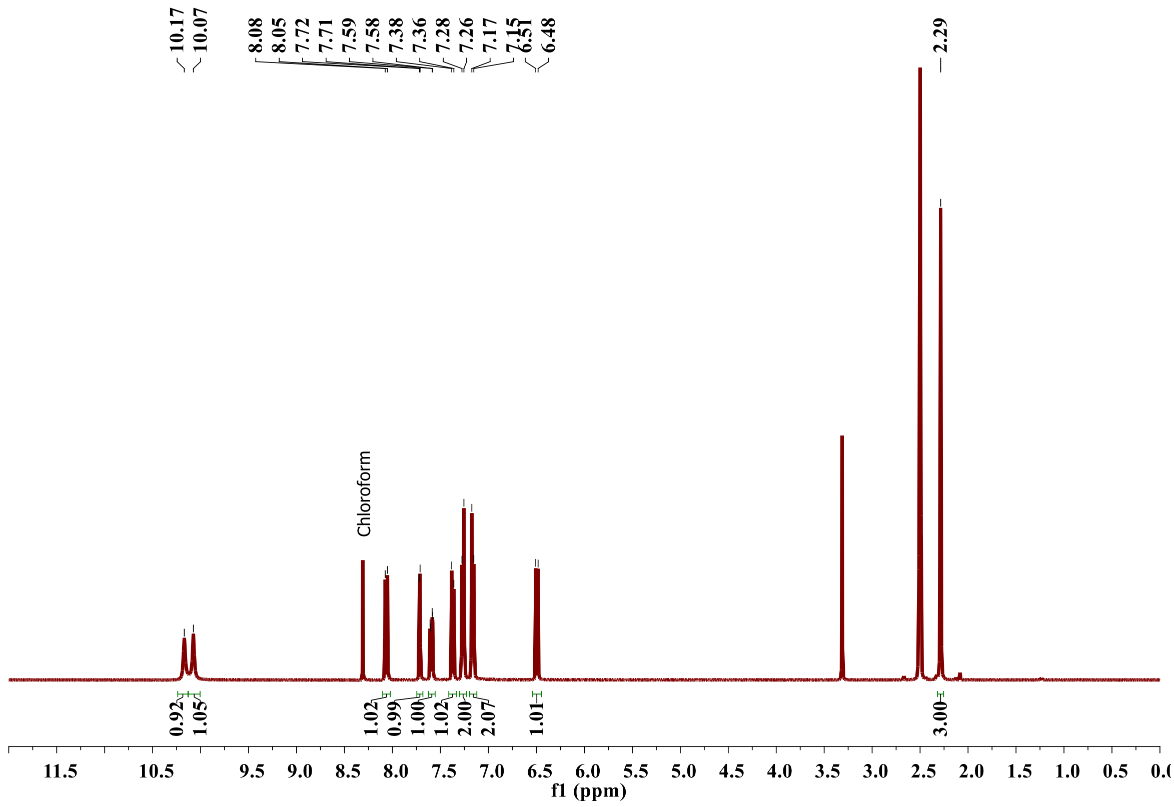

Figure S36. ^{1}H-NMR of compound **3b**.

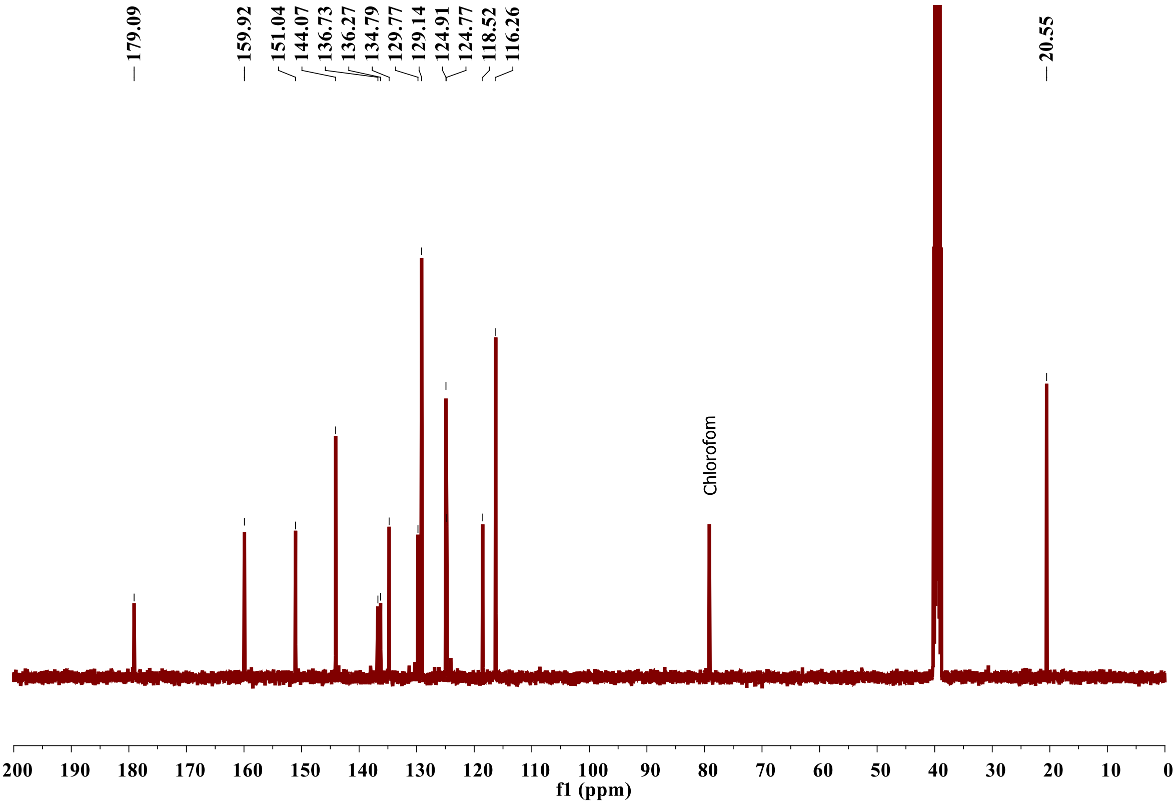

Figure S37. ^{13}C-NMR of compound **3b**.

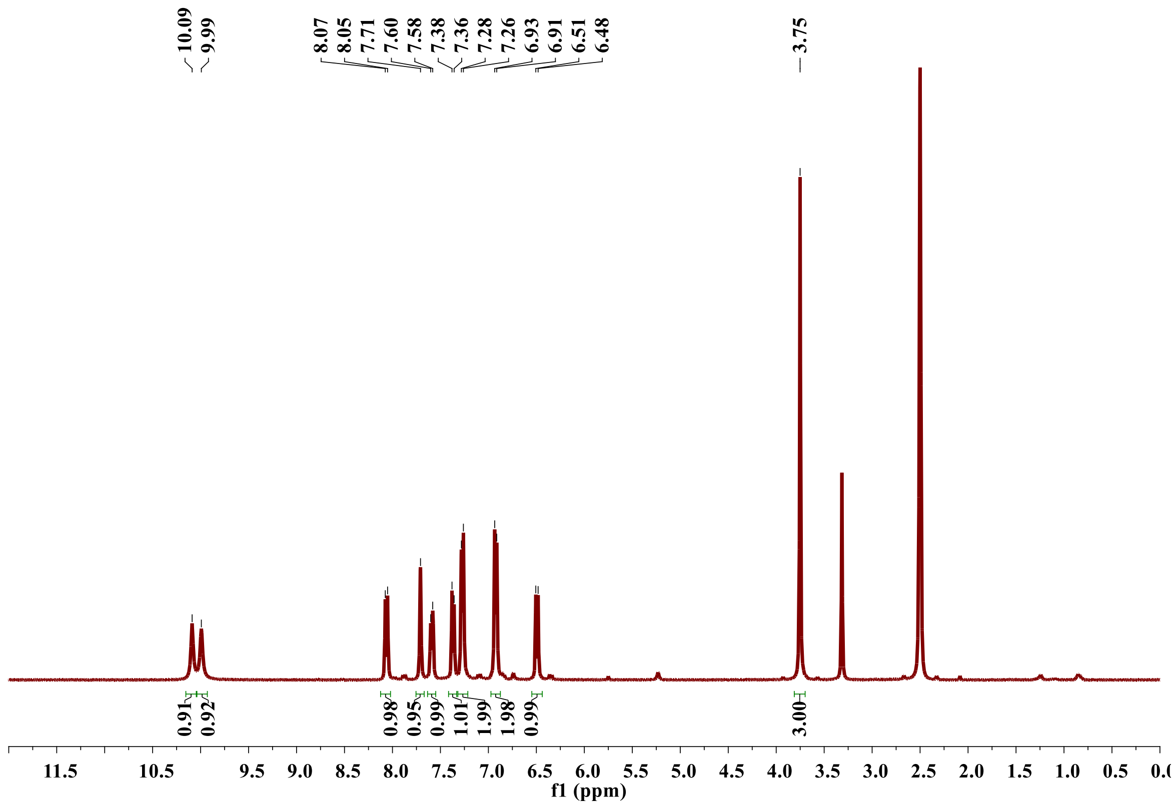

Figure S38. ^{1}H-NMR of compound **3c**.

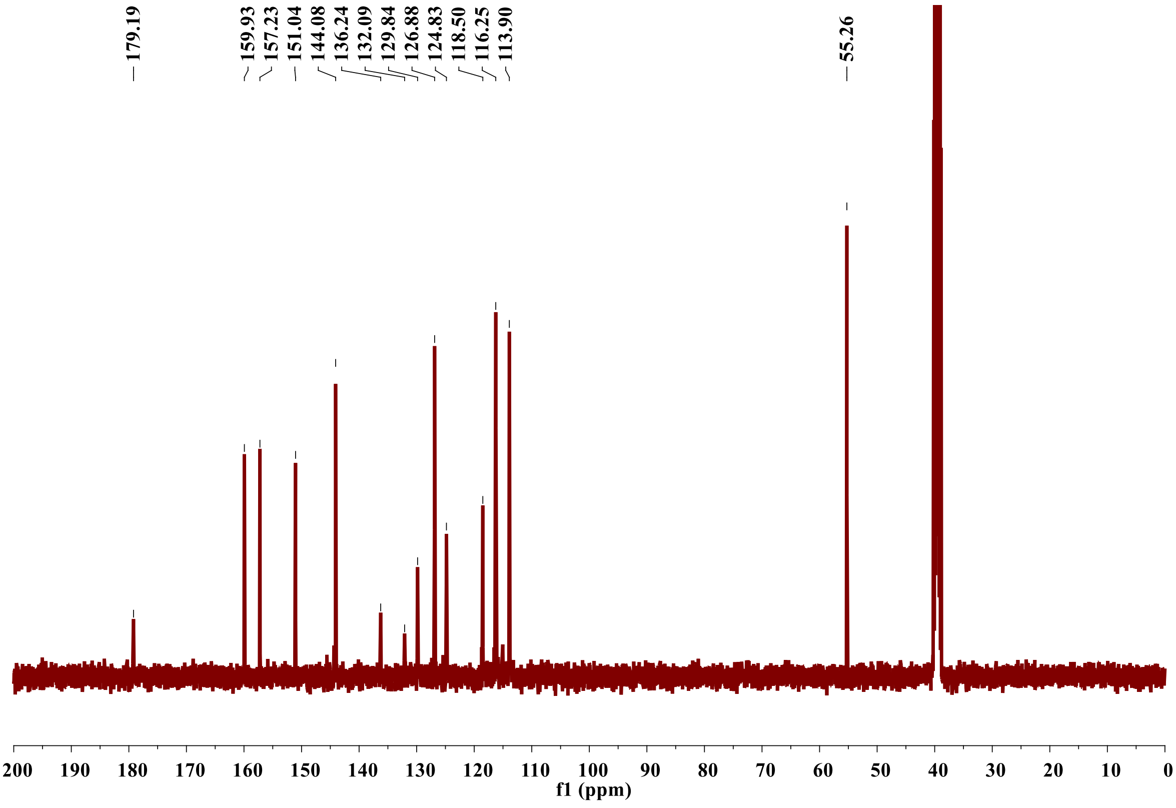

Figure S39. ^{13}C-NMR of compound **3c**.

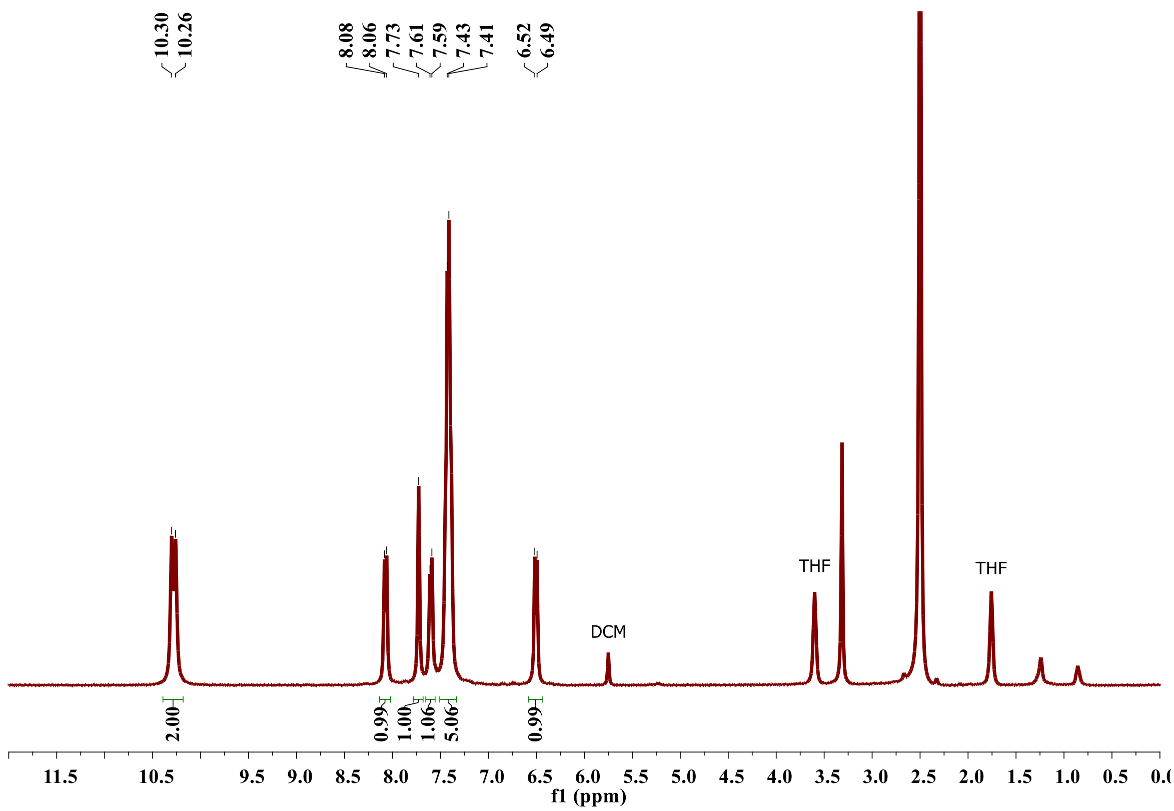

Figure S40. ^{1}H-NMR of compound **3d**.

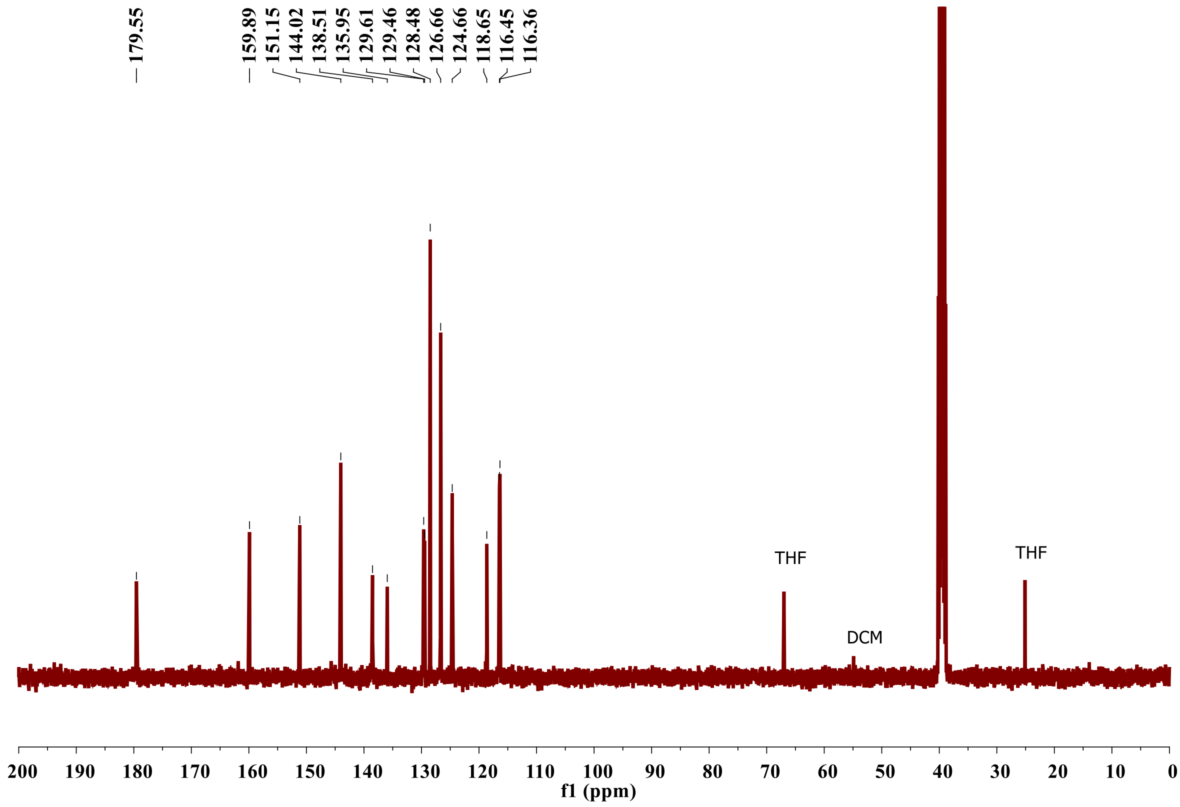

Figure S41. ^{13}C-NMR of compound **3d**.

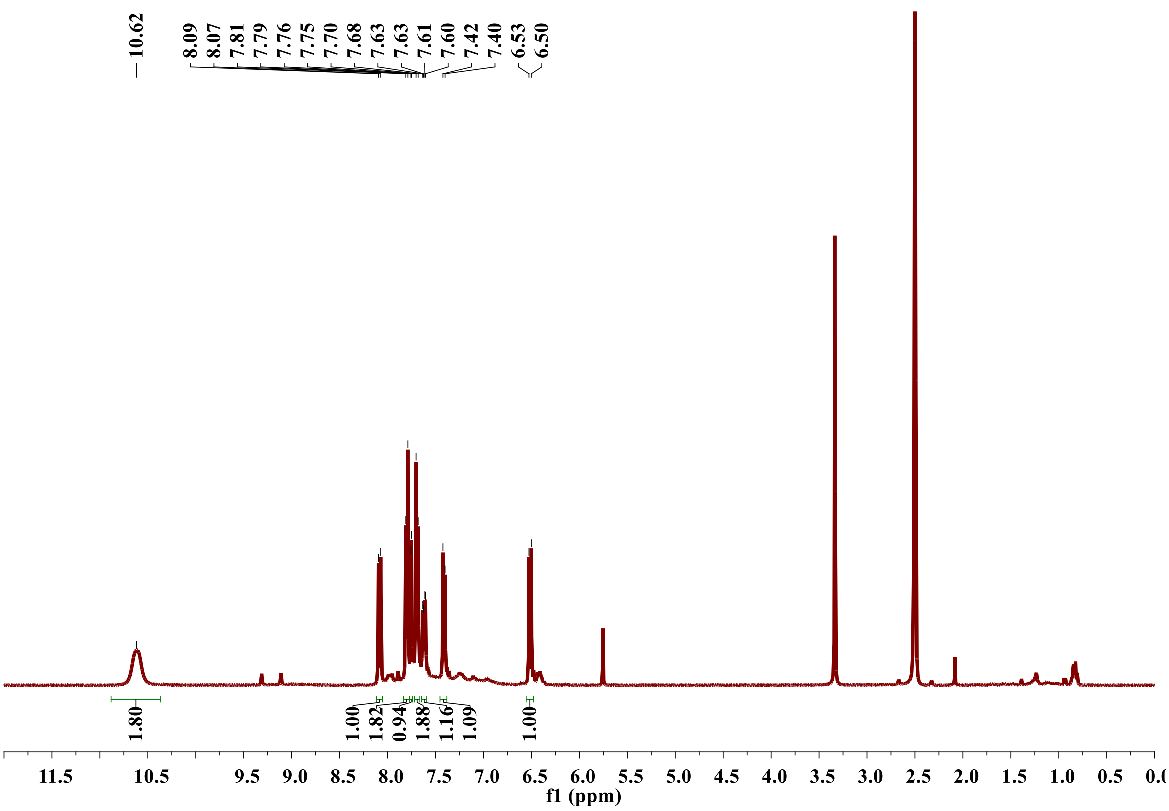

Figure S42. ^{1}H-NMR of compound **3e**.

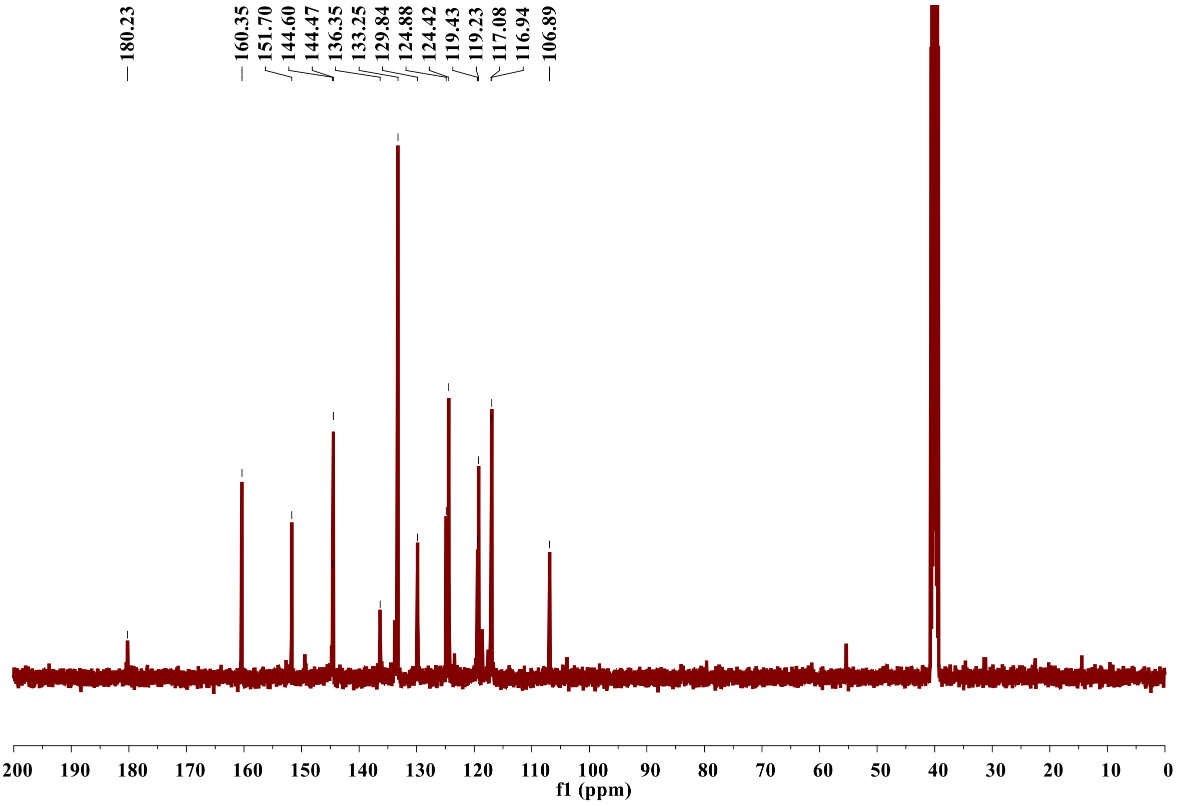

Figure S43. ^{13}C-NMR of compound **3e**.

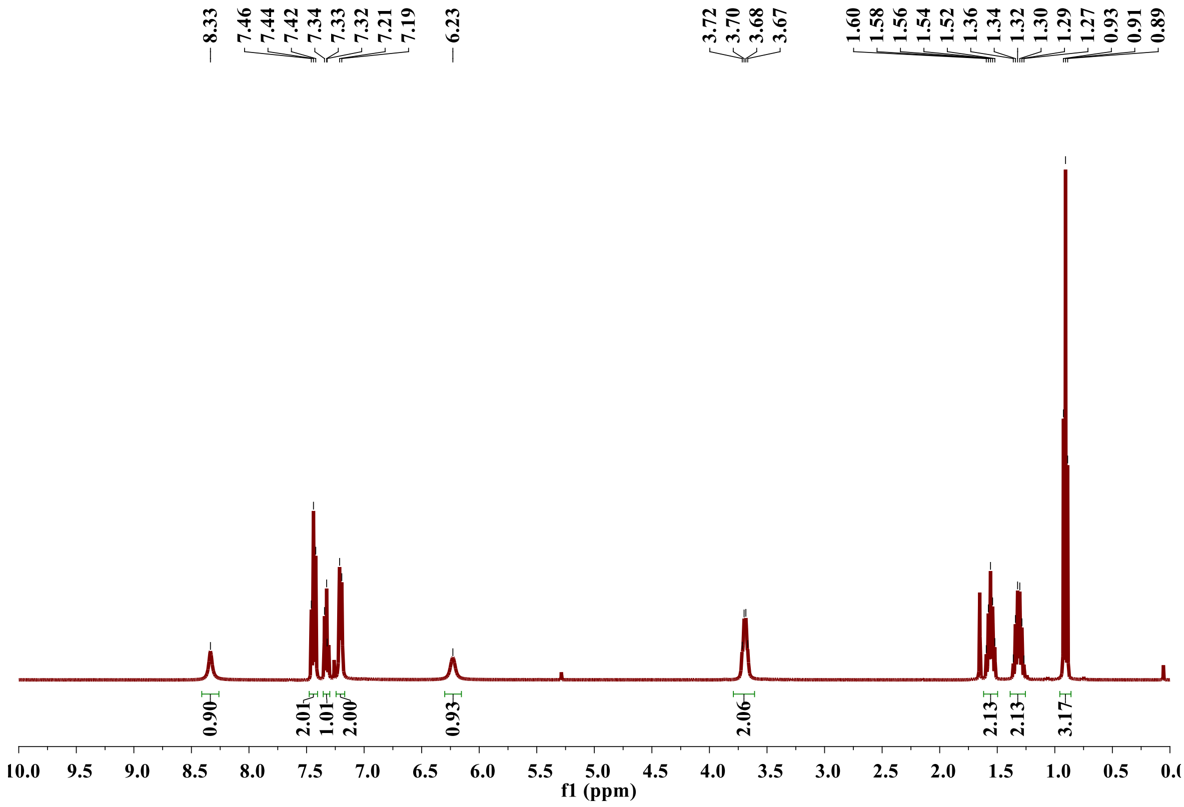

Figure S44. ^{1}H-NMR of compound **4a**.

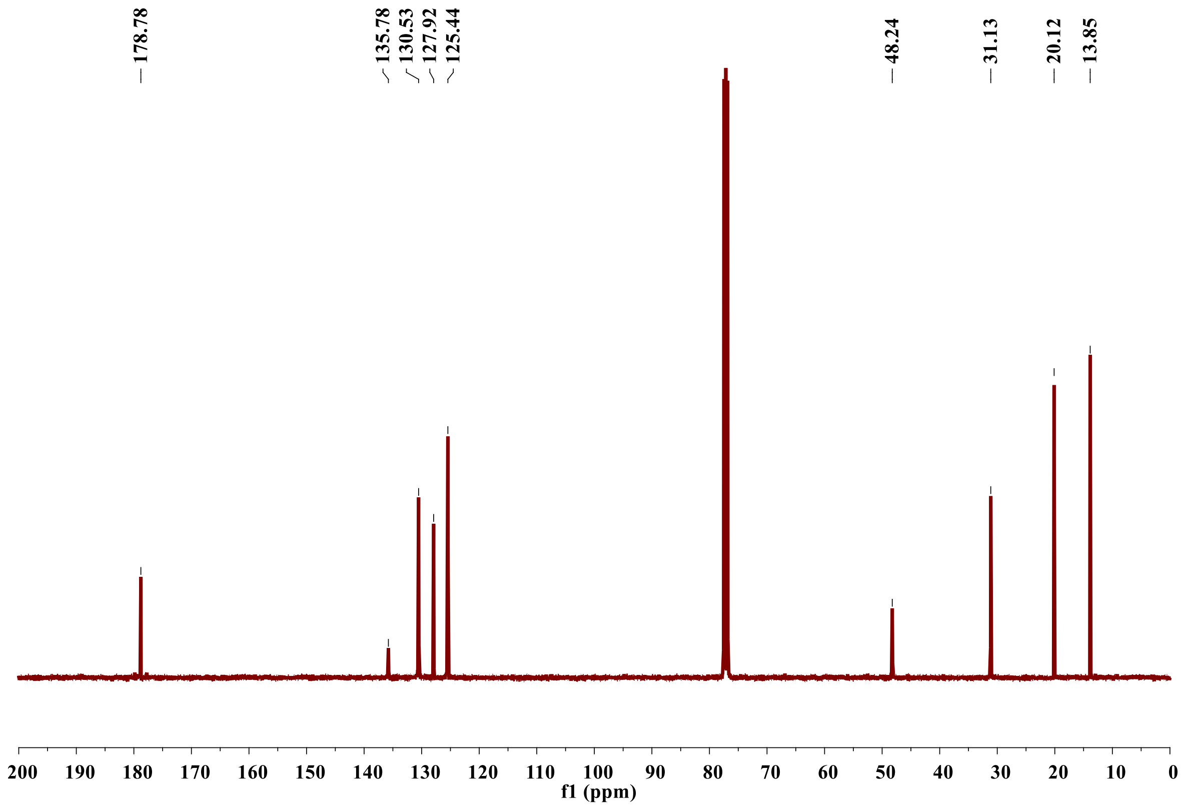

Figure S45. ^{13}C-NMR of compound **4a**.

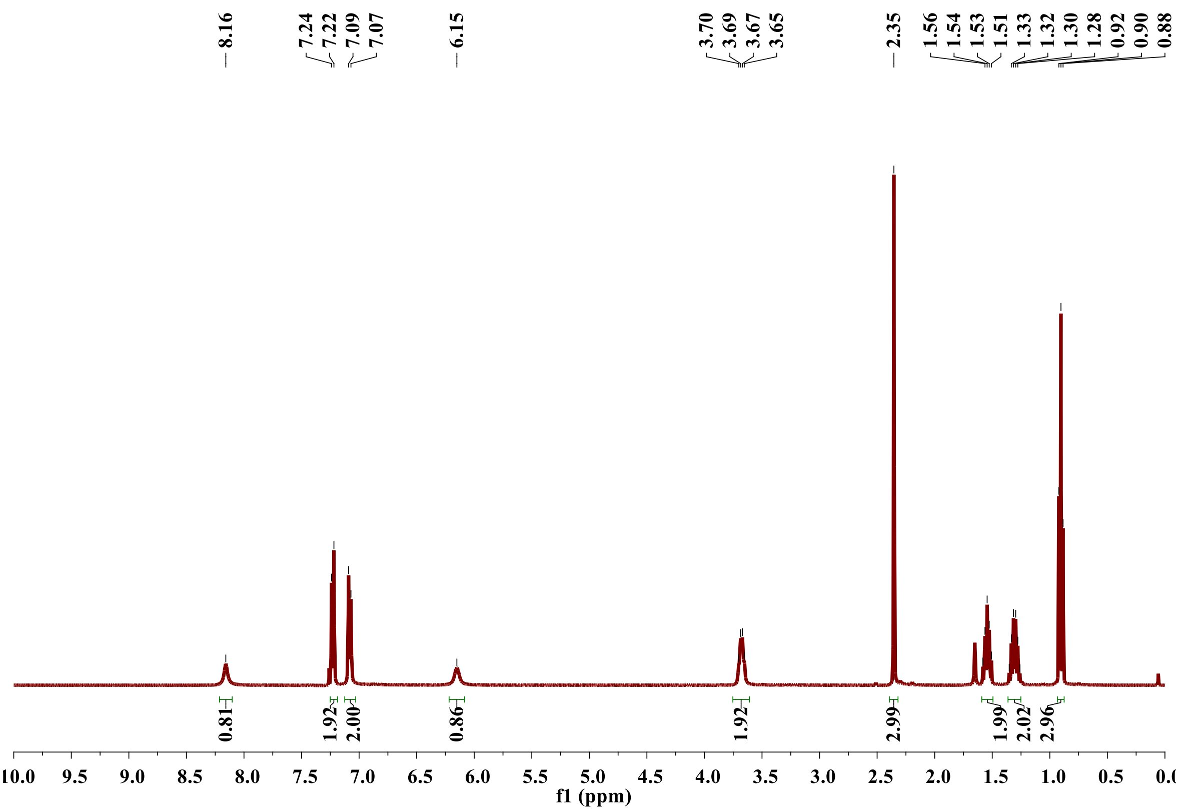

Figure S46. ^{1}H-NMR of compound **4b**.

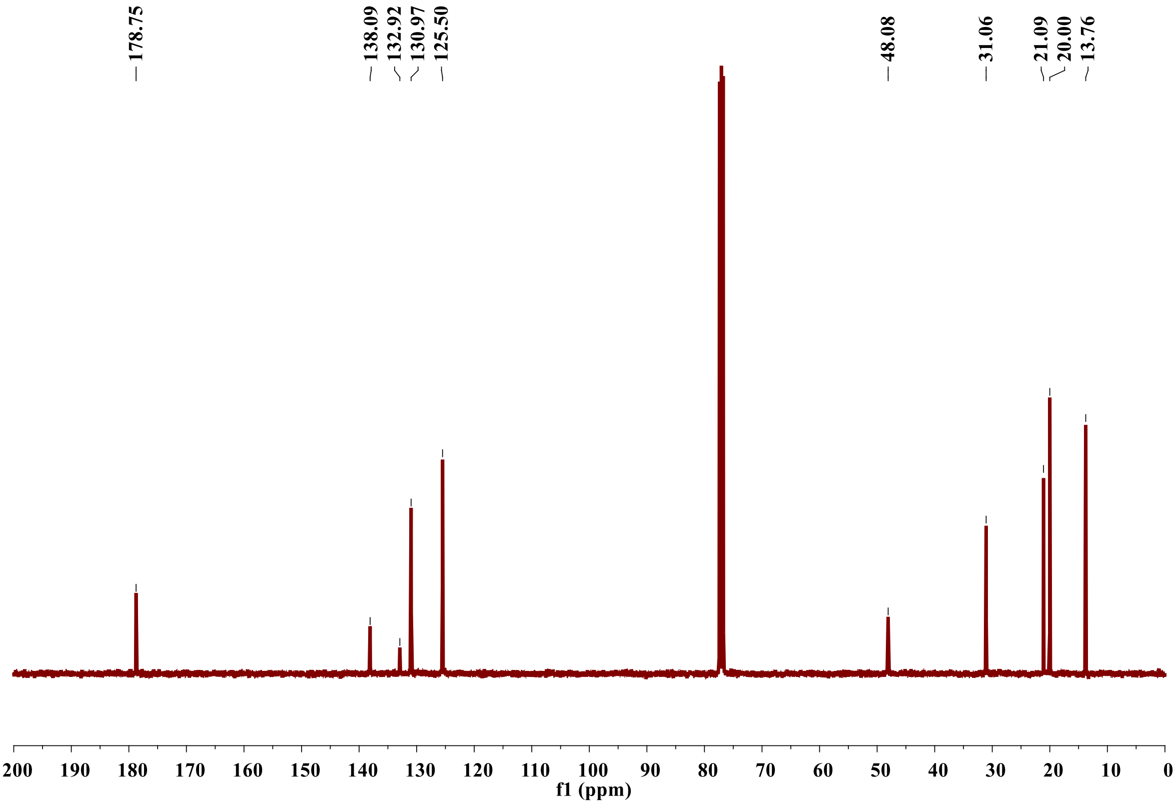

Figure S47. ^{13}C-NMR of compound **4b**.

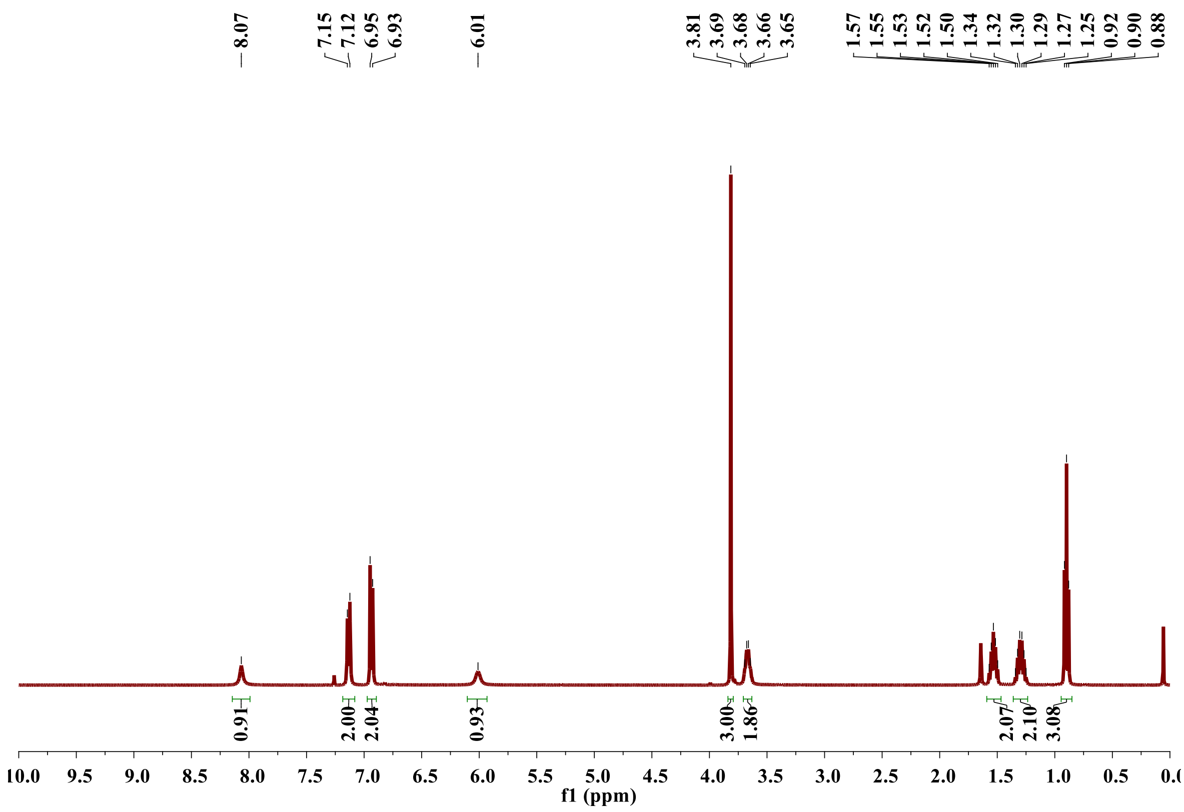

Figure S48. ^{1}H-NMR of compound **4c**.

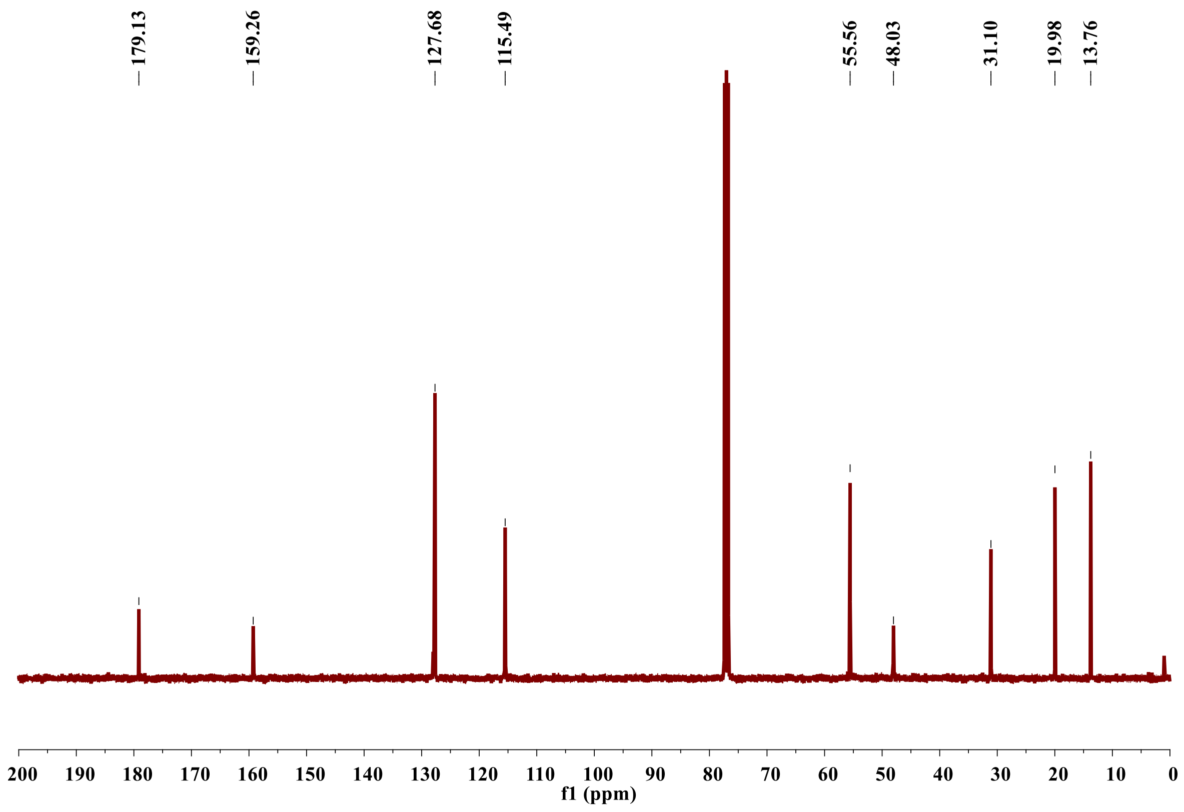

Figure S49. ^{13}C-NMR of compound **4c**.

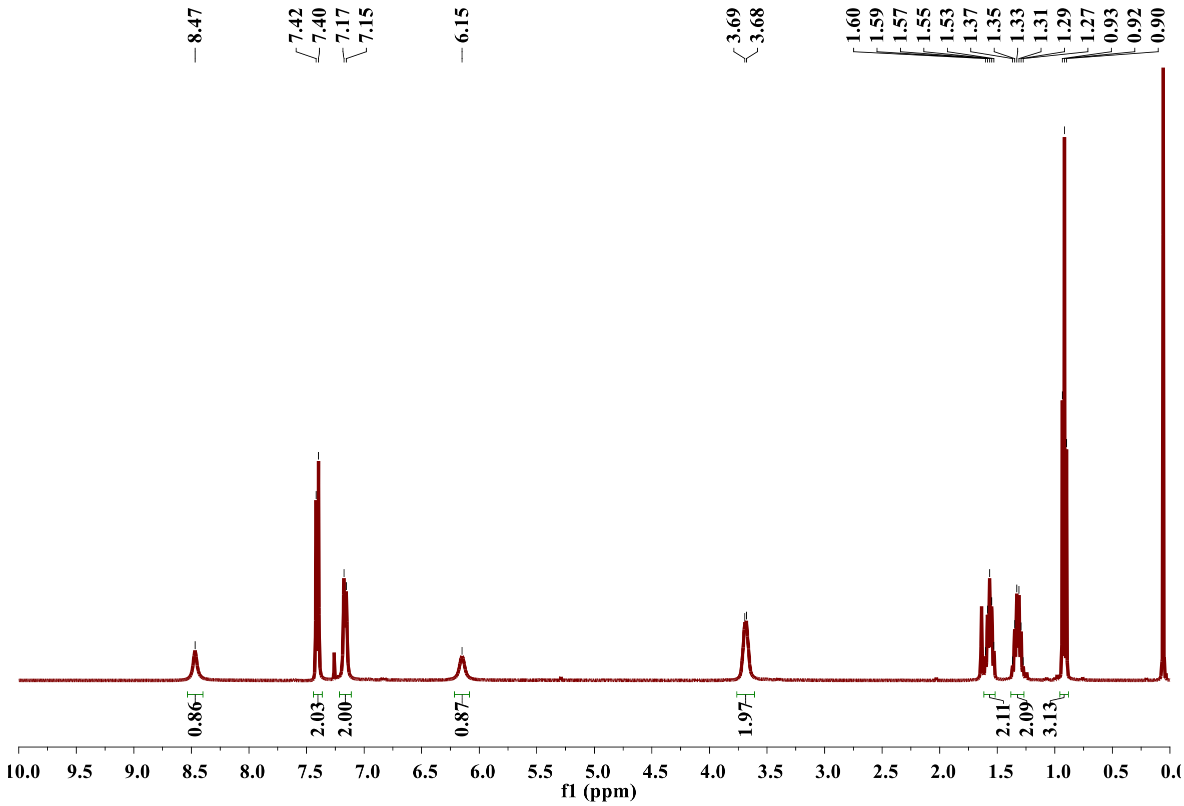

Figure S50. ^{1}H-NMR of compound **4d**.

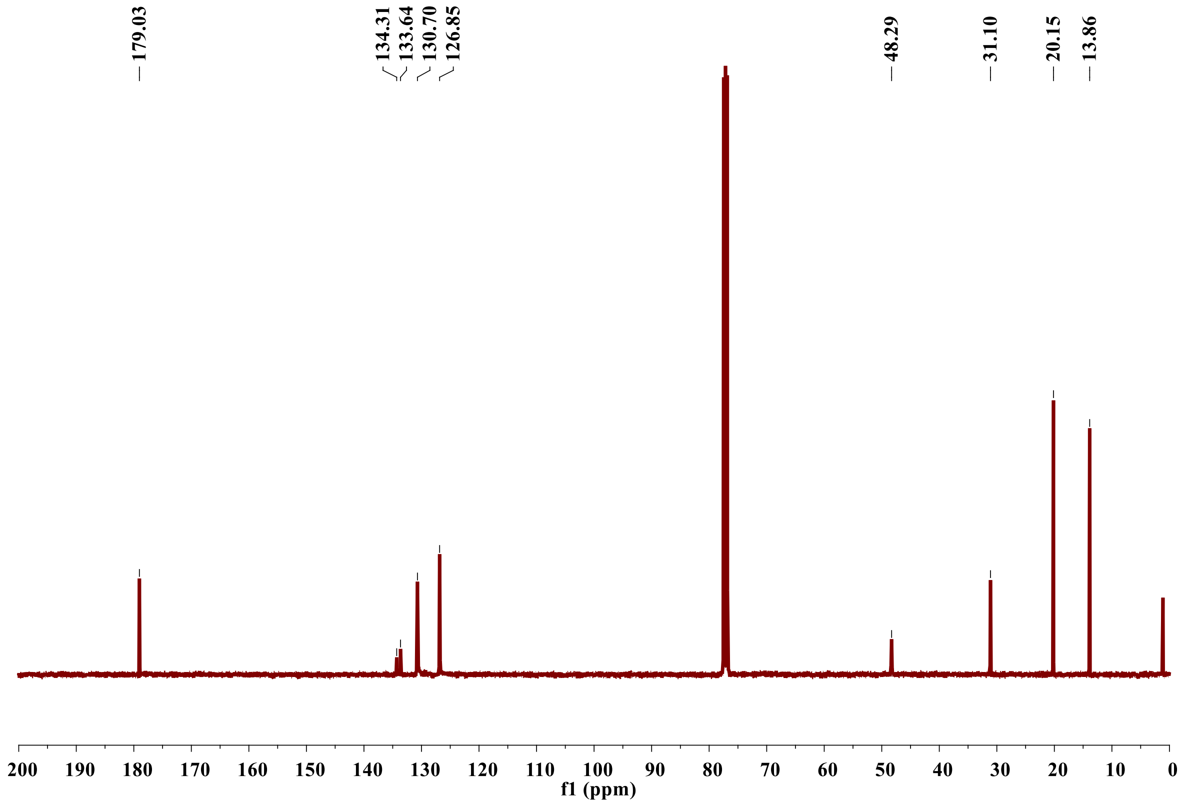

Figure S51. ^{13}C-NMR of compound **4d**.

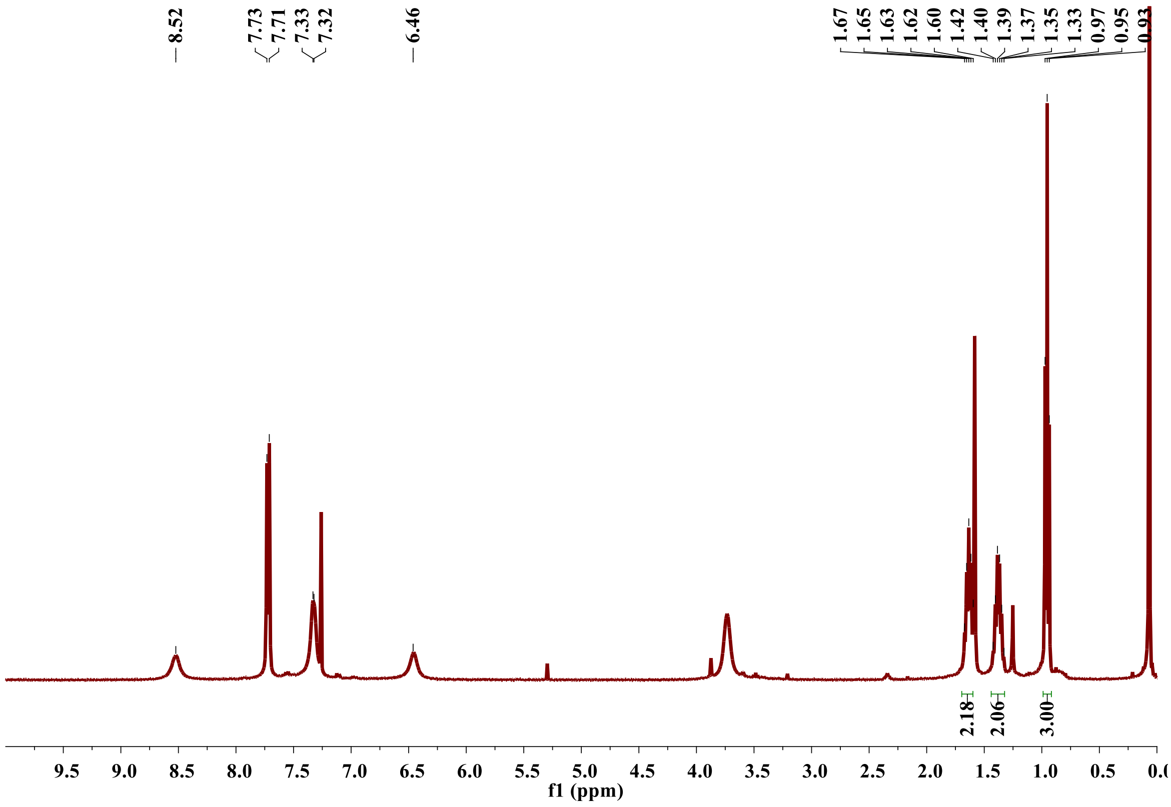

Figure S52. ¹H-NMR of compound **4e**.

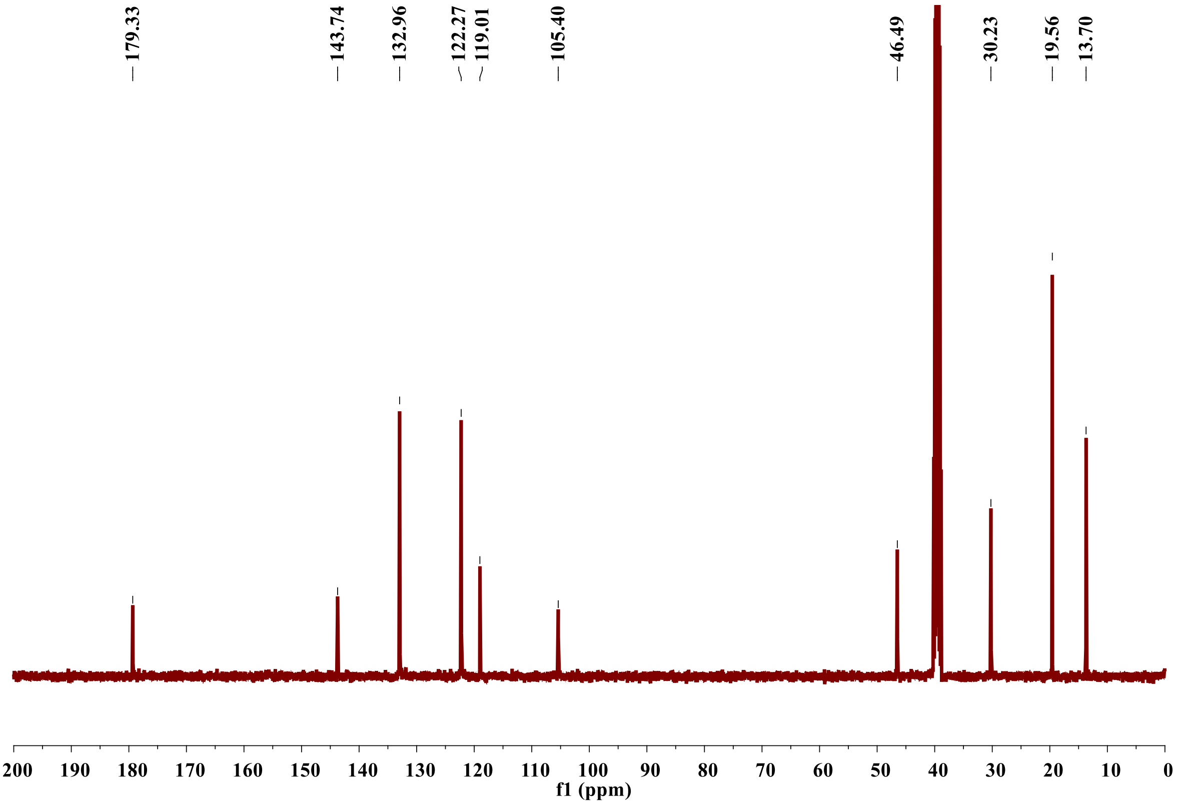

Figure S53. ¹³C-NMR of compound **4e**.

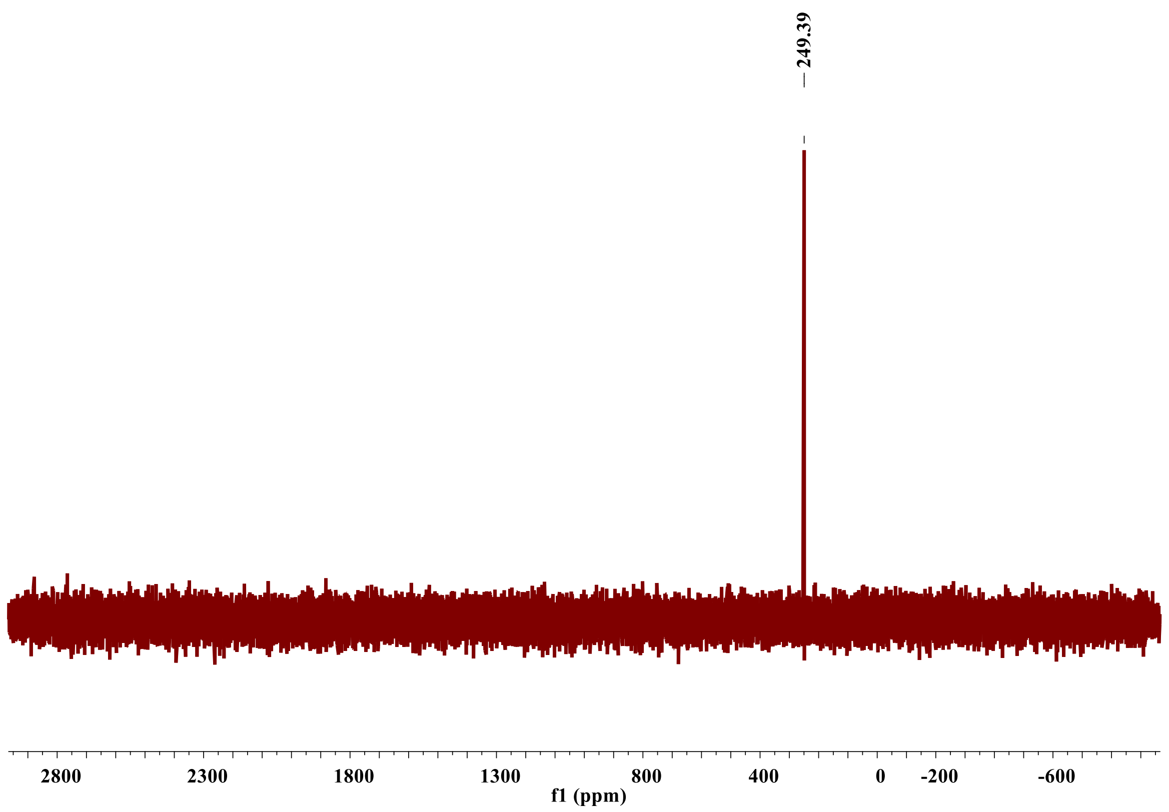

Figure S54. ^{77}Se-NMR of compound **4e**.

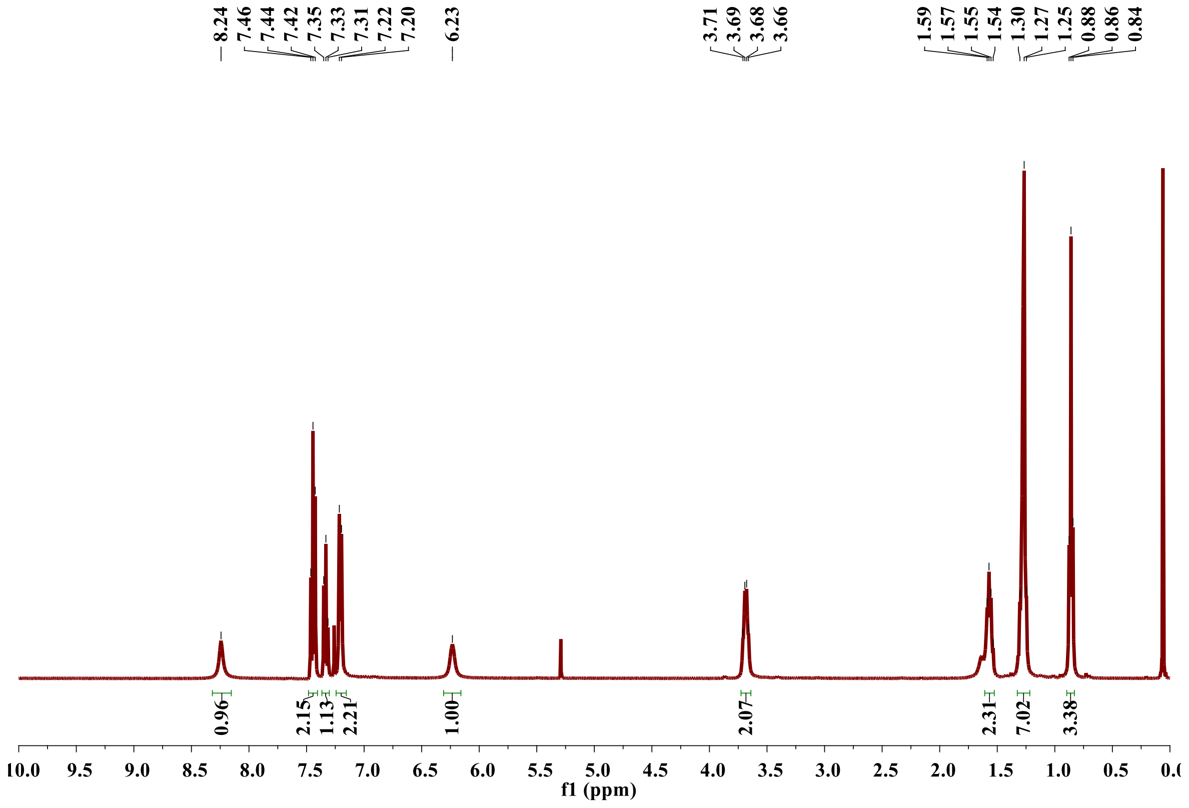

Figure S55. ^{1}H-NMR of compound **5a**.

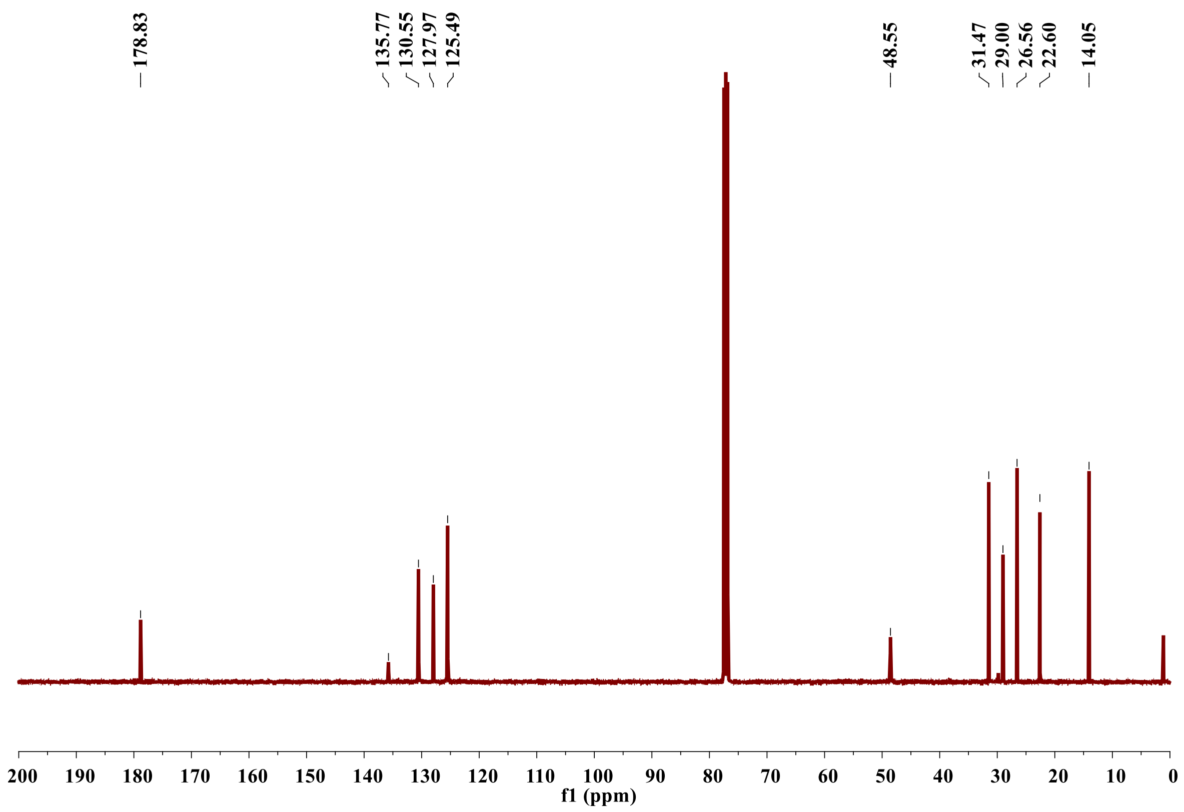

Figure S56. ^{13}C-NMR of compound **5a**.

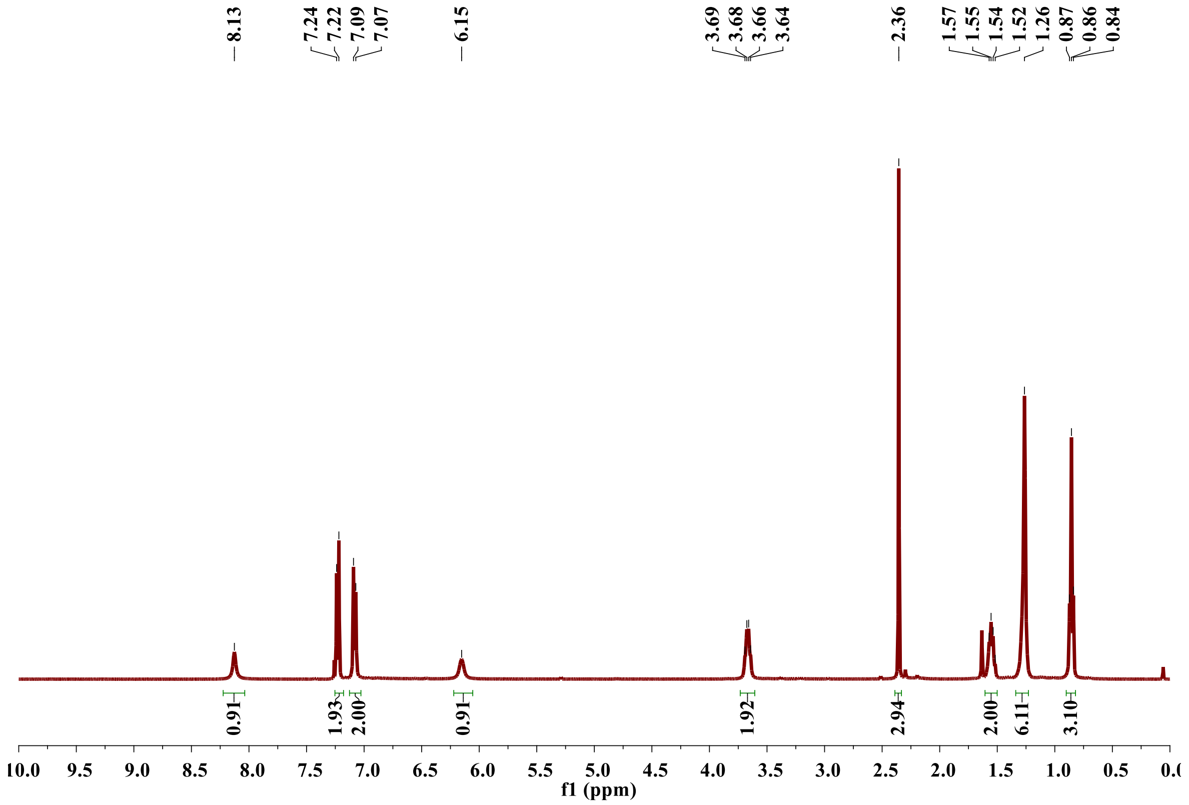

Figure S57. ^{1}H-NMR of compound **5b**.

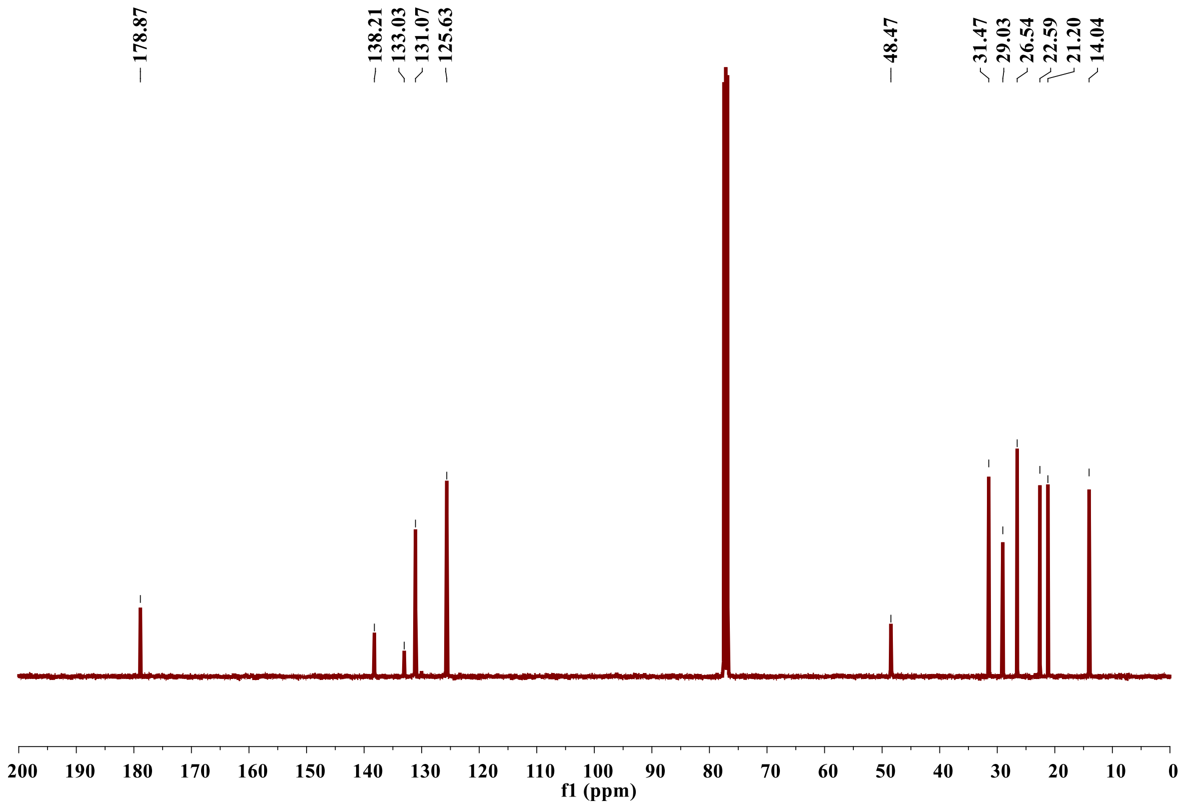

Figure S58. ^{13}C-NMR of compound **5b**.

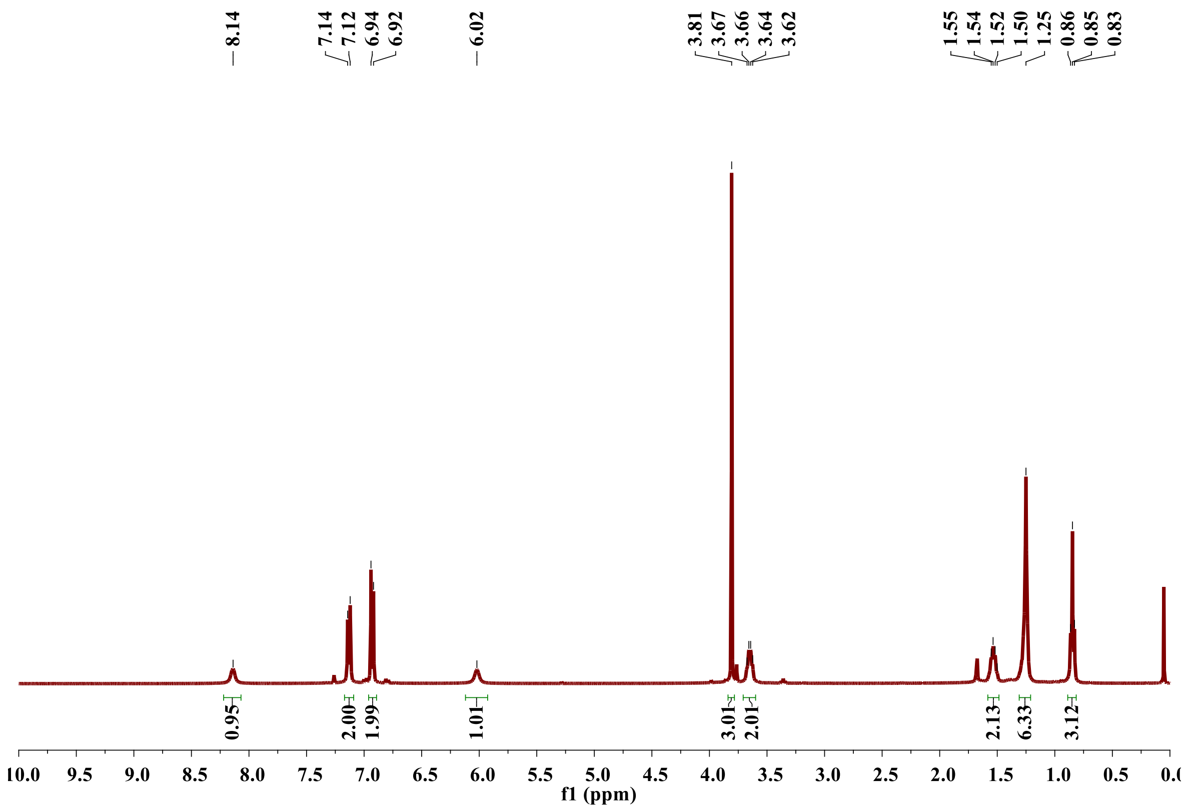

Figure S59. ^{1}H-NMR of compound **5c**.

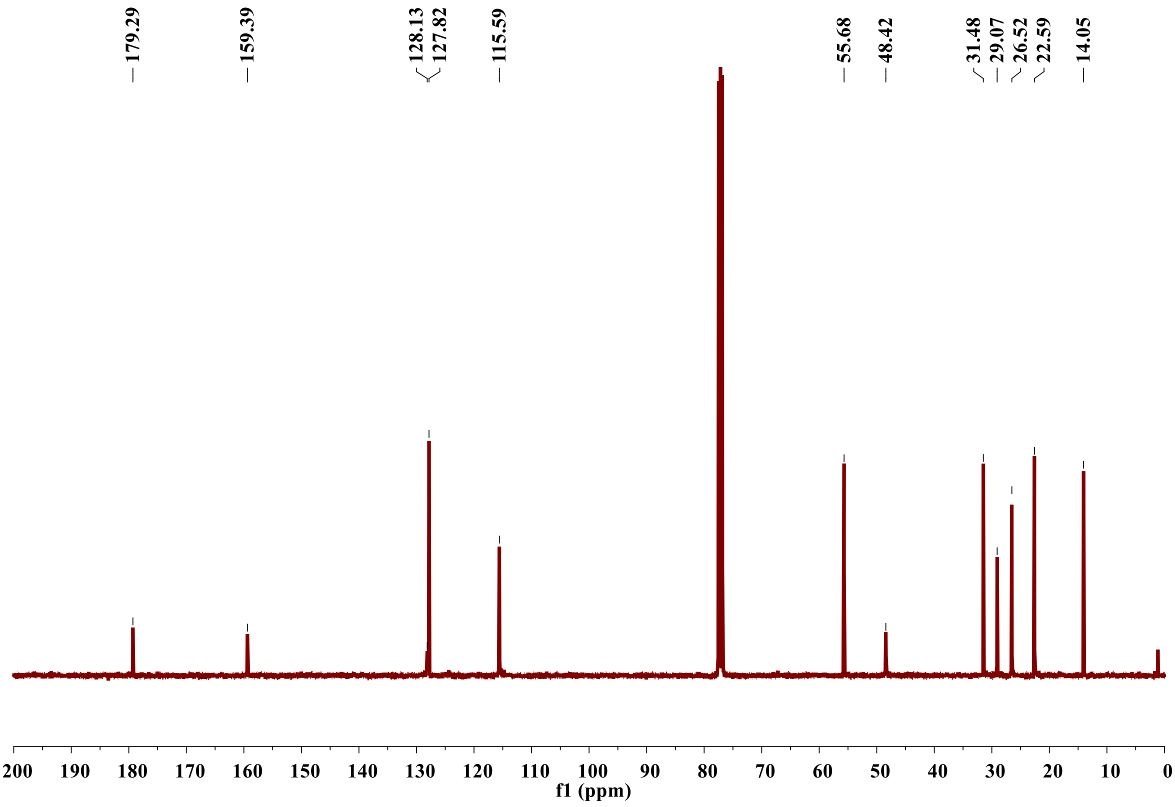

Figure S60. ^{13}C-NMR of compound **5c**.

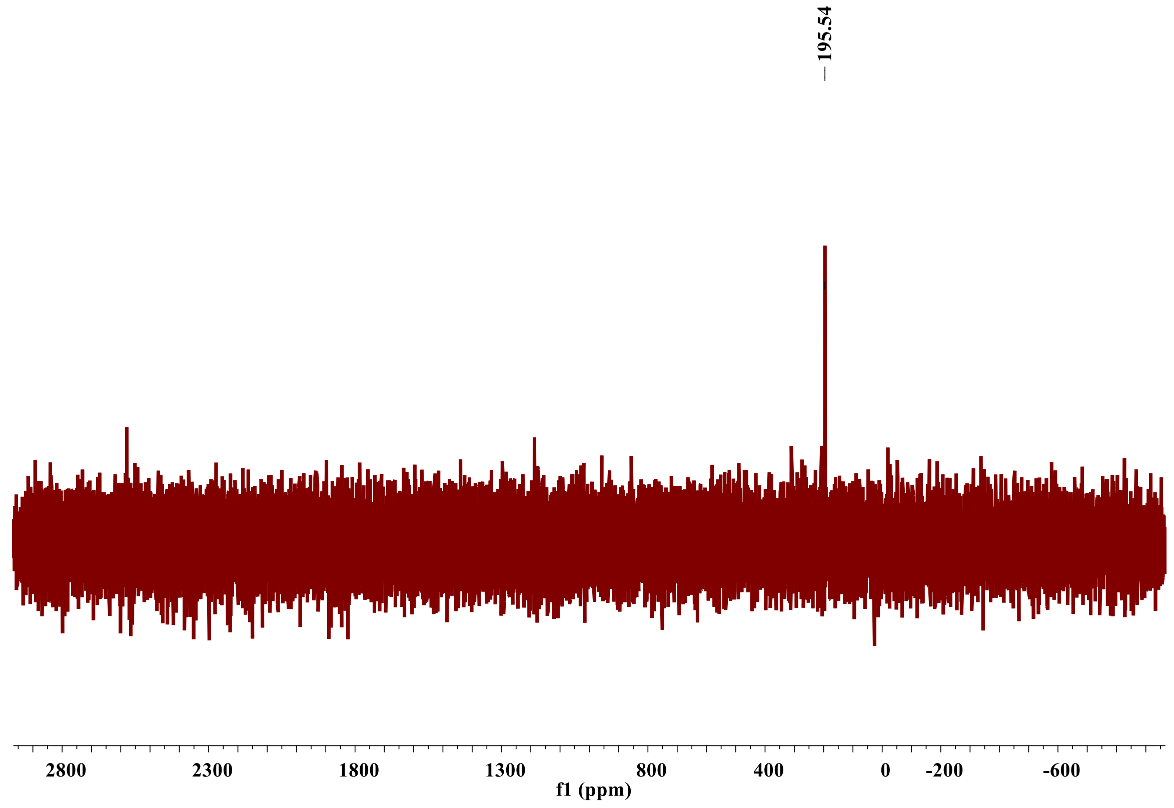

Figure S61. ^{77}Se-NMR of compound **5c**.

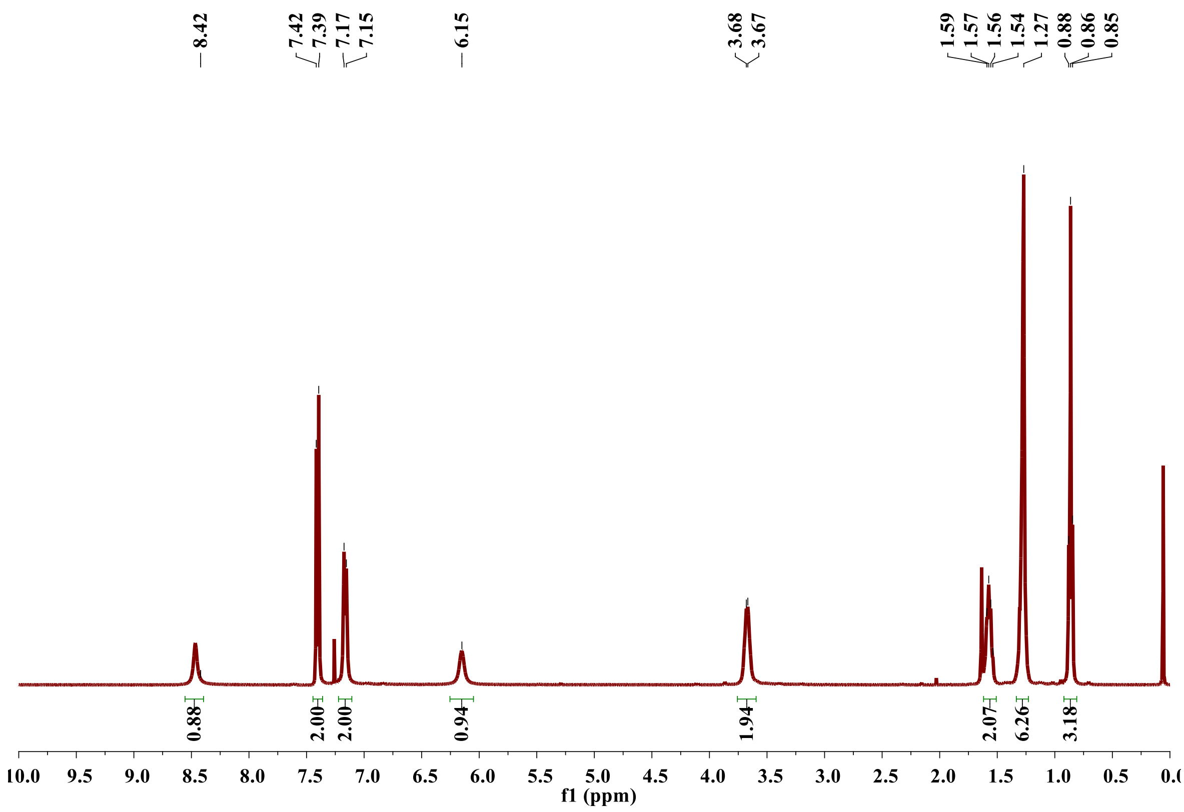

Figure S62. ^{1}H-NMR of compound **5d**.

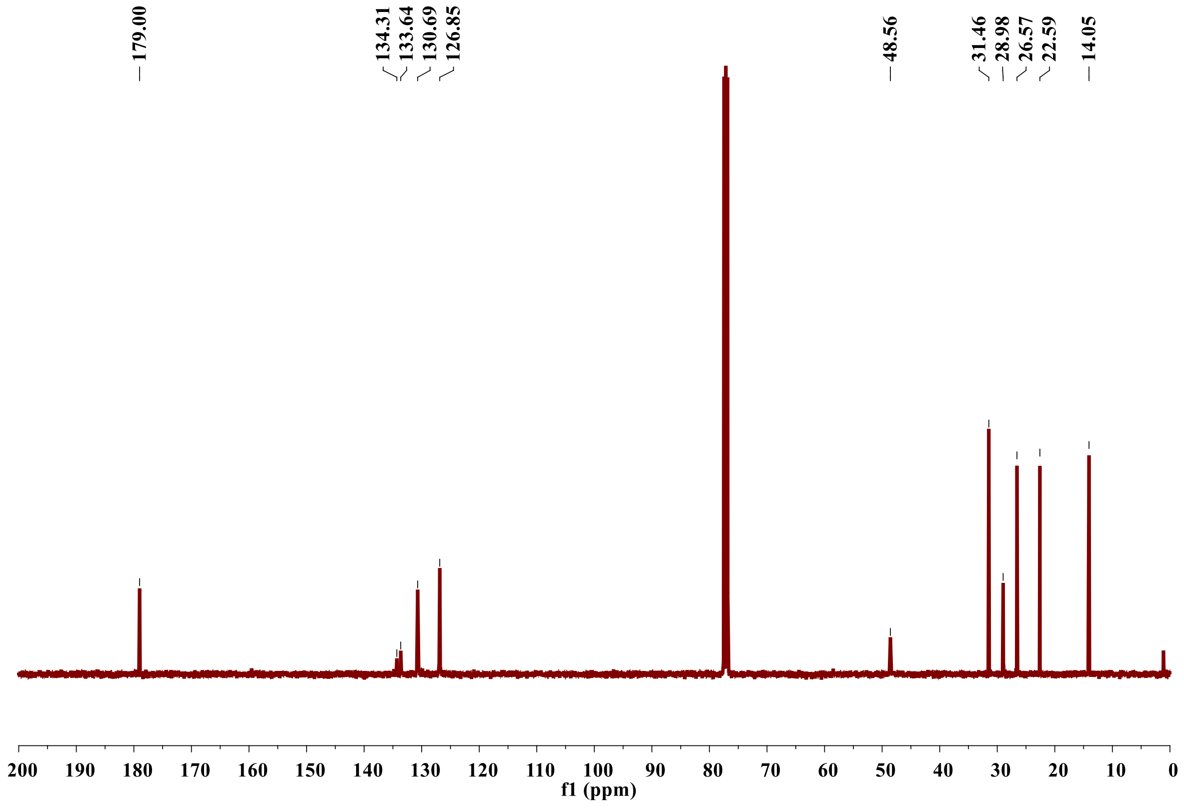

Figure S63. ^{13}C-NMR of compound **5d**.

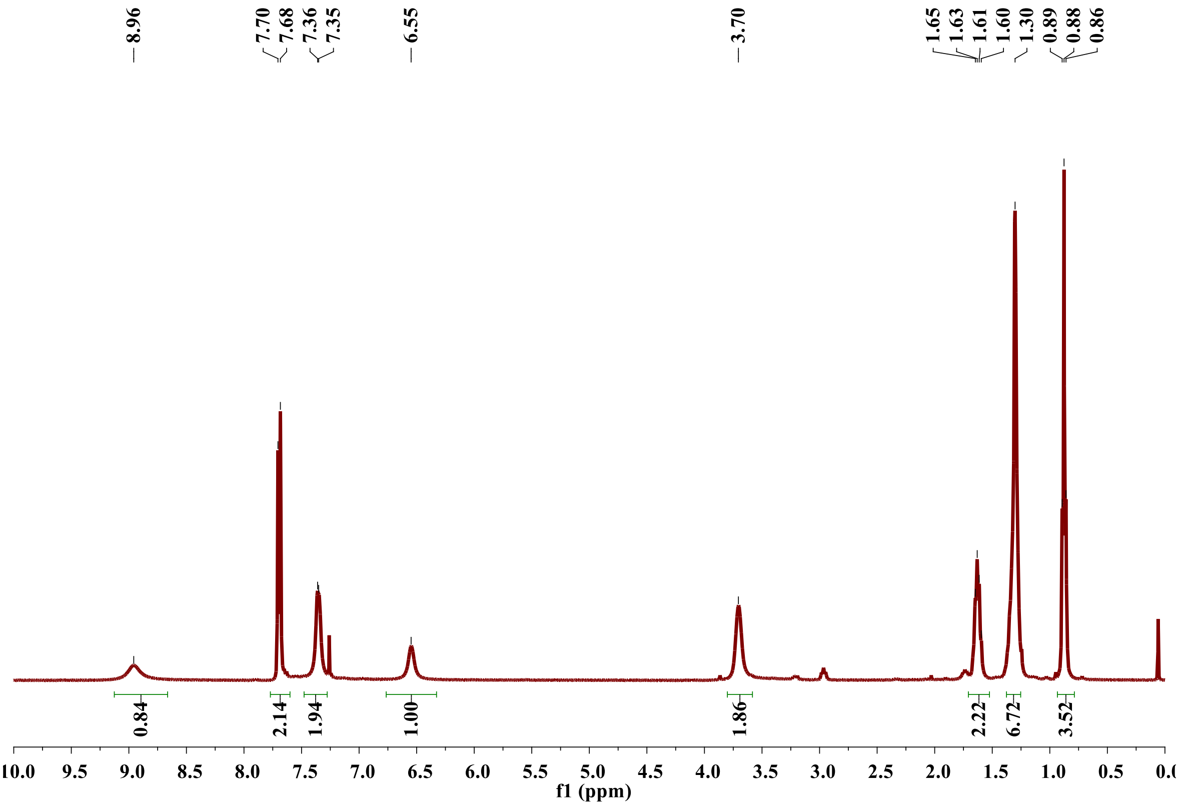

Figure S64. ^{1}H-NMR of compound **5e**.

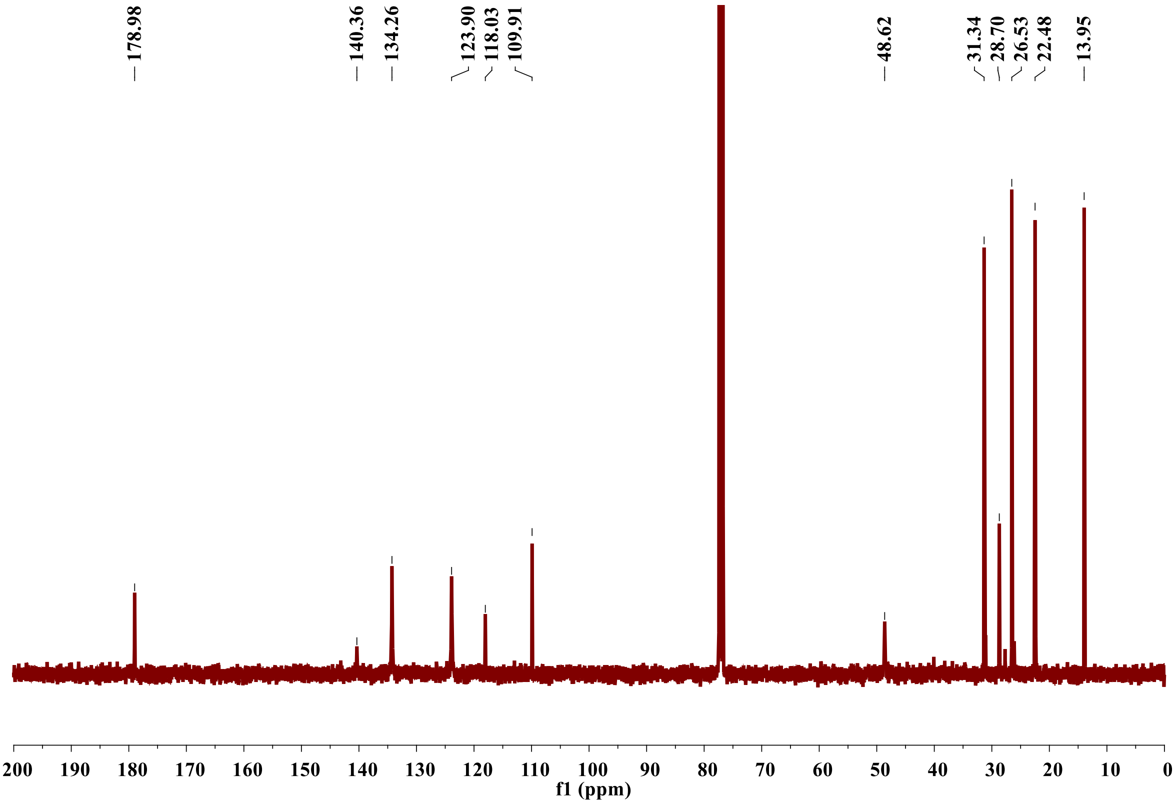

Figure S65. ^{13}C-NMR of compound **5e**.

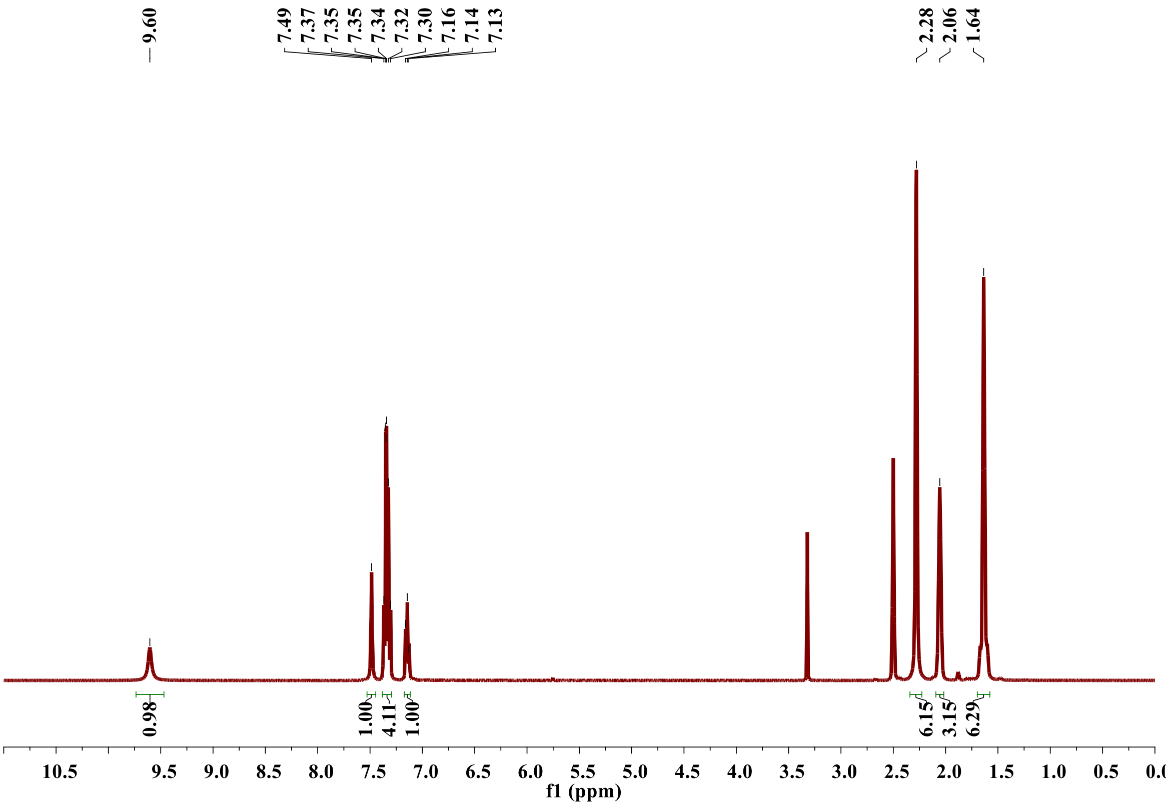

Figure S66. ¹H-NMR of compound **6a**.

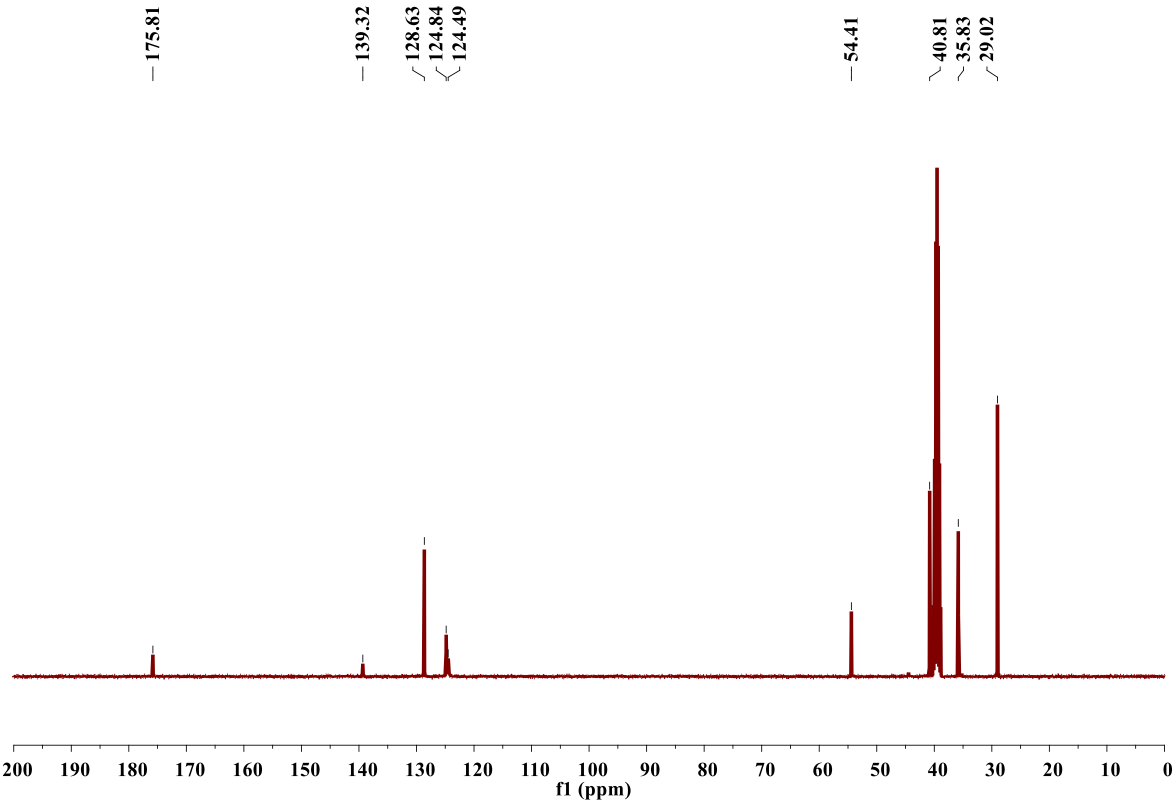

Figure S67. ¹³C-NMR of compound **6a**.

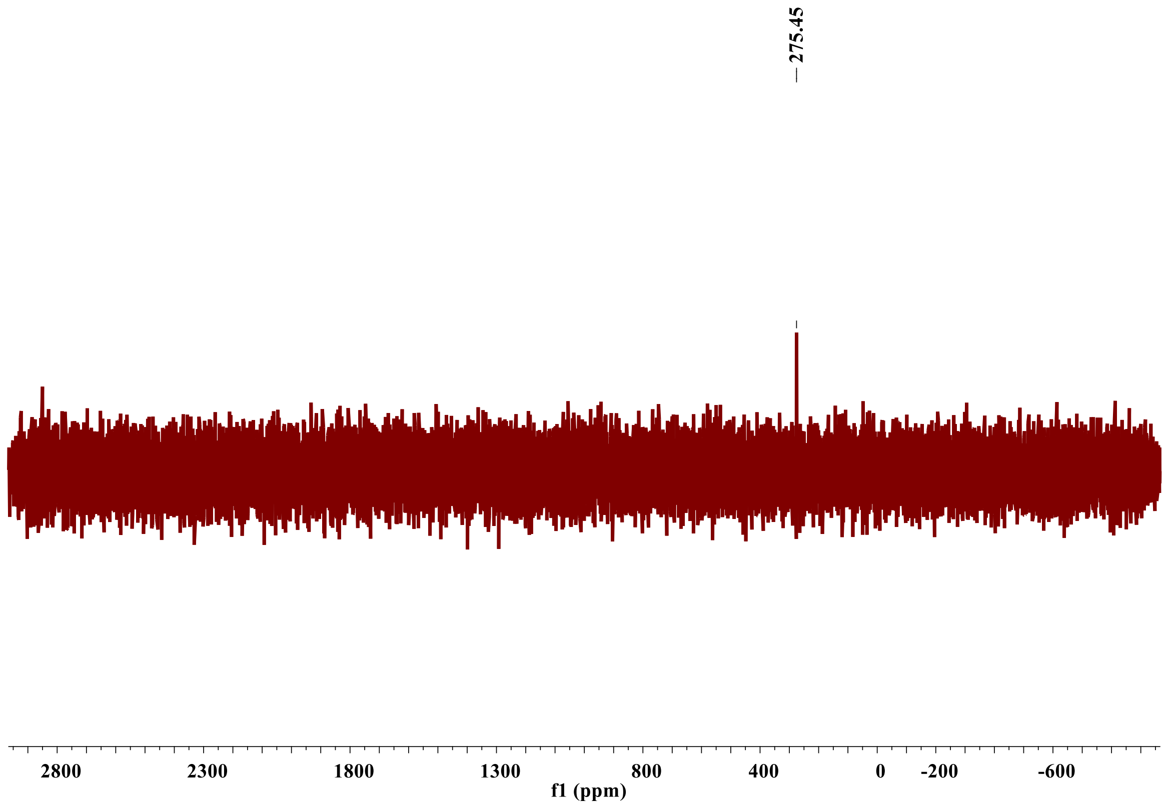

Figure S68. ^{77}Se-NMR of compound **6a**.

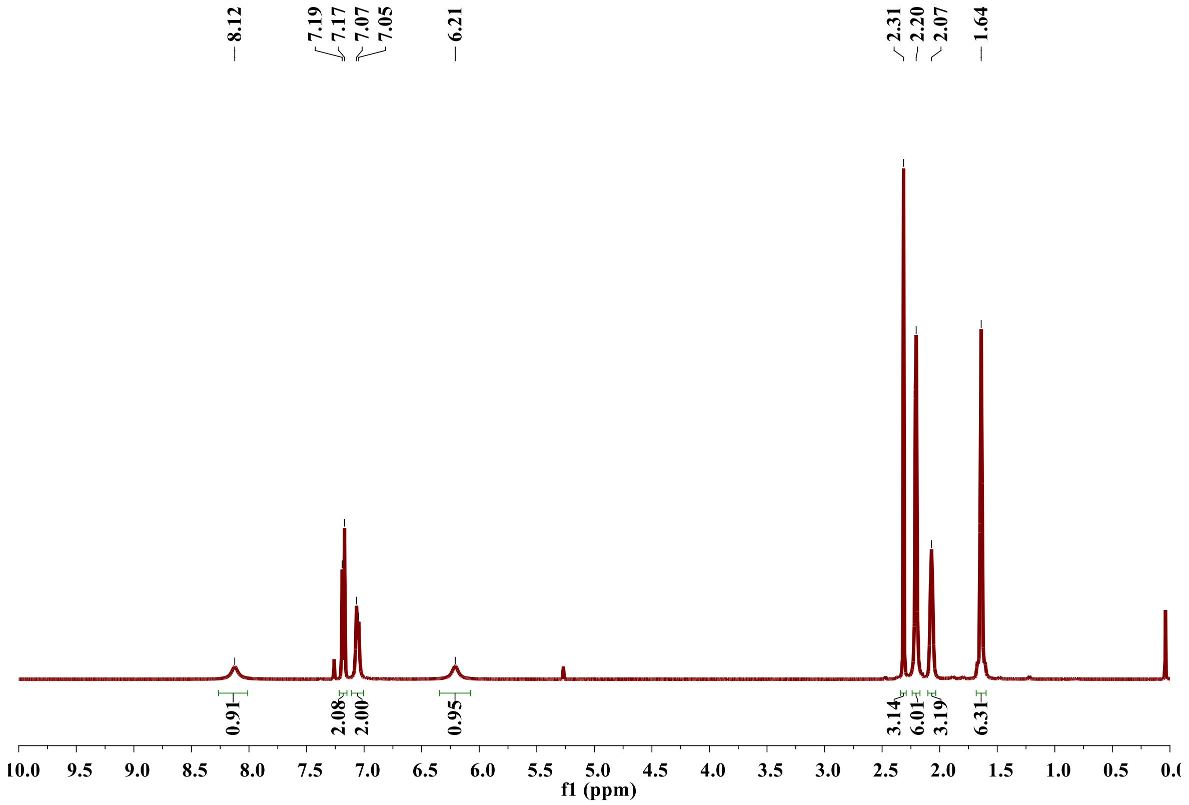

Figure S69. ^{1}H-NMR of compound **6b**.

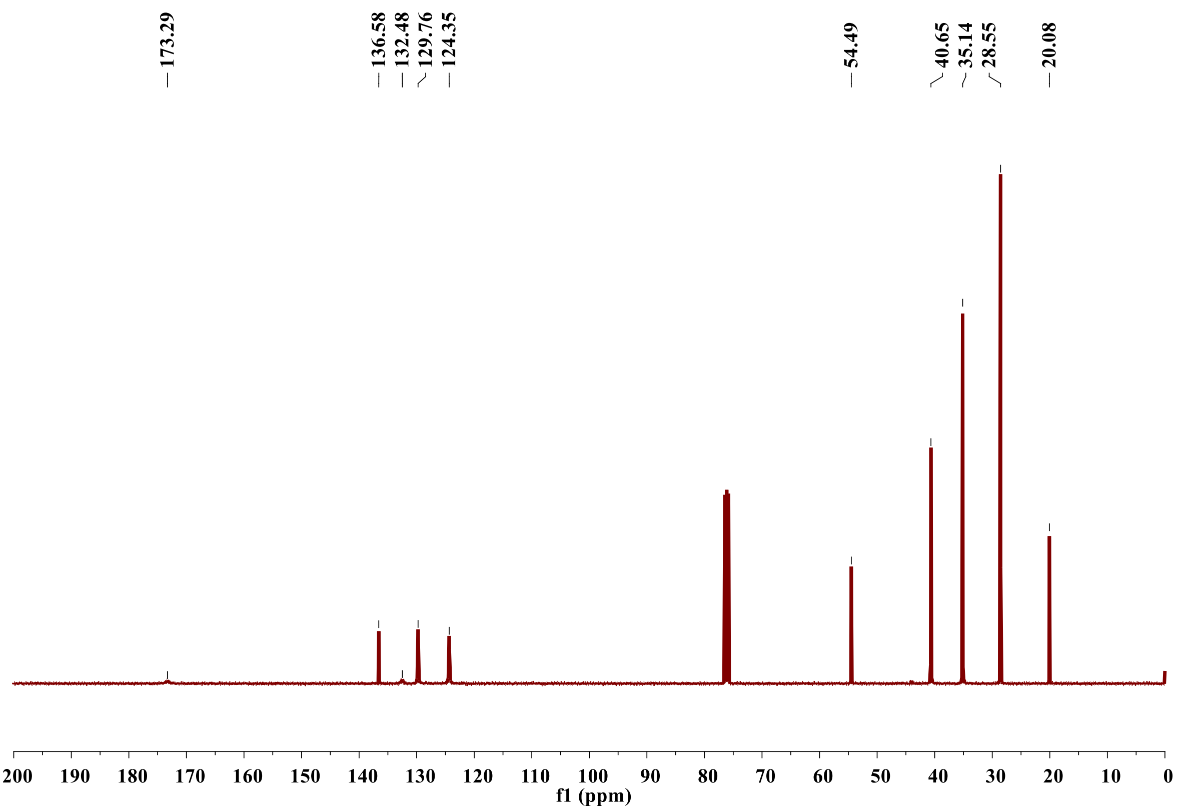

Figure S70. ^{13}C-NMR of compound **6b**.

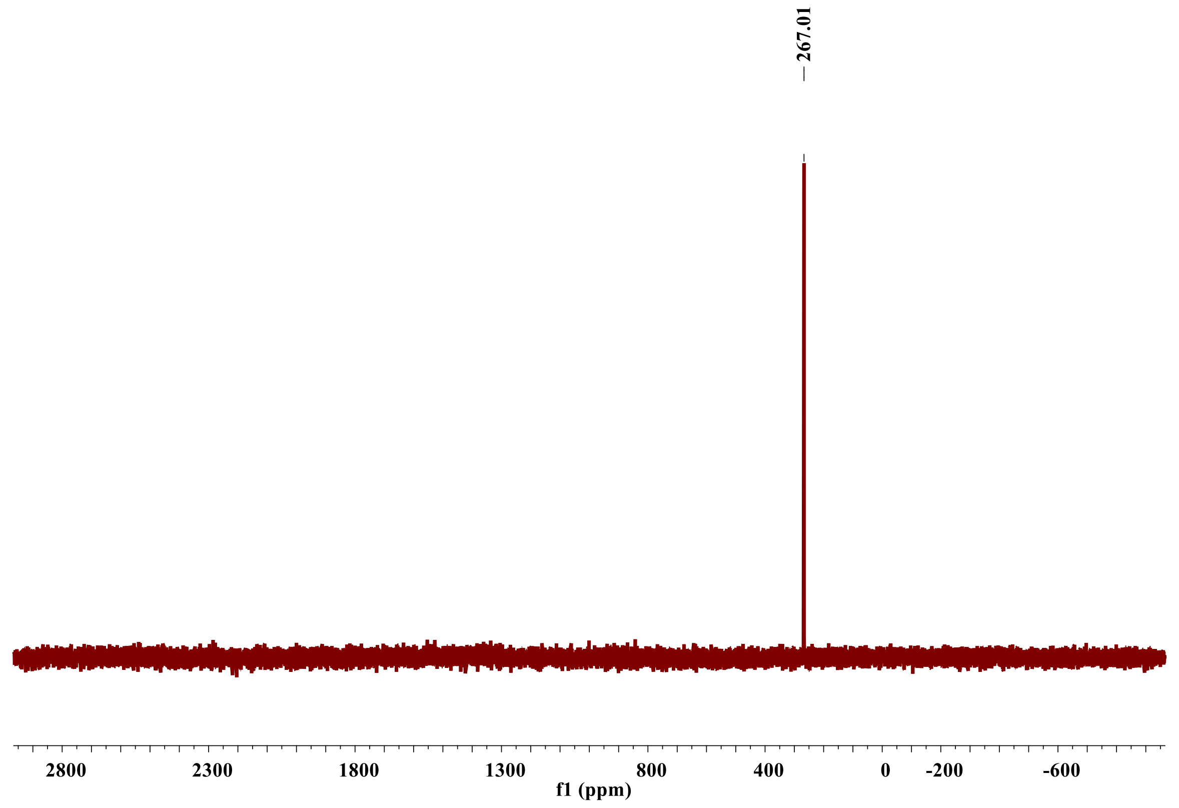

Figure S71. ^{77}Se-NMR of compound **6b**.

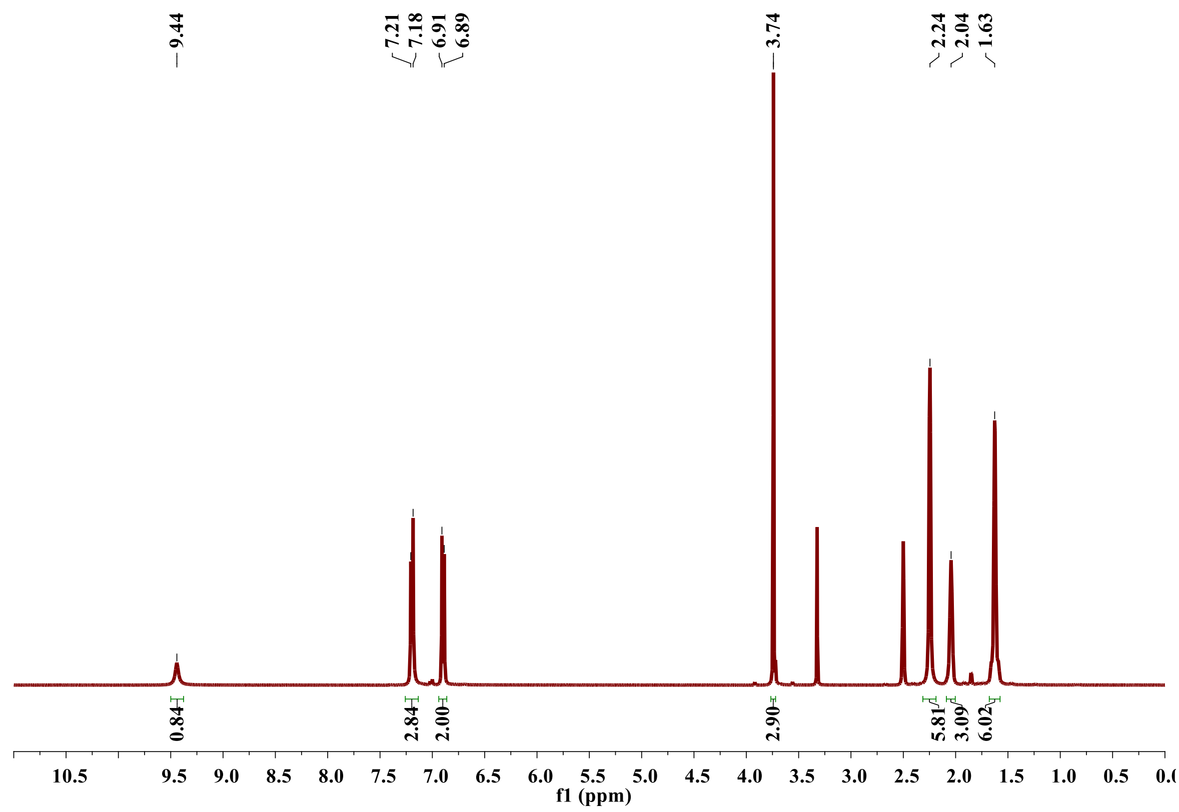

Figure S72. ^{1}H-NMR of compound **6c**.

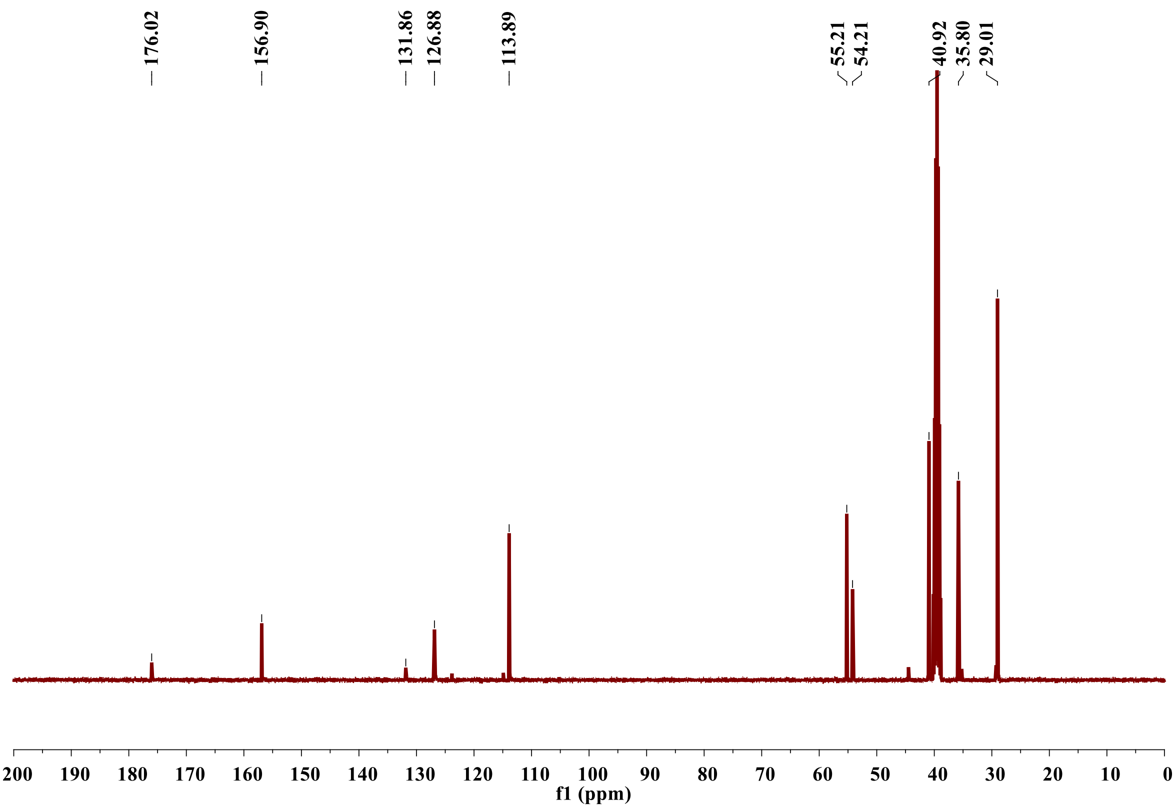

Figure S73. ^{13}C-NMR of compound **6c**.

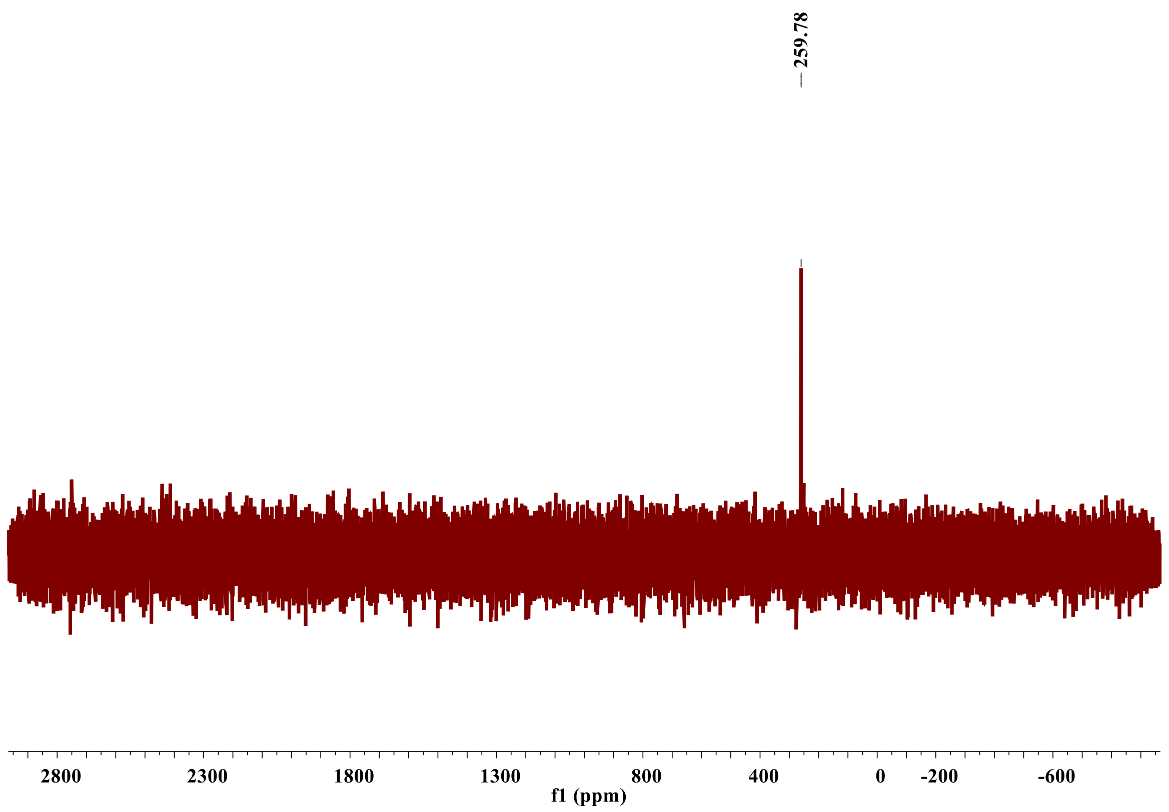

Figure S74. ^{77}Se-NMR of compound **6c**.

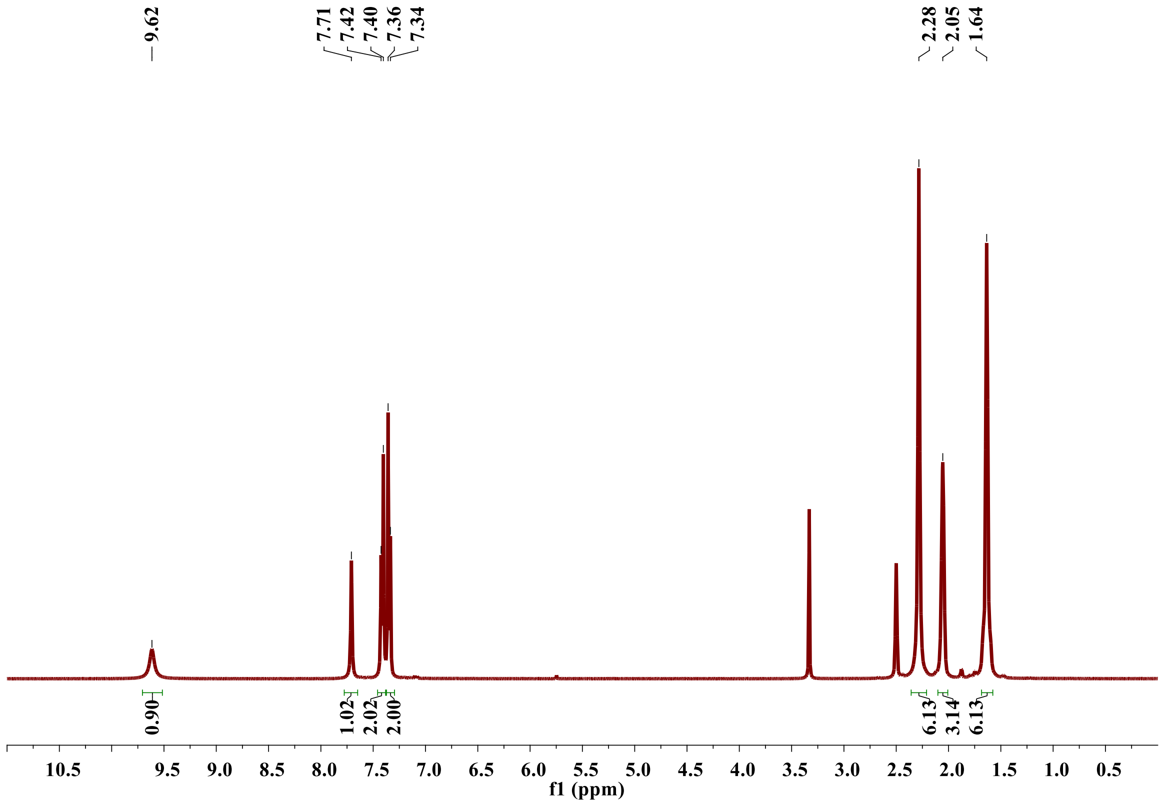

Figure S75. ^{1}H-NMR of compound **6d**.

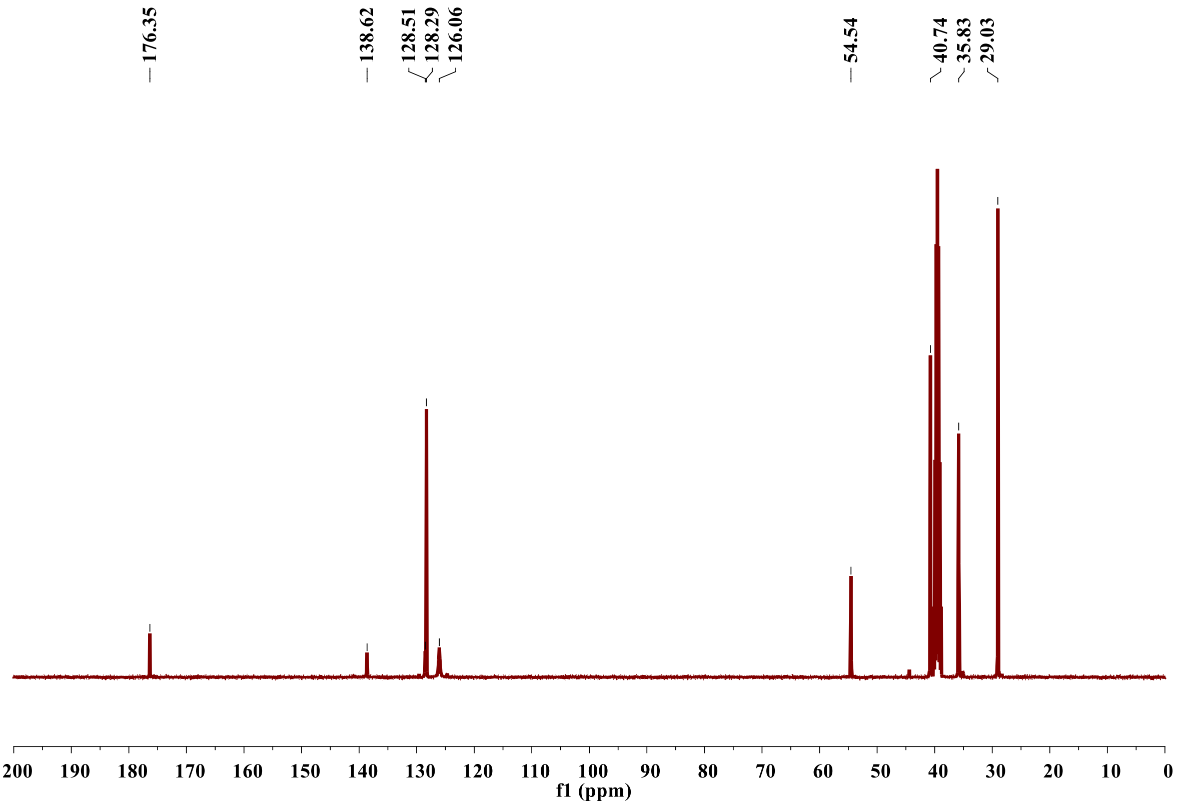

Figure S76. ^{13}C-NMR of compound **6d**.

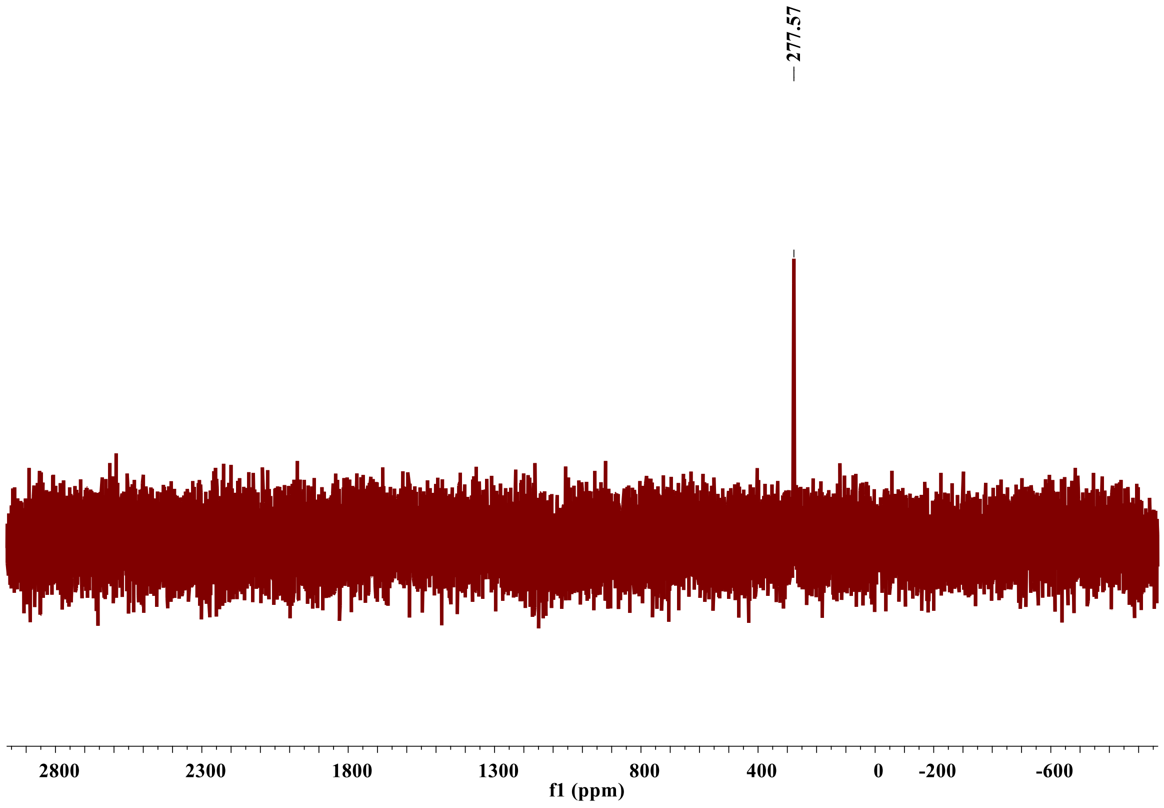

Figure S77. ^{77}Se-NMR of compound **6d**.

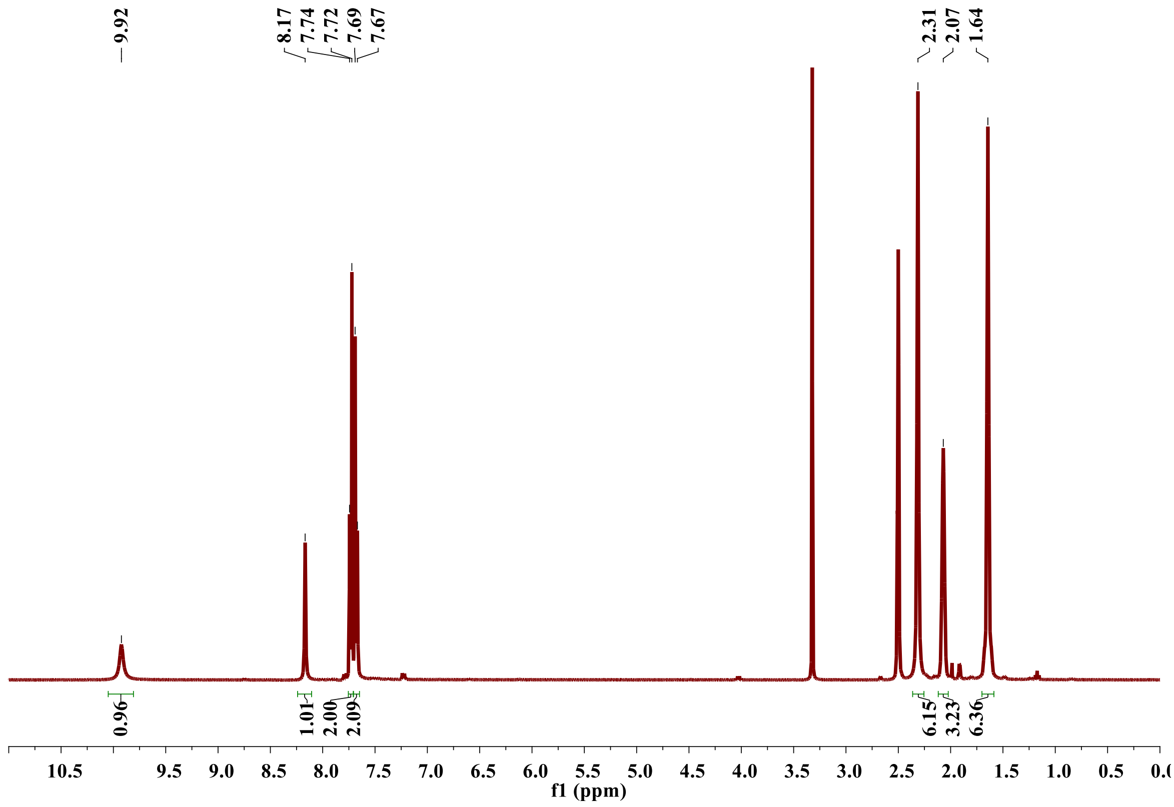

Figure S78. ¹H-NMR of compound **6e**.

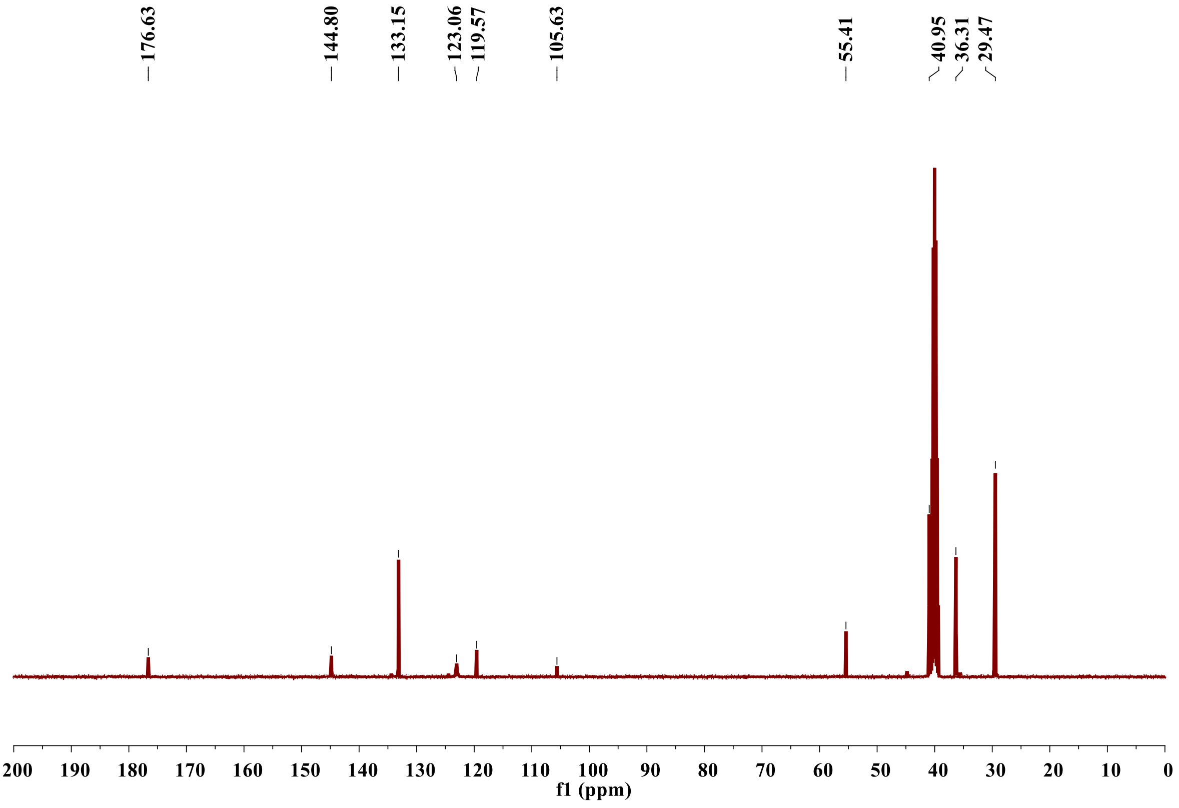

Figure S79. ¹³C-NMR of compound **6e**.

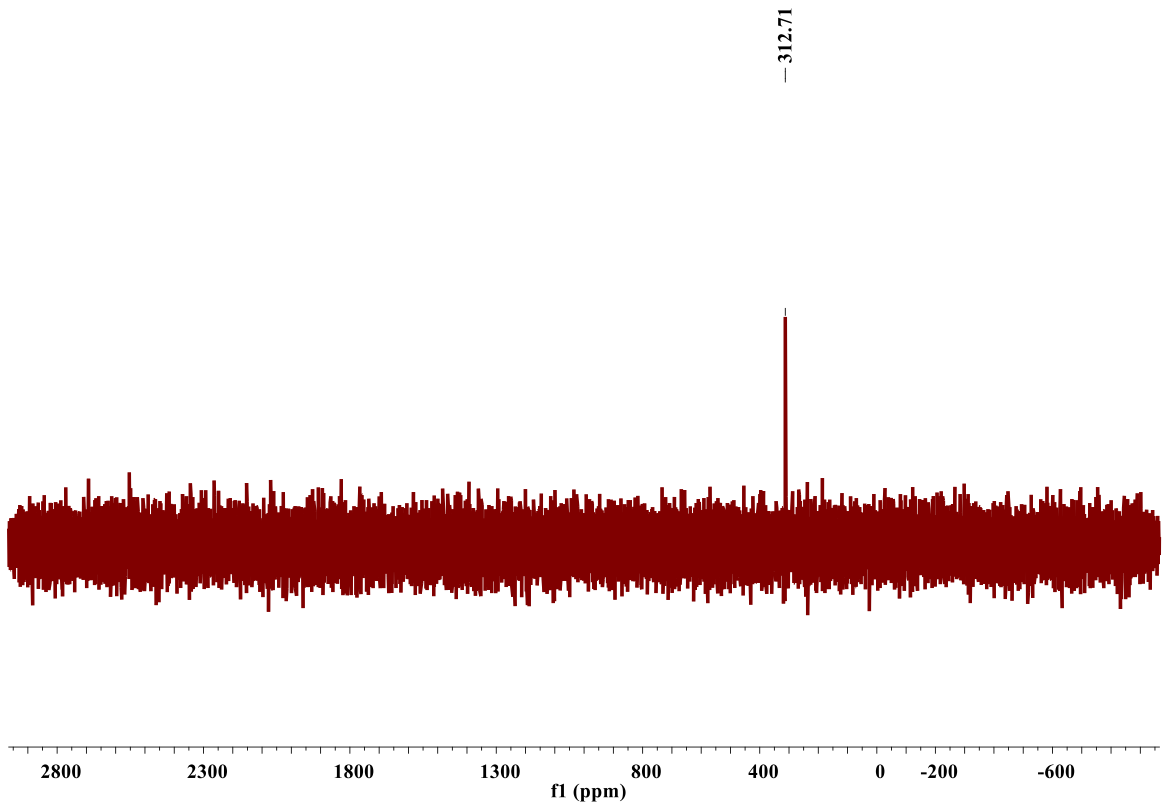

Figure S80. ^{77}Se-NMR of compound **6e**.

Supplementary data for DPPH and ABTS assays.

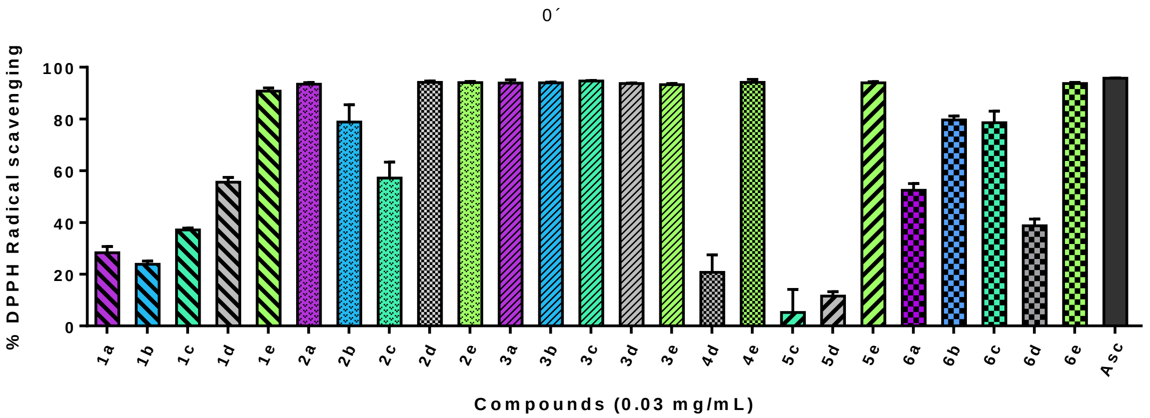

Figure S81. DPPH Radical scavenging activity at 0.03 mg/mL and after 0 minutes.

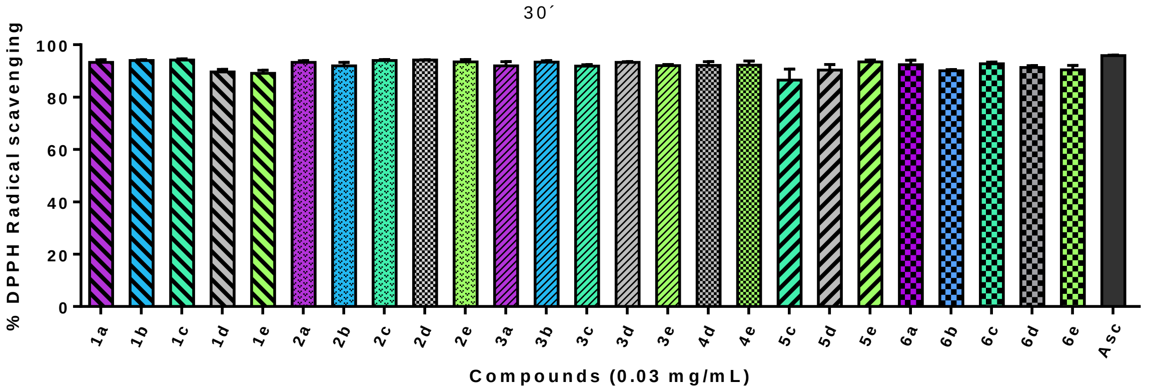

Figure S82. DPPH Radical scavenging activity at 0.03 mg/mL and after 30 minutes.

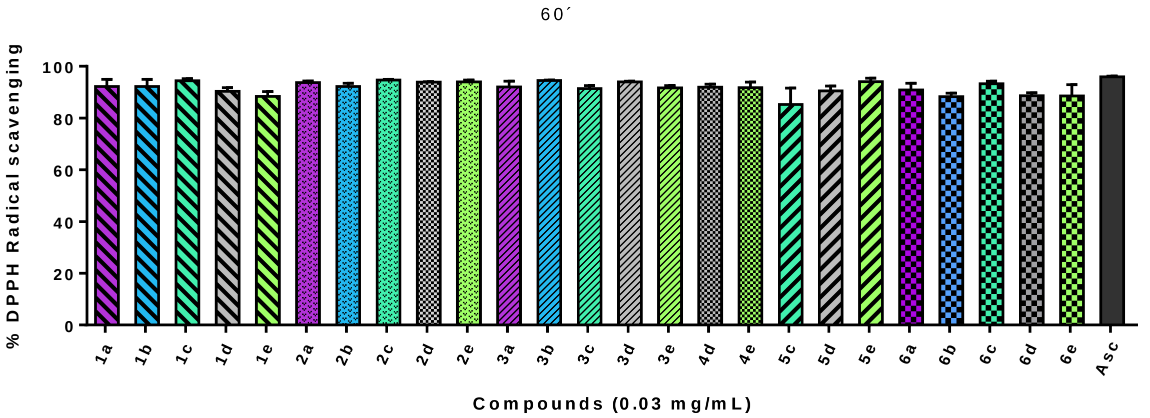

Figure S83. DPPH Radical scavenging activity at 0.03 mg/mL and after 60 minutes.

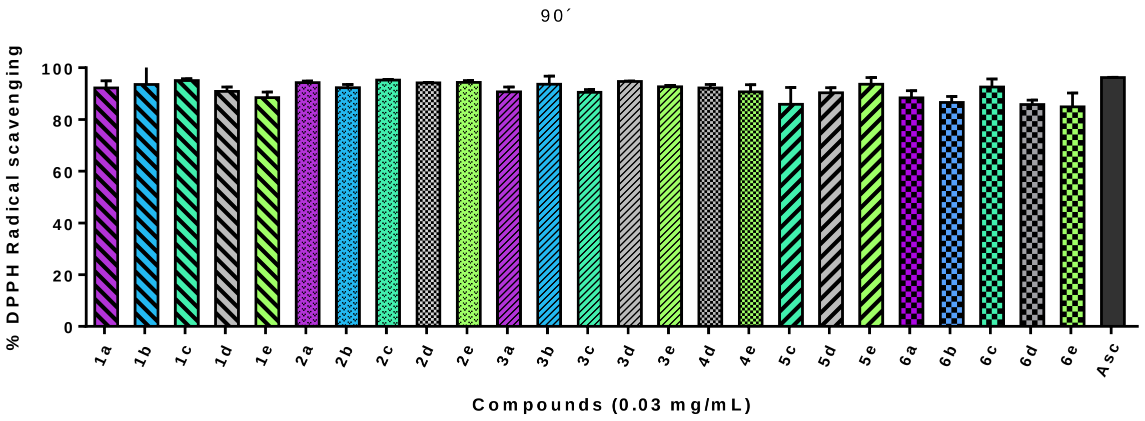

Figure S84. DPPH Radical scavenging activity at 0.03 mg/mL and after 90 minutes.

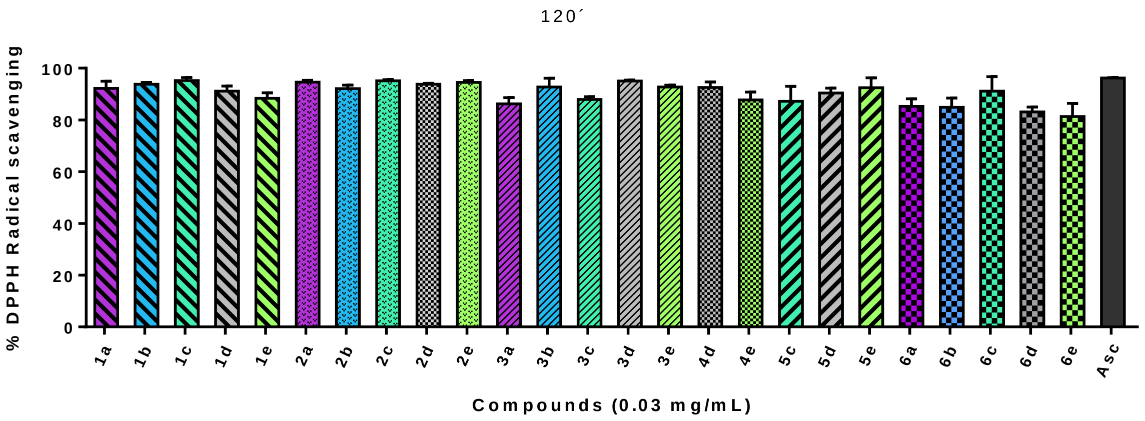

Figure S85. DPPH Radical scavenging activity at 0.03 mg/mL and after 120 minutes.

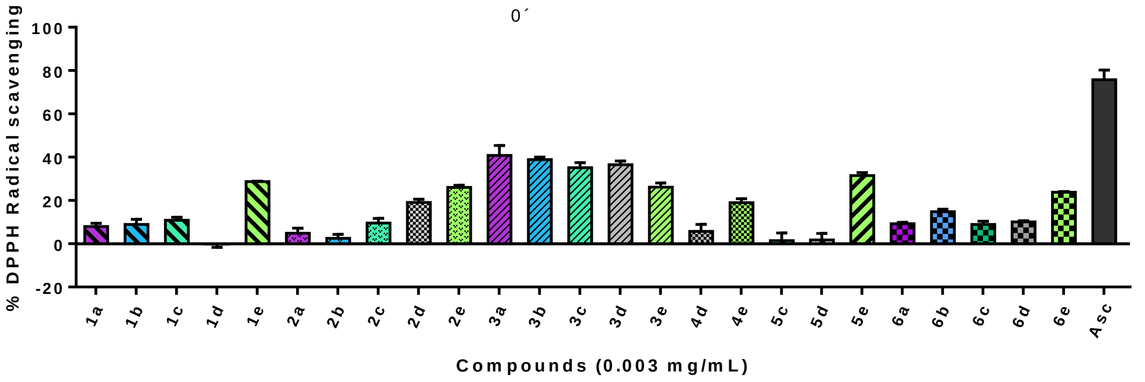

Figure S86. DPPH Radical scavenging activity at 0.003 mg/mL and after 0 minutes.

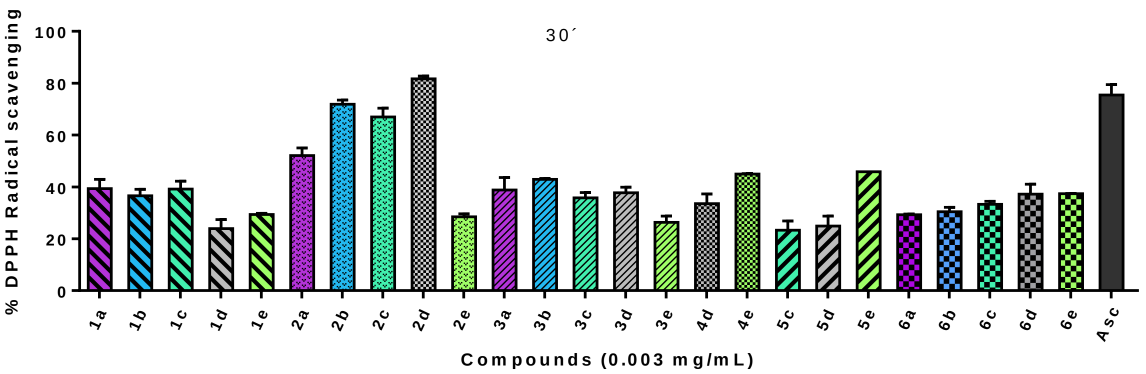

Figure S87. DPPH Radical scavenging activity at 0.003 mg/mL and after 30 minutes.

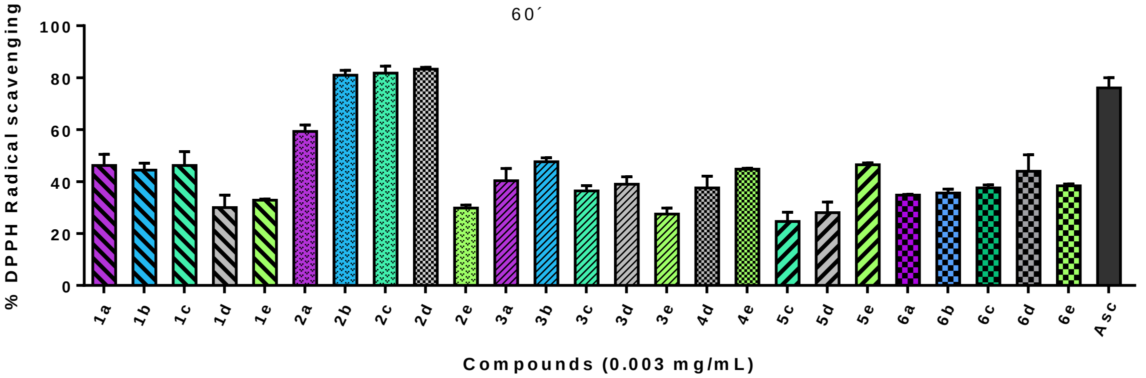

Figure S88. DPPH Radical scavenging activity at 0.003 mg/mL and after 60 minutes.

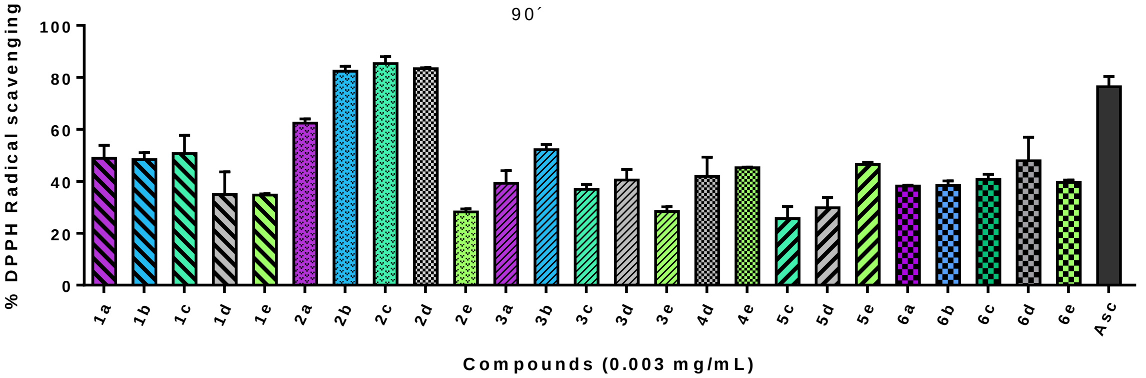

Figure S89. DPPH Radical scavenging activity at 0.003 mg/mL and after 90 minutes.

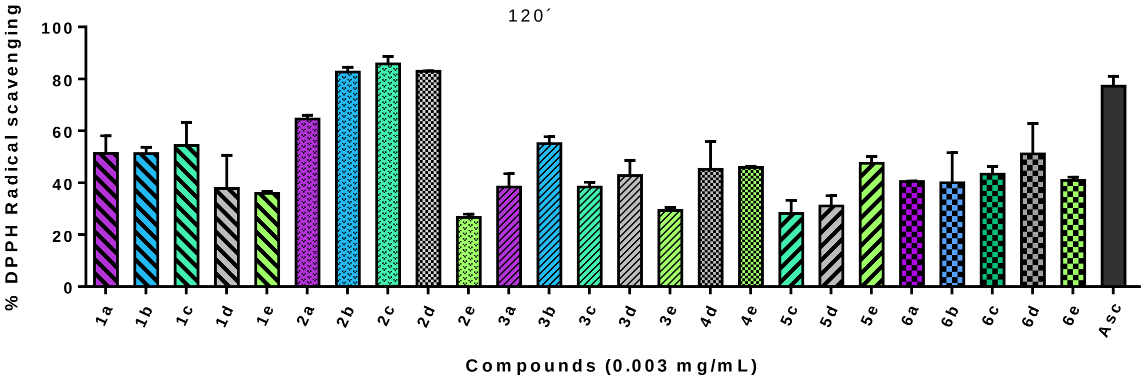

Figure S90. DPPH Radical scavenging activity at 0.003 mg/mL and after 120 minutes.

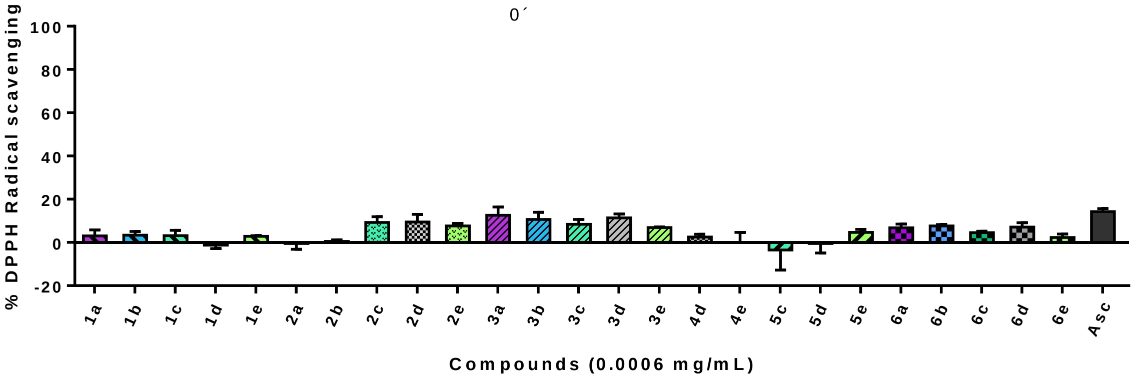

Figure S91. DPPH Radical scavenging activity at 0.0006 mg/mL and after 0 minutes.

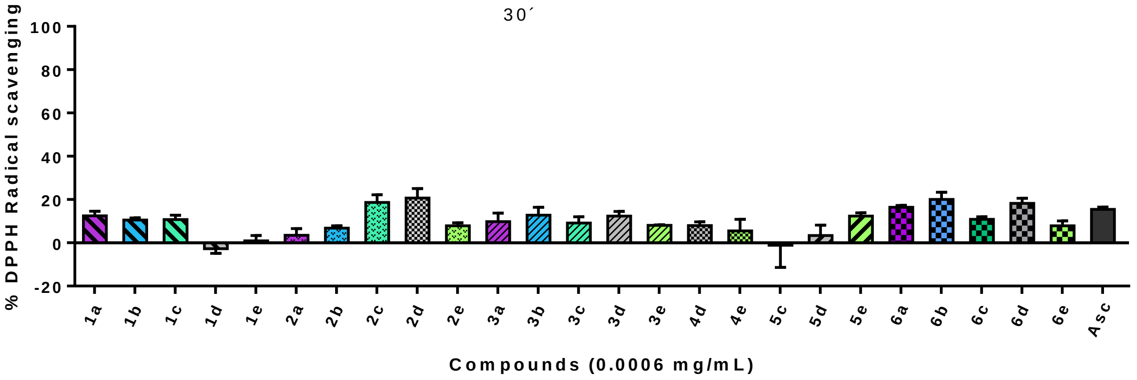

Figure S92. DPPH Radical scavenging activity at 0.0006 mg/mL and after 30 minutes.

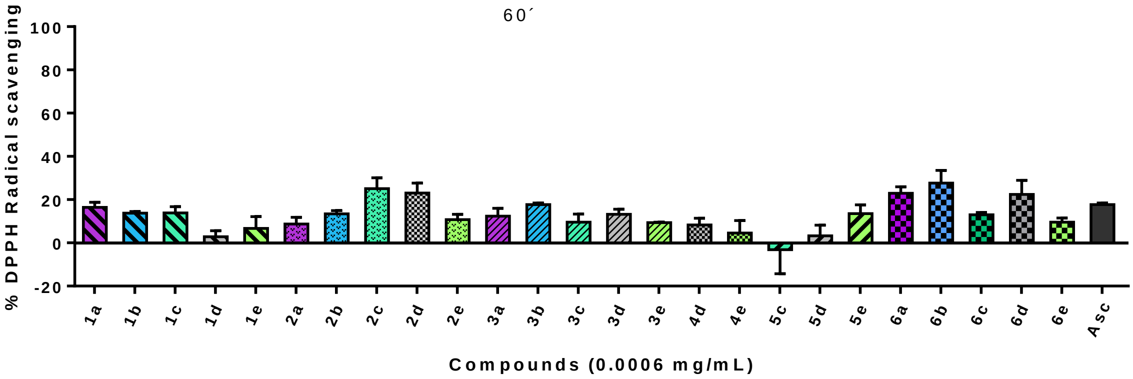

Figure S93. DPPH Radical scavenging activity at 0.0006 mg/mL and after 60 minutes.

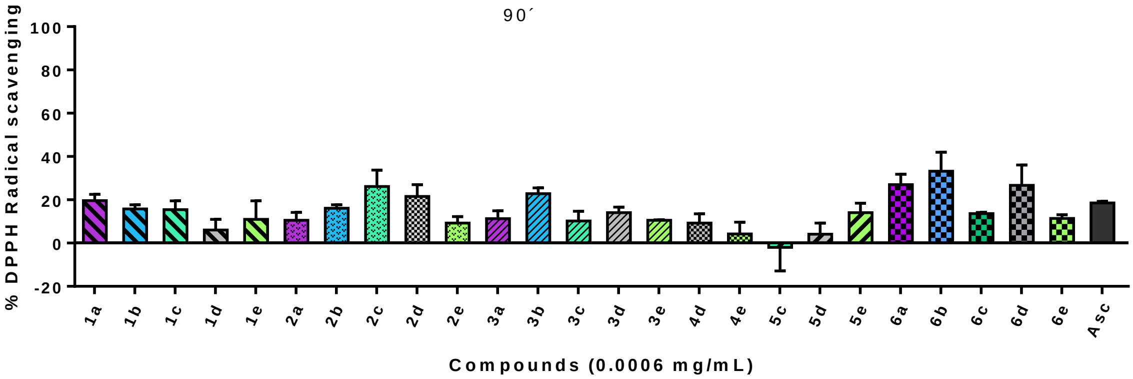

Figure S94. DPPH Radical scavenging activity at 0.0006 mg/mL and after 90 minutes.

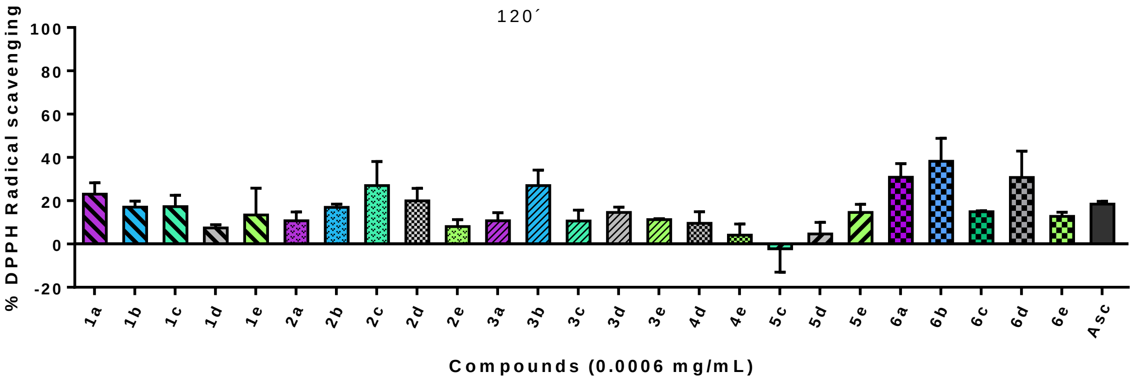

Figure S95. DPPH Radical scavenging activity at 0.0006 mg/mL and after 120 minutes.

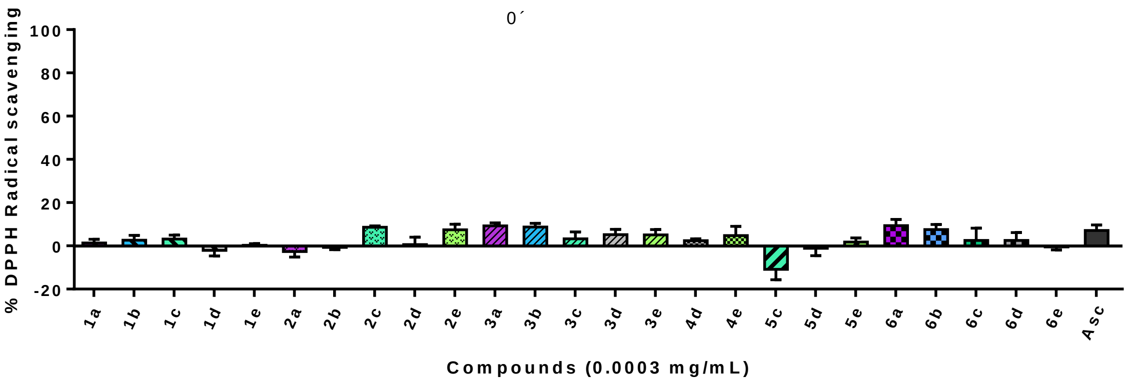

Figure S96. DPPH Radical scavenging activity at 0.0003 mg/mL and after 0 minutes.

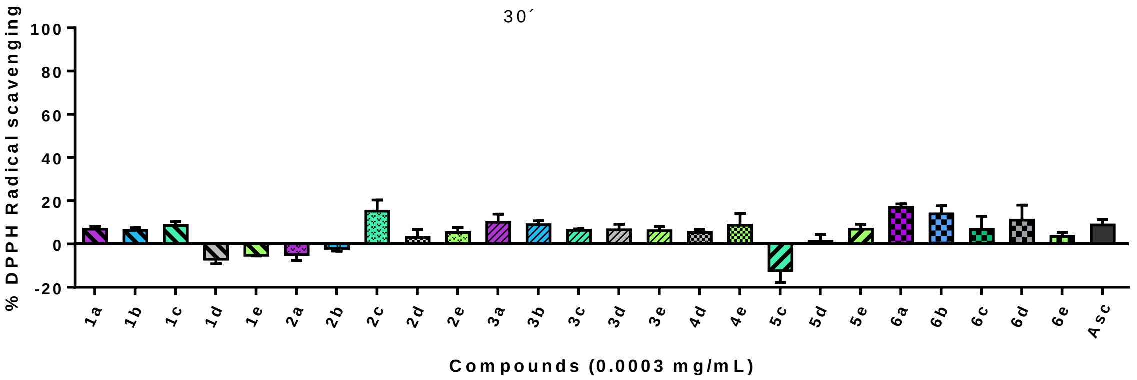

Figure S97. DPPH Radical scavenging activity at 0.0003 mg/mL and after 30 minutes.

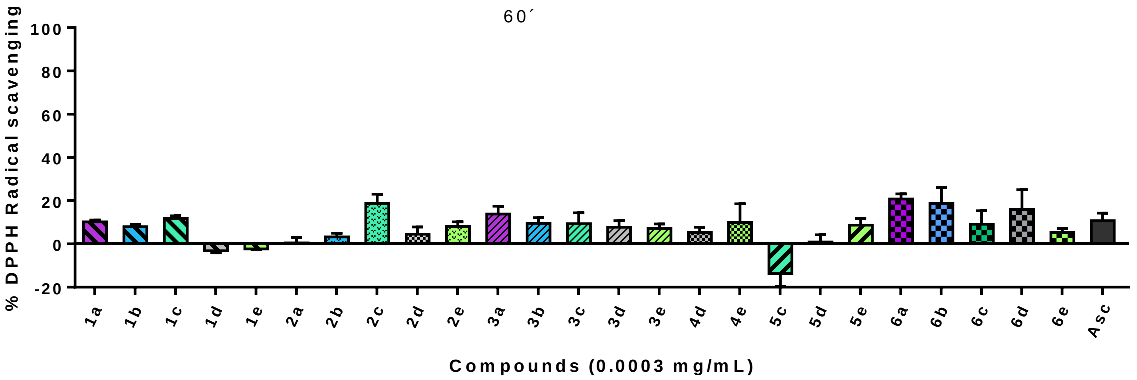

Figure S98. DPPH Radical scavenging activity at 0.0003 mg/mL and after 60 minutes.

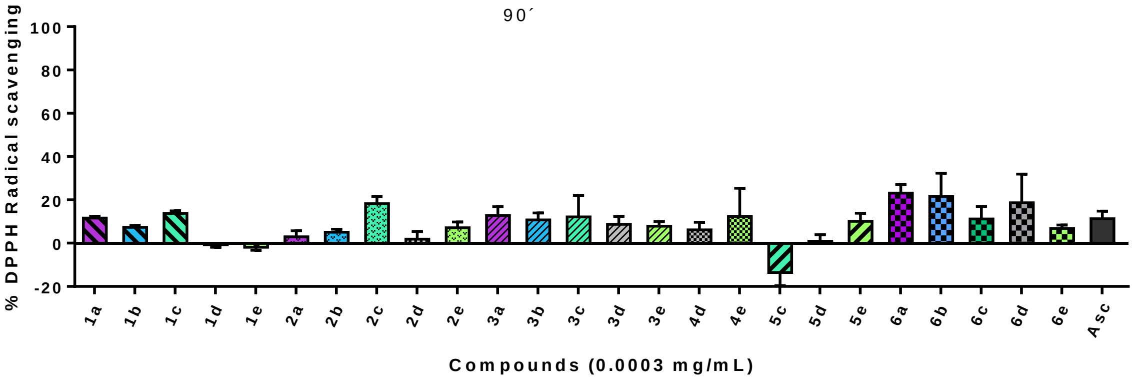

Figure S99. DPPH Radical scavenging activity at 0.0003 mg/mL and after 90 minutes.

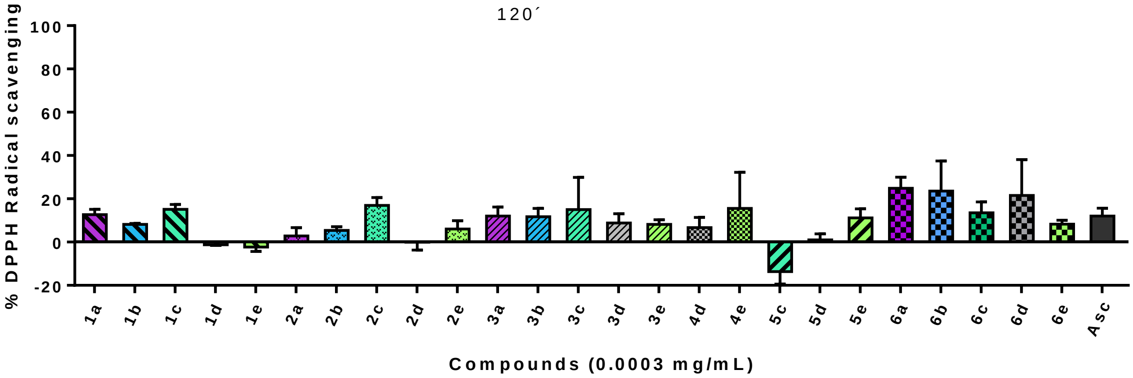

Figure S100. DPPH Radical scavenging activity at 0.0006 mg/mL and after 120 minutes.

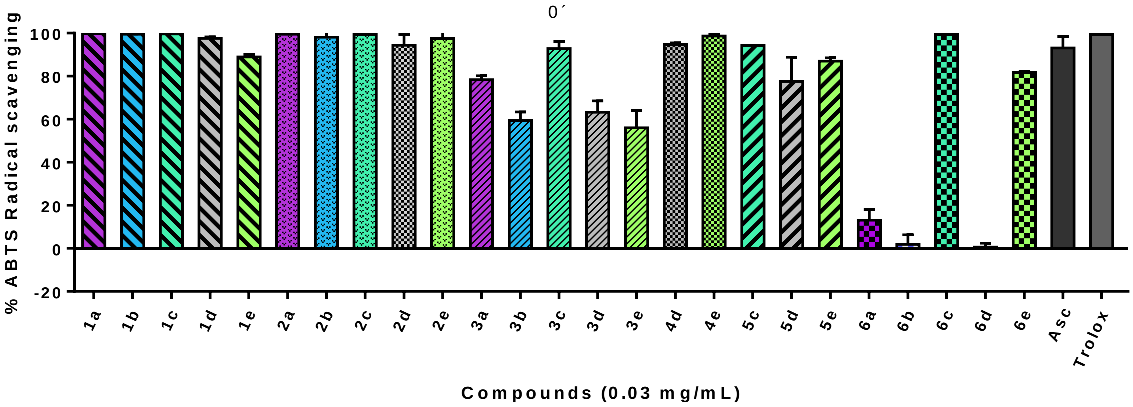

Figure S101. ABTS Radical scavenging activity at 0.03 mg/mL and after 0 minutes.

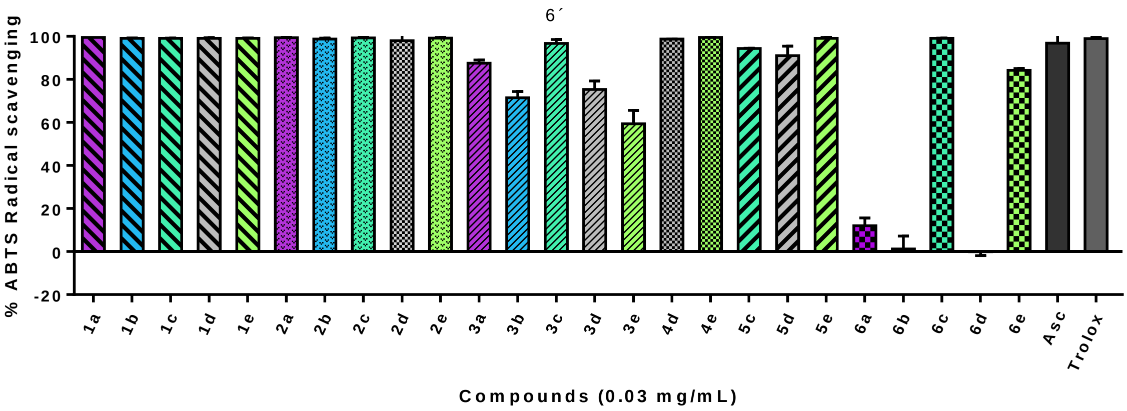

Figure S102. ABTS Radical scavenging activity at 0.03 mg/mL and after 6 minutes.

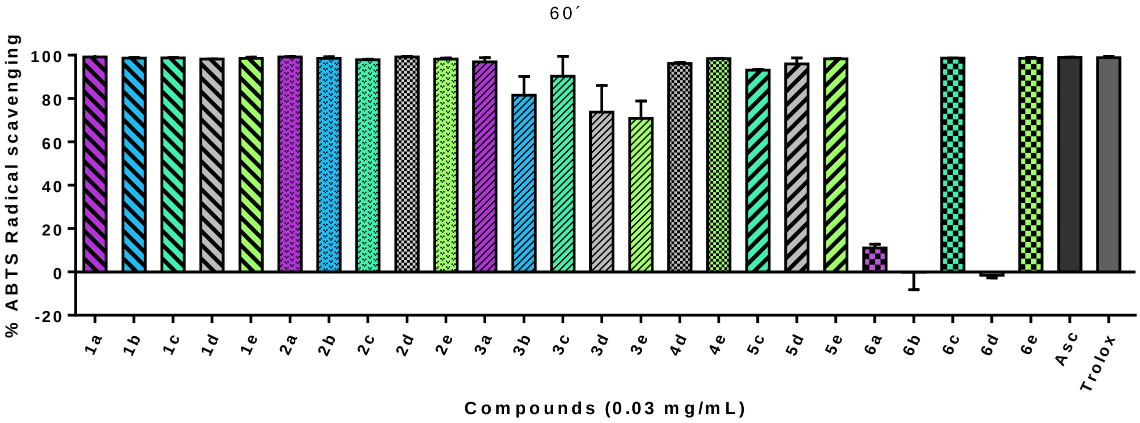

Figure S103. ABTS Radical scavenging activity at 0.03 mg/mL and after 60 minutes.

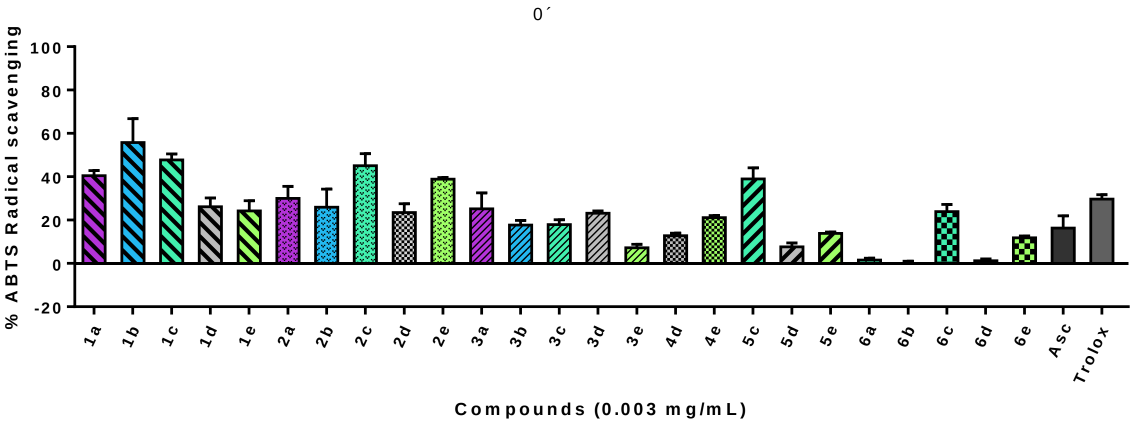

Figure S104. ABTS Radical scavenging activity at 0.003 mg/mL and after 0 minutes.

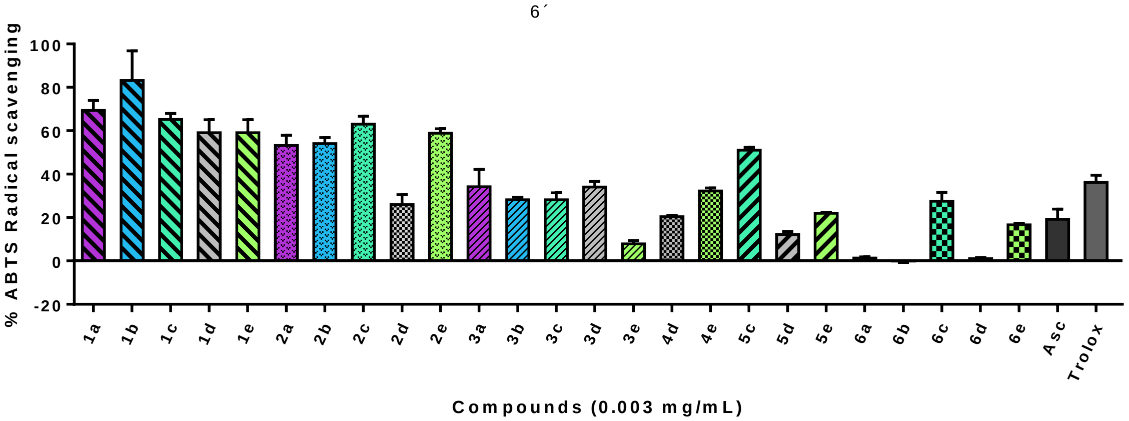

Figure S105. ABTS Radical scavenging activity at 0.003 mg/mL and after 6 minutes.

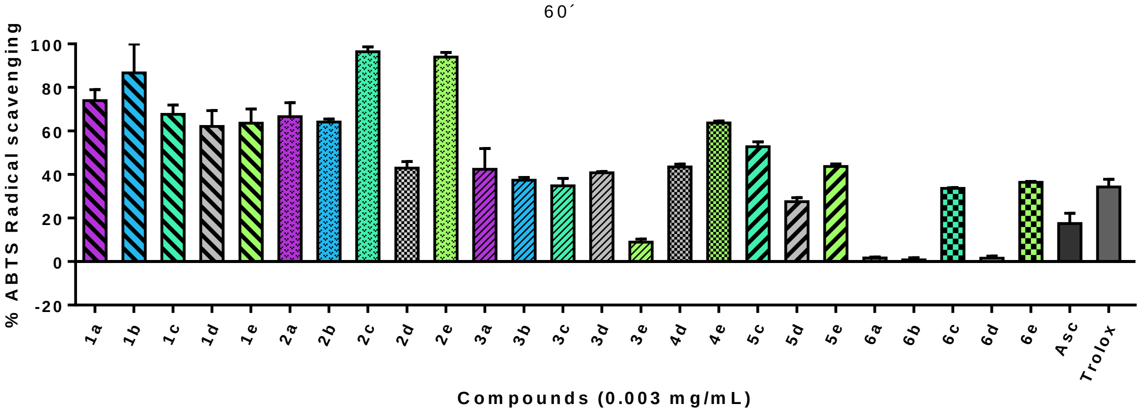

Figure S106. ABTS Radical scavenging activity at 0.003 mg/mL and after 60 minutes.
